# The impact of care farms on quality of life, depression and anxiety among different population groups: A systematic review

**DOI:** 10.1002/cl2.1061

**Published:** 2019-11-26

**Authors:** Jenni Murray, Nyantara Wickramasekera, Marjolein Elings, Rachel Bragg, Cathy Brennan, Zoe Richardson, Judy Wright, Marina G. Llorente, Janet Cade, Darren Shickle, Sandy Tubeuf, Helen Elsey

**Affiliations:** ^1^ Academic Unit of Public Health, Leeds Institute of Health Sciences University of Leeds Leeds UK; ^2^ Plant Research International Wageningen University Wageningen The Netherlands; ^3^ Care Farming UK Bedminster UK; ^4^ York Trials Unit, Department of Health Sciences, Faculty of Science University of York York UK; ^5^ Leeds Institute of Health Sciences The University of Leeds Leeds UK; ^6^ Madrid Institute for Rural, Agricultural and Food Research and Development (IMIDRA), Social‐Ecological Systems Lab, Ecology Department Universidad Autónoma de Madrid (Spain) Madrid Spain; ^7^ University of Leeds Leeds UK

## Abstract

Care farming (also called social farming) is the therapeutic use of agricultural and farming practices. Service users and communities supported through care farming include people with learning disabilities, mental and physical health problems, substance misuse, adult offenders, disaffected youth, socially isolated older people and the long term unemployed. Care farming is growing in popularity, especially around Europe. This review aimed to understand the impact of care farming on quality of life, depression and anxiety, on a range of service user groups. It also aimed to explore and explain the way in which care farming might work for different groups. By reviewing interview studies we found that people valued, among other things, being in contact with each other, and feeling a sense of achievement, fulfilment and belonging. Some groups seemed to appreciate different things indicating that different groups may benefit in different ways but, it is unclear if this is due to a difference in the types of activities or the way in which people take different things from the same activity. We found no evidence that care farms improved people's quality of life and some evidence that they might improve depression and anxiety. Larger studies involving single service user groups and fully validated outcome measures are needed to prove more conclusive evidence about the benefits of care farming.

## 1 PLAIN LANGUAGE SUMMARY

### 1.1 More evidence needed on the effectiveness of care farms (CFs)

Care farming is the therapeutic use of agricultural and farming practices. People value the farms, but the evidence on their effectiveness is limited.

### 1.2 What is this review about?

Care farming (also called social farming) is the therapeutic use of agricultural and farming practices. Service users and communities supported through care farming include people with learning disabilities, mental and physical health problems, substance misuse, adult offenders, disaffected youth, socially isolated older people and the long‐term unemployed.

This review aims to understand the impact of care farming on quality of life, depression and anxiety, on a range of service user groups. It also aims to explore and explain the way in which care farming might work for different groups.

**What is the aim of this review?**

This Campbell systematic review examines the impact of care farming on quality of life, depression and anxiety, on a range of service user groups. It also aims to explore and explain the way in which care farming might work for different groups.

### 1.3 What studies are included?

The review included randomised controlled trials (RCTs) and quasi‐RCTs; interrupted time series and nonrandomised controlled observational studies; uncontrolled before and after studies and qualitative studies. Study participants were those who typically receive support at a CF. Studies conducted in a setting that met the accepted definition of a CF were included, but farming interventions that were carried out in a hospital or prison setting were excluded.

The total number of included studies in this review are 18 qualitative studies and 13 quantitative studies, one of which was a mixed‐methods study.

### 1.4 What are the findings of this review?

The qualitative interview studies showed that people valued, among other things, being in contact with each other, and feeling a sense of achievement, fulfilment, and belonging.

Some groups seemed to appreciate different things, indicating that different groups may benefit in different ways but, it is unclear if this is due to a difference in the types of activities or the way in which people value different things from the same activity.

There is a lack of quantitative evidence that CFs improve people's quality of life, but some evidence that they might improve depression and anxiety.

Larger studies involving single service user groups and fully validated outcome measures are needed to prove more conclusive evidence about the benefits of care farming.

### 1.5 What do the findings of the review mean?

There is a lack of evidence to determine whether or not care farming is effective in improving quality of life, depression and anxiety. More evidence is available for those with mental ill‐health, but firm conclusions cannot be drawn.

Despite the current lack of robust evidence to support the effectiveness of care farming, there are strong arguments to support a more integrated approach to care farming as a viable alternative or adjunct to mainstream approaches for mental health problems. Lack of choice, gender inequalities, and over‐burdened statutory services indicate the need for a credible alternative treatment option.

There needs to be a concerted effort to increase awareness among commissioners of health care, frontline service providers and potential service users about care farming, how—and for whom—it might work. Models across Europe that offer a more integrated approach between green care and statutory services could provide valuable learning.

The evidence for care farming for other service user groups is not as well developed as it is for those with mental health problems, but that is not to say there is not a need. Disaffected youth, adult offenders and people with dementia represent significantly large vulnerable population groups where current service provision struggles to meet demand.

The need to continue to improve and provide high quality research in these areas is therefore pressing.

### 1.6 How up‐to‐date is this review?

The review authors searched for studies published up to July 2017.

## 2 EXECUTIVE SUMMARY

### 2.1 Background

Care farming (also called social farming) is the therapeutic use of agricultural and farming practices. Service users and communities supported through care farming include people with learning disabilities, people with mental and physical health problems, people with substance misuse problems, adult offenders, disaffected youth, socially isolated older people and the long‐term unemployed. Care farming is a highly complex intervention that can involve different farming activities (horticulture, forestry or livestock farming) or other activities (gardening, conservation or woodwork), with different levels of support provided according to the needs of the individual service users. Likewise the service users can contribute to farming production or the farm itself may focus on the provision of care services. Care farming sits within a broader framework of similar nature based supportive interventions collectively terms green care that also includes wilderness therapy, social and therapeutic horticulture, environmental conservation and green exercise. There are around 1,100 CFs in The Netherlands, 900 in France, 675 in Italy and 669 in Belgium. In the UK and Ireland (both the Republic and the North) numbers are fewer with around 230 and 100, respectively. With increasing pressure on the health and social care sector, commissioners are turning to green care interventions as an alternative approach. Although a number of overviews and one systematic review of care farming exists there is a need for a review that captures the full range of published and grey literature, and to explore in depth the mechanisms that explain how care farming works for different service user groups.

### 2.2 Objectives

The primary objective was to systematically review the available evidence of the effects of CFs on quality of life, health and social well‐being on service users. Within this, we aimed to explore the size of the effect that CFs have on the health, well‐being and social outcomes of different population groups. With available material we also aimed to explore the relationship between contextual data (the activities and characteristics of the farm and the nature of the service user groups) and the impact on outcomes. Finally, we aimed to understand the mechanisms of change for different population groups with a view to constructing a logic model to describe the ways in which care farming might work.

### 2.3 Search methods

In 2015, we searched 22 health, education, environmental, criminal justice and social science electronic databases. We also searched databases of grey literature, and various websites, including care farming websites across a number of European countries. Reference lists of included studies and identified systematic reviews were scanned, and citations of key papers were tracked using Google Scholar and Web of Science Citation Indices. This was supplemented by hand searching the Wageningen Journal of Life Sciences from 2000 onwards and by contacting academic and care farming networks to identify any other reports. Our search terms were deliberately broad to capture all rehabilitative interventions occurring on farm and farm type settings. The search of electronic databases as repeated in 2017, due to limited resources the grey literature search was not repeated in 2017.

### 2.4 Selection criteria

We included a broad range of study designs: RCTs and quasi‐RCTs; interrupted time series and nonrandomised controlled observational studies; uncontrolled before and after studies and qualitative studies. We excluded single subject designs, reviews, overviews, surveys, commentaries and editorials. Study participants were those that typically receive support at a CF, including but not restricted to people with mental health problems, learning difficulties, health problems, substance misuse problems, and offenders and disaffected youth. Only those attending for a single day as a visitor were excluded. Studies conducted in a setting that met the accepted definition of a CF were included, but farming interventions that were carried out in a hospital or prison setting were excluded. For the purposes of developing the logic model, we retained papers that described any theories to explain how and for whom care farming might work. These papers are not formally included in the review.

### 2.5 Data collection and analysis

Each screening stage involved two independent reviewers. Studies that were potentially eligible after title and abstract screening underwent full paper screening. Disagreements were discussed and resolved by consensus at each stage. Papers describing theories in relation to care farming were separately retained even if they did not meet the inclusion criteria for the purposes of constructing a theoretical framework to inform the logic models. The Preferred Reporting Items for Systematic Reviews and Meta‐Analysis (PRISMA) was used to state the process of study selection. We stored all references in Endnote (VX7) and recorded extracted data and the outcomes of full paper screening in EPPI‐Reviewer 4 (V.4.5.0.1). The data extraction form was based on the CPHG Data Extraction and Assessment Template with subsections for contextual information, and qualitative and quantitative data. We used a sequential exploratory approach to the review involving four stages: (a) developing a theoretical framework; (b) identifying the intervention components, mechanisms of change, and proximal outcomes from existing theories and qualitative data; (c) mapping the mechanisms of change and proximal outcomes to the theories to develop the logic models and (d) testing the logic models against the quantitative data. We used an adapted version of the COREQ tool to assess the quality of the qualitative studies, and the EPOC and EPHPP tools to assess the risk of bias in quantitative studies. No studies were excluded based on quality. The nature of the studies meant that we were unable to assess treatment effect and reporting biases.

### 2.6 Results

In 2015, our search methods identified 1,659 articles, of which 14 qualitative studies, 13 quantitative studies and one mixed methods study met the inclusion criteria. In addition, we identified 15 theories that had been quoted in connection with care farming. The rerun of the search of publish literature in July 2017 identified a further 391 articles, of which three qualitative studies met the inclusion criteria. The total studies in this review are 18 qualitative studies and 13 quantitative studies, one of which was a mixed‐methods study. We created four logical models to explain how care farming might work: an overall one for all service user groups; one for people with either mental health problems or substance misuse problems, one for disaffected youth and one for people with learning disabilities. These models comprised five key theoretical concepts derived from identified theories (restorative effects of nature, being socially connected, personal growth, physical well‐being and mental well‐being), five categories of intervention components (being in a group, the farmer, the work, the animals and the setting) and 15 categories of mechanisms derived from included qualitative studies (achievement and satisfaction, belonging and nonjudgement, creating a new identity, distraction, feeling valued and respected, feeling safe, learning skills, meaningfulness, nurturing, physical well‐being, reflection, social relationships, stimulation, structure, and understanding the self). In addition, from the theories and qualitative studies, we identified 12 different outcomes, both proximal (secondary) and primary, that we expected to find when testing the logic models against the quantitative studies. One key theoretical concept “restorative effects of nature” was underrepresented in the intervention components and mechanisms reported within the qualitative studies. The types of mechanisms appeared to differ according to different service user groups, suggesting that care farming may work in different ways according to different needs. Across the 13 quantitative studies (including the mixed methods study), 24 different outcome measures were reported. Eight studies (both qualitative and quantitative) reported results for mixed client groups. Only the logic model for mental illness and substance misuse was tested, due to a lack of quantitative evaluations for the other service user groups. We found a lack of evidence to indicate that CFs improve quality of life, and limited evidence that they might improve depression and anxiety. There was some evidence to suggest that CFs can improve self‐efficacy, self‐esteem and mood, with inconsistent evidence of benefit for social outcomes. All of the studies had a high risk of bias so the results should be treated with caution.

### 2.7 Authors’ conclusions

There is a lack of evidence available to determine whether or not care farming is effective in improving quality of life, depression and anxiety. More evidence is available for those with mental ill‐health, but firm conclusions cannot be drawn. Small study sizes of poor design, evaluations involving mixed service user groups, the use of multiple and sometimes unvalidated outcome measures, short follow‐ups, and the absence of key outcomes that fit with theory have all hampered the development of a more robust evidence base. However, we now have a set of theory‐based logic models that offer a framework for research evaluations. With recommendations in place to address the current research inadequacies there is an opportunity to vastly improve the evidence base for care farming.

Despite the current lack of robust evidence to support the effectiveness of care farming, there are strong arguments to support a more integrated approach to care farming as a viable alternative or adjunct to mainstream approaches for mental health problems. Lack of choice, gender inequalities and over‐burdened statutory services indicate the need for a credible alternative treatment option. A concerted effort to increase awareness among commissioners of health care, frontline service providers, and potential service users about care farming, how, and for whom, it might work is needed. Models across Europe that offer a more integrated approach between green care and statutory services could provide valuable learning. The evidence for care farming for other service user groups is not as well developed as it is for those with mental health problems, but that is not to say there is not a need. Disaffected youth, adult offenders and people with dementia represent significantly large vulnerable population groups where current service provisions struggles to meet demand. The need to continue to improve and provide high quality research in these areas is, therefore, pressing.

## 3 BACKGROUND

### 3.1 The problem, condition or issue

Supporting individuals whose vulnerabilities put them at greater risk of poorer quality of life is a cornerstone of many charitable/third sector organisations. Often the support needed goes beyond that which can be provided by statutory health and social care organisations. This is partly a capacity issue relating to increasing life expectancies over the 20th century (Christensen, Doblhammer, Rau, & Vaupel, 2009) and increasing prevalence of long‐term conditions. However, it also relates to changing needs and demands of populations within modern societies. Many of the problems presenting to health service providers are complex and are often underpinned or exacerbated by social problems such as poor education, poor housing, unemployment and social isolation, and the skills within health services to address these issues do not exist within this sector (Citizen's Advice, 2015; Popay, Kowarzik, Mallinson, Mackian, & Barker, 2007). Thus, for many such individuals inadequate support can lead to poorer quality of life, and for society as a whole, greater health inequalities (Marmot et al., 2010).

Learning disabilities is an umbrella term for a range of conditions, including Down's syndrome, fragile X syndrome and cerebral palsy. Autism spectrum disorder (ASD) can also be included here, but not all people with ASD have a learning disability. People with learning disabilities^1^ often experience poorer health and higher mortality due to increased social and health inequalities and underlying pre‐existing conditions (Krahn, Hammond, & Turner, 2006). Although many people with learning difficulties could reach more personal autonomy through the labour market, rates of employment are very low and social isolation is common (www.mencap.org.uk). Further, some conditions are associated with varying degrees of challenging behaviours so placement for some individuals can be difficult. Day care is available for people with learning disabilities, but ensuring that people are given a sense of purpose alongside social interaction in a place without judgement can be a challenge.

Mental illnesses, including, for example, depression, anxiety, personality disorders, schizophrenia and posttraumatic stress disorders are a leading cause of disability in the occidental cultures (Murray et al., 2012). In some countries, such as the UK, the prevalence of major depression is increasing and imposing huge personal and economic costs (Centre for Mental Health, 2010). Likewise in Spain, although indicators of physical health have constantly improved during the last three decades, indicators of healthy habits (rates of cholesterol, diabetes, hypertension, allergies and obesity) and mental health (such as the number of suicides and the number of psychological treatments) have worsened (Spanish National Ecosystem Assessment, 2013). As an early treatment, approximately 70% of depressed patients in UK primary care are prescribed antidepressant medication (Kendrick, Stuart, Newell, Geraghty, & Moore, 2015); however, adherence may be as low as one third (Bull et al., 2002). An alternative or adjunct to antidepressants is talking therapies. There are long waiting lists, and of those that complete the course around two thirds show signs of improvement and 40% recover (Department of Health, 2012). But for the many that do not take up the offer of talking therapies or who do not benefit from it, there are few alternatives. Social problems can also underpin many anxiety and depressive disorders. A more practical approach that directly targets these underpinning causes may be a more effective approach and an efficient use of resources. Providing a safe calm environment that is nonconfrontational and offers structure and space for people to channel their energies into tasks that are mentally relaxing could provide a good fit for those who are unable to benefit from more conventional services.

Disengaged or disaffected children, defined as those who are not fully taking part in school life as they have given up trying or are resisting help (Lumby, 2013), are at high risk of exclusion from school. Exclusion from school can predispose young people to becoming a “NEET” (a person between the age of 16 and 24 and Not in Education, Employment or Training), which in turns carries an increased likelihood of committing a criminal offence, being in a lower paid job and subsequently a poorer quality of adult life compared to those who complete their education (Audit Commission, 2010; Public Health England, 2014). Evidence suggests that the numbers of children that fit within the disengaged category are increasing (McEwan et al., 2014; Robins, Cohen, Slomkowski, & Robins, 1999), and a large proportion of youth who show problem behaviour at a young age go on to develop antisocial personality disorders as an adult (Rutter et al., 1997) or can experience social exclusion (Hassiotis & Hall, 2008). Furthermore, there is also an increased risk of developing psychoactive substance use disorders, bipolar disorder and long‐term smoking addictions (Biederman et al., 2008). Strategies to support children and young people in this situation are in place across a number of developed countries. For example, in the UK schools can refer pupils at risk of exclusion directly to off‐site educational provisions. These can include local CFs which are contractually obliged to support teenagers to achieve National Open College Network accreditation. Importantly, classroom‐based education is integrated with practical outdoor activities, which enables better student engagement.

Offenders often have mental and physical health problems (Brooker, Syson‐Nibbs, Barrett, & Fox, 2009) or drug addiction and substance misuse problems (Abracen, Looman, & Anderson, 2000), and are more likely to have suffered from socioeconomic deprivation (Farrington, 1990), to have witnessed domestic violence (Caputo, Frick, & Brodsky, 1999), to have a family history of criminal violence (Farrington & West, 1990) or to have experienced harsh or neglectful parenting (Sutton, Utting, & Farrington, 2004). Poor education and a lack of skills predisposes individuals to unemployment, which itself is a risk factor for offending (Farrall, 2013). Some CFs aim to support offenders by developing self‐esteem and providing work‐based skills that provide hope for the future.

Being able to be physically active in nature may help to improve both the physical and mental well‐being of older people (Elings, Haubenhofer, Hassink, Rietberg, & Michon, 2011). Levels of depression and anxiety are often higher among these groups than the general populations (Pedersen et al., 2011), and findings suggest that depression can cause worse health outcomes in older people when combined with chronic conditions such as arthritis, asthma or diabetes (Moussavi et al., 2007).

### 3.2 The intervention

#### 3.2.1 Defining care farming

Care farming (also called social farming) has been formally defined as the use of commercial and noncommercial farms and agricultural landscapes as a base for promoting mental and physical health, through normal farming activity (Hassink, 2003; Hassink & Van Dijk, 2006; Hine, Peacock, & Pretty, 2008a). A CF utilises the whole or part of a farm to provide health, social or educational care services, employment skills and support for different groups of people, through the provision of a supervised, structured programme of farming‐related activities, rather than occasional one‐off visits (Care Farming UK, 2016; Di Iacovo & O'Connor, 2009).

Care farming is a truly complex intervention. It may occupy part of a farm where farming production is the primary function (i.e., commercial agricultural units), or where the main function is provision of care services (i.e., community farms). Farms also differ in the types of farming activities undertaken (e.g., horticulture, forestry and livestock farming), other activities available (e.g., gardening, composting organic waste, medicinal plants work, conservation and woodwork), the level of support provided (e.g., health promotion, counselling, rehabilitation and skills qualifications) and the range of service user groups treated. Given this complexity, the main defining feature of a CF is the involvement in agrarian or forestry activities for a therapeutic purpose. It is also important to highlight the farming component of the intervention, as this helps to distinguish care farms from horticultural or animal‐based therapy projects. Care farms can function as a social enterprise where income gained by agricultural production is used to finance the CF (Elings et al., 2011).

- A diverse range of activities can be offered to service users at a care farm. Tasks selected are primarily determined by the particular needs and capabilities of the user and the type of farm and activities available. The range of CF types varies both between and within countries across Europe. To demonstrate the variety of care farms in the UK, the Netherlands and Spain, some examples are provided below and further details can be found from the following websites: Care Farming UK (https://www.carefarminguk.org/) and from European social farming sites: http://www.maie‐project.eu/index.php?id=33; http://www.socialfarmingacrossborders.org/seupb; http://www.egina.eu/. In Madrid, a city farm with an urban orchard offers occupational activities and training for employment to people with learning disabilities. Among other activities they do horticultural work and raise livestock. The farm includes a one hectare urban orchard divided into 200 smaller areas that are rented to the general public. People with learning disabilities help clients to take care of their orchards and provide them with advice and support to keep orchards in a good condition. In addition, they attend school.
- In a farm in the North West of England the service users are primarily those with mental health problems, and activities are focused on horticultural production, but with some site maintenance. The service users also cook meals for themselves on site, often using produce that they have grown onsite. Service users are given work that increases in intensity as they recover. Working within a therapeutic community is the essence of the farm.
- In the Midlands, a farm supports offenders who are unemployed and have social problems. The farm provides a range of activities including growing vegetables, harvesting and retailing produce, and working with the farmer to manage a large herd of beef cattle. Since an aim is to improve employability, offenders are awarded a nationally recognised qualification on completion of their time at the farm. Because offenders work with the same group throughout their time at the farm, they have the opportunity to develop friendships.
- A city farm based in London runs a project on site which aims to reduce social isolation for older people living in residential homes and those using the services of older people's organisations. They specifically offer animal handling, which not only gives individuals an opportunity to touch and care, but also creates an avenue for open conversations to encourage social engagement.
- In the southern part of the Netherlands, a farmer and his wife (who works in health care) run a small scale CF with cows and arable produce. The farmer's wife provides day activities for people with learning difficulties and mental health problems. On average, eight service users access the farm each day, working together on different activities. They have coffee and lunchbreaks together with the family. Some of the service users work in the farm shop.

In addition to engaging in different activities, a small number of care farms offer service users the opportunity to interact with other professional caregivers to receive counselling or support to develop a healthier lifestyle. A recent survey of care farms in England found that, on average, 34 participants attended a CF each week. The length and duration of the CF intervention is determined by the need of the client, and this varies from one to three times a week, on average over a period of 30 weeks (Bragg, Egginton‐Metters, Elsey, & Wood, 2014).

In addition, the intervention can vary depending on the wider cultural context in which the farm resides. For example, in the Netherlands, an agriculturally productive farm will offer some form of care or health promotion to their service users, whereas in Germany, care farms are frequently connected to a healthcare institution rather than being solely based on agricultural production farm (Haubenhofer, Elings, Hassink, & Hine, 2010). German care farms often function on a large scale, as government subsidies are only provided to farms with more than 300 service users (Haubenhofer, Blom‐Zandstra, Kattenbroek, & Brandenburg, 2010).

The service users that utilise care farms also differ according to the setting of the intervention, for example, in Norway, the service users tend to be young children and psychiatric patients, whereas in the United Kingdom, Belgium, the Netherlands and Italy, a variety of different people use the intervention (Haubenhofer et al., 2010).

Individuals and communities supported through care farming include those with learning difficulties, ASD or mental health problems, plus disaffected youth, people with physical disabilities, older people, people with drug and alcohol problems, adult offenders, people with dementia, and exservice personnel (Bragg et al., 2014). In the UK, the largest service user groups are those with learning difficulties, ASD, mental health problems and disaffected youth. A similar pattern of intake is seen in the Netherlands, the country with the greatest number of care farms. In developing countries and areas experiencing greater rural poverty, care farming is also now being used to support the long‐term unemployed and empower women to become economically active (Food and Agriculture Organization, 2015).

Care farms can also provide support for offenders referred from probation services either as a rehabilitative intervention or as a way of “paying‐back” to the community for crimes committed (Elsey et al., 2018). Elderly people, including those with dementia, are a more recent group to use care farming (Elings et al., 2011). Care farms can offer an alternative to day centres by providing a home from home environment that can involve some outdoor work for mental stimulation and physical activity.

The number of care farms has been growing, particularly in Europe, with an estimated 1,100 care farms now in the Netherlands (Elings et al., 2011), and around 230 in the UK (Bragg & Atkins, 2016), 900 in France, 669 in Belgium (Steunpunt Groene Zorg, 2014), 160 in Germany, 675 in Italy, 100 in Ireland (Di Lacovo & O'Connor, 2009) and around 40 in Catalonia in Spain (Guirado‐González et al., 2014).

#### 3.2.2 Care farming within the broader literature

**Figure 1:**
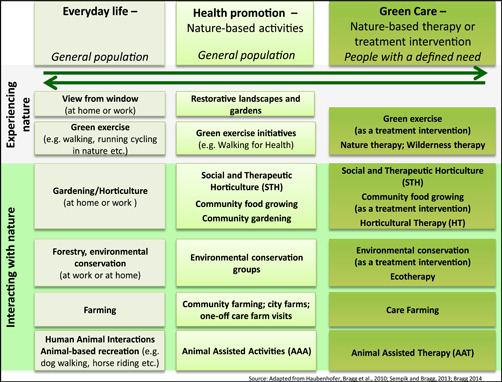
The different contexts in which an individual may engage with nature. *Source*: (Bragg & Atkins, 2016). The three columns represent the different contexts in which an individual may engage with nature. On the left, the “Everyday life” column highlights various situations in which an individual engages with nature as part of their normal lifestyle. The middle column “Health promotion” outlines a variety of existing group projects and initiatives which aim specifically to encourage individuals, communities and disadvantaged groups to benefit from nature‐based activities. Funding is usually for the project as a whole and may come from public health, local authority grants or from the voluntary or private sector. On the right, the “Green care” column represents the various nature‐based interventions which have been specifically commissioned for an individual with a defined health or social need as part of their care or treatment package

The ways in which individuals interact with nature can be viewed as a continuum with overlapping categories, ranging from general everyday contact such as viewing, working or undertaking recreational activities, through to using nature deliberately as a therapeutic or treatment resource (i.e., green care) involving activities like wilderness therapy, social and therapeutic horticulture, animal‐assisted therapy and care (social) farming (see Figure 1). Green care has been defined as follows:

Green care utilises plants, animals and landscapes to create interventions to improve health and well‐being; (i.e., it does not represent a casual encounter with nature). It also provides care and support to enable people to reach their true potential, that is, although many of the approaches are termed “therapies” or “therapeutic”, they are not necessarily directed at treating or curing conditions and diseases but, as in the case of people with learning difficulties, for example, they provide care, support, training and other opportunities to enable those individuals to develop. Such opportunities are often not available elsewhere (Sempik & Bragg, 2013).

Care farming is a distinct category within green care as the focus is on the use of a farm, either a commercial farms or other agricultural landscapes as a base for promoting mental and physical health, through normal farming activity (Hassink, 2003; Hassink, Zwartbol, Agricola, Elings, & Thissen, 2007; Hine, 2008). Activities are not designed as “therapy” as they might be within a horticultural therapy or animal‐assisted therapy, rather they are the jobs that would need to be done on a farm to ensure successful production. Furthermore, care farms provide a range of farming activities that users can engage with. This provides a clear distinction with therapeutic horticultural activities and animal‐assisted interventions (AAI) which focus on a single activity such as gardening or horse riding.

### 3.3 How the intervention might work

As a highly complex intervention comprising multiple activities and involving many client groups with differing needs, it is likely that multiple mechanisms and interactions will be at work to bring about changes in individuals. At the core of the intervention is the contact with nature which has value in its own right, but also provides the platform for the range of activities. Studies have shown that contact with nature has a positive effect on people's mental, physical, and psychological well‐being, and spiritual beliefs (Bragg, 2013; Sempik, Hine, & Wilcox, 2010) and that engaging in nature based activities such as farming or gardening enables people to find solace (Sempik et al., 2010). As a result, care farms may be beneficial for a wide range of service users.

A number of theories have been mentioned within the care farming literature and some of these speak specifically to the nature element such as attention restoration theory (Kaplan & Kaplan, 1989) and biophilia hypothesis (Wilson, 1984). Other theories relate specifically to the client groups that attend care farms, for example, desistence theory for offenders (McNeill & Weaver, 2010) and the recovery model for people with mental health problems (Anthony, 1993). Within these theories, mechanisms are proposed to explain how any effective intervention would be expected to bring about change. Identifying these mechanisms within the care farming interventions will indicate its fit with the theory, and therefore its likely effectiveness. For example, desistence theory suggests that interventions that lead to a reduction in recidivism involve building human relationships, opportunities for reflection and change (Farrall & Bowling, 1999; Weaver & McNeill, 2007), developing self‐efficacy (Maruna, 2001; McCulloch, 2005; McNeill, 2006) and social capital by learning and applying new skills to develop a new more positive identity (Farrall, 2004; Giordano, Cernkovich, & Rudolph, 2002; Laub & Samson, 1993; Maruna, 2001; McNeill & Maruna, 2007). A sense of community and the development of friendships are indeed valued aspects of a CF (Hassink, 2009). Furthermore, farmers are perceived as a role model with a strong sense of identity, thus offering an essential role model that can be emulated within a new identity (Hassink, De Meyer, van der Sman, & Veerman, 2011). Both the concepts of building human relationships and creating a new identity are clearly present within care farming interventions.

### 3.4 Why it is important to do the review

With increasing pressures on the health and social care sector, commissioners and policy makers are turning to care farms as a potentially effective intervention. Farmers across Europe are becoming more multifunctional in how they use their land, and care farming may be an increasingly attractive option. As such, there is great potential to increase the use of care farms as an intervention to bring beneficial outcomes to a range of different population groups.

The growth in care farming in recent years is partly attributable to their commissioning successes with a range of health and social sector organisations through patient‐referral and contracts for provision of support to health, social‐care and probation clients. Their sustainability is important given the increasing reliance that health and social care place on them. However, they remain heavily dependent on charitable funding, and policy changes over recent years have detrimentally impacted income streams.

Care farming is one of many third sector health interventions that are competing for similar funding streams. Its strength is its clear capacity to deliver care to a wide range of service users. Their ethos fits well with a number of theories relating to, for example, mental health recovery and rehabilitation of offenders. As is common for many interventions delivered by the third sector, the evidence base for their effectiveness is not well developed. This undermines the ability of the sector to move beyond being peripheral support organisations with limited core funding. In the past, the need to provide evidence was the domain of the pharmaceutical industry, but in recent decades this has expanded to cover complex health service evaluations. The methodologies for the latter are transferable to the third sector, but a lack of infrastructure and sustained income has hindered the development of a robust evidence base here. Additionally, the complexities and multifaceted nature of care farms means that this is not an intervention that lends itself easily to a randomised controlled study design.

Nonetheless, there are a number of studies of care farms published in a wide range of journals across Europe. Although one systematic review and a small number of overviews exist (Bragg & Atkins, 2016; Elings, 2012b; Iancu et al., 2013), which document the extent and range of care farming initiatives and summarise the evidence for benefits, there is the need for a systematic review to capture the full range of both published and grey literature and to explore in depth the mechanisms that explain how care farms work for different client groups. Garnering this knowledge will help to clarify for policy makers and commissioners the unique contribution that care farming can make to health and social outcomes.

There is the potential for care farming to improve the health and well‐being of different population groups, and this is an important public health goal. If successful, they may have a role to play in reducing inequalities. Improving the lives of the most disadvantaged can have far‐reaching societal benefits, for example, through enhancing social cohesion, reducing use of health and social care service usage and reducing crime (Wilkinson & Pickett, 2009).

This review aims to synthesise the existing evidence on how and for whom, care farming works, in order to improve health and well‐being for a wide range of service users.

This systematic review is part of a feasibility and pilot study, funded by the National Institute of Health Research's Public Health Research Programme.

## 4 OBJECTIVES

The primary objective is to systematically review the available evidence of the effects of care farms on quality of life, health and social well‐being on service users.

Where possible we will synthesise the evidence in order:

1. To understand the size of the effect that care farms may have on the health, well‐being or social outcomes of different population groups
2. To examine whether effects differ depending on the activities and characteristics of the farm/farmer, the duration of time participants spend at the farm, the number and diversity of the participants on the farm, and whether the farm is the only intervention
3. To understand the mechanisms of change for different population groups attending care farms using a range of study methodologies, including qualitative studies

## 5 METHODS

### 5.1 General approach

We conducted a mixed methods synthesis using a sequential explanatory approach (Pluye & Hong, 2014) that involved the development of an intervention framework based on existing theories. These theories propose how care farming might work, and our review used qualitative and quantitative evidence to test the processes and outcomes suggested by these theories. This approach is valuable in identifying possible mechanisms of change to inform the development of a logic model for care farming. An earlier scoping review of the literature indicated a dearth of RCTs evaluating the effectiveness of care farms but instead highlighted a number of qualitative studies, a few small‐scale RCTs and observational studies. Thus a narrative approach which could synthesize the findings from a wide range of study designs was planned.

### 5.2 Criteria for considering studies for this review

#### 5.2.1 Types of studies

The study designs considered for inclusion in the review were:

- RCTs with randomisation at individual or cluster level.
- Quasi‐RCTs and cluster quasi‐RCTs, where participants are allocated by some means other than randomisation (e.g., by case number, date of birth).
- Interrupted time series that clearly define intervention points and record at least three outcome measurement points before and after (or before and during) the intervention.
- Nonrandomised observational studies that are prospective and have a control group, including:
  ○ Cohort studies, which ideally provide a reasonable timescale for effects to be detectable and attributable, and accurately record drop‐out figures/characteristics.
  ○ Case control studies that report cases and controls from studies where comparability on relevant baseline characteristics and potential confounders can be judged, and comprehensively report confounders.
  ○ Controlled before and after studies, where data collection must be contemporaneous and groups comparable on baseline scores.
- Before and after studies that do not have a control group:
  The findings provided useful information on the nature and context of care farms and the mechanisms that may support effectiveness.
- Qualitative studies:
  All designs of qualitative studies were considered, including phenomenology, ethnography, and grounded theory. In addition, we also included qualitative studies with different methods of analysis, such as thematic analysis discourse/conversation analysis and narrative analysis.

We excluded single subject designs, reviews, overviews, surveys, commentaries and editorials. We also excluded theses with empirical data that had been subsequently published elsewhere.

In addition to these study designs, we also retained papers which described any theories offering explanations for how care farms might bring about change in the various population groups under investigation. As our interest here is purely to explore the theoretical basis by which care farming might work to initially inform the logic model(s), we do not refer to these papers as “included studies” or “excluded studies”. These latter terms are for empirical data.

#### 5.2.2 Types of participants

Service users attending care farms of any age were included in the review. The list below presents the likely population groups:

- Offenders serving community orders or similar sentences in the community rather than in prison; offenders “on‐licence” (i.e., recently leaving prison to re‐enter the community)
- People with substance misuse, such as drug and alcohol problems
- People with mental health problems, including anxiety, depression and psychiatric disorders
- Young people with challenging behaviour, particularly those excluded/facing exclusion from school or those at risk of offending
- People with health problems, particularly long‐term conditions, including dementia
- People with learning difficulties
- People receiving palliative care
- Socially isolated older people

We excluded studies if participants were not from a vulnerable or disadvantaged population, such as school children visiting for education purposes or adults visiting as conservation volunteers.

#### 5.2.3 Types of interventions

All care farms have some degree of “farming” (crops, livestock, woodland, etc.) and of “care” (including health care, social rehabilitation, education or training), but the balance of these elements differs from CF to care farm.

We included studies where the intervention met the definition of care farming (see Section 3.2.1 for definition). The definition of care farming includes a number of components, each of which requires clarification to define exactly what was included and excluded in the review. These components include:

(a) “Providing a supervised, structured programme of farming‐related activities”: many care farms offer contact with farm livestock or with crops and plants. Studies were included in the review if a range of farming activities were delivered. We excluded studies with a single activity such as gardening or horse riding. This provides a clear distinction between the variety of social and therapeutic horticultural activities, AAI and care farming. We excluded interventions that are purely categorised as “therapy”, whether in relation to animals or other natural elements that are not part of a working farm; examples include pet therapy and donkey/equine therapy.

(b) “Providing services on a regular basis for participants”: studies were included if the intervention was structured and service users attended several sessions rather than a planned “one‐off” visit. The review also excluded petting farms and farms used for “one‐off” educational activities.

We excluded care farming interventions that were combined with other interventions (i.e., music therapy) as we would be unable to differentiate the effects derived from actual farm work. We also excluded farming interventions that were provided in hospital or in prisons.

Eligible comparators included no intervention, wait‐list controls or alternative interventions. Comparators were specific to the population group studied, for example, offenders serving their community order on a CF were compared to those serving their order cleaning public areas; or for those with addiction problems, another drug rehabilitation programme.

#### 5.2.4 Types of outcome measures

##### Primary outcomes

Care farms aim to improve a complex collection of social, educational and health outcomes for their service users. Given that the possible end impact of this complex interaction will be seen in changes in quality of life and mental health, this review included quality of life, anxiety and depression as the primary outcomes. Studies that did not use a validated instrument were not included in the analysis.

##### Secondary outcomes

Secondary outcomes varied according to the different populations, but we reported any mental health outcomes (in addition to quality of life, depression and anxiety as primary outcomes), social, physical and behavioural outcomes. Although we report all relevant outcomes we do not include in the analysis any secondary outcomes that had been developed in‐house or failed to be defined.

The secondary outcomes included were:

- Mental health outcomes: self‐efficacy, self‐esteem, stress, coping, mood, mental status, mental functioning, positive affect, rehabilitation and cognitive functioning, empowerment
- Social outcomes: social functioning/interaction, group cohesion, recidivism, employment, school exclusion
- Physical outcomes: functional performance, physical activity, and appetite and eating pattern
- Behavioural outcomes: drug use, alcohol intake and smoking

#### 5.2.5 Duration of follow‐up

The review included any length of follow‐up of participants after their attendance at the care farm. Studies that only collected follow‐up data at the beginning and at the end of each day were excluded.

#### 5.2.6 Types of settings

To be included, the studies need to explicitly state that activities took place on a farm that was not part of an institutional setting such as a prison or hospital. Community gardens and allotments were excluded.

### 5.3 Search methods for identification of studies

#### 5.3.1 Electronic searches

Health, education, environmental, criminal justice and social science databases were searched to identify studies from a variety of disciplines. Care farms are seen as both a health and a social intervention, and so are likely to be reported in the literature relating to these disciplines. The selection of databases is extensive, offering a good international coverage of journals in attempt to identify relevant studies throughout the world. Further databases were added to those already identified in the protocol in order to identify studies commensurate with the range of potential outcomes and population groups. A single search strategy was used to identify both quantitative and qualitative studies. No restrictions were imposed on publication format or language in the search strategy.

In November 2014 we searched the following databases:

- Applied Social Sciences Index and Abstracts (ASSIA) (ProQuest) from 1987
- CINAHL (EBSCO) from 1981
- The Campbell Library
- Criminal Justice Abstracts (EBSCO) from 1830
- Conference Proceedings Citation Index‐Science (Thomson Reuters Web of Science) from 1990
- Conference Proceedings Citation Index‐Social Science and Humanities (Thomson Reuters Web of Science) from 1990
- Embase Classic + Embase (Ovid) from 1947
- ERIC (ProQuest) from 1966
- FRANCIS (EBSCO) from 1972
- Global Health (Ovid) from 1910
- GreenFILE (EBSCO) from 1910
- Ovid MEDLINE(R) from 1946
- Ovid MEDLINE(R) In‐Process and Other Non‐Indexed Citations
- National Criminal Justice Reference Service Abstracts (ProQuest) from 1975
- PsycINFO (Ovid) from 1806
- Sciences Citation Index (Thomson Reuters Web of Science) from 1900
- Scopus (Elsevier B.V.) from 1823
- Social Care Online (SCIE) from 1980
- Social Sciences Citation Index (Thomson Reuters Web of Science) from 1900
- Social Services Abstracts (ProQuest) from 1979
- Sociological Abstracts (ProQuest) from 1925
- Web of Science. Science Citation Index Expanded (SCI‐EXPANDED), Social Sciences Citation Index (SSCI) from 1900

#### 5.3.2 Searching other resources

In order to further limit publication bias and improve the generalisability of results, we searched databases of grey literature (including conferences, dissertations and reports) and websites likely to contain unpublished reports of studies on care farms. In November 2014, we searched care farming websites in English, Dutch, Spanish and Italian to identify grey literature.

Resources searched:

- HMIC Health Management and Information Consortium 1983+ (Ovid)
- ProQuest Dissertations and Theses A&I 1743+ (Proquest)
- Web of Science. Conference Proceedings Citation Index‐Science (CPCI‐S) 1990–present (Thomson Reuters)
- Web of Science. Conference Proceedings Citation Index‐Social Science and Humanities (CPCI‐SSH) 1990–present (Thomson Reuters)
- Databases of ongoing trials such as Current Controlled Trials (http://www.controlled‐trials.com/).
- Websites also searched in November 2014:
  ○ European Network for Rural Development http://enrd.ec.europa.eu/
  ○ Ministry of Justice https://www.justice.gov.uk/
  ○ Care Farming UK http://www.carefarminguk.org/
  ○ Social farming in Europe http://sofar.unipi.it/index_file/socialfarfming.htm
  ○ http://www.umb.no/greencare
  ○ http://library.wur.nl/frontis/farming_for_health
  ○ http://www.greenchimneys.org
  ○ https://www.novapublishers.com/catalog/product_info.php?products_id=41368
  ○ http://www.regjeringen.no/nb/dep/lmd/dok/rapporter‐og‐planer/rapporter/2012/green‐care‐in‐the‐nordic‐countries‐‐a‐re.html?id=712600 (Nordic report)
  ○ http://www.matmerk.no/inn‐pa‐tunet (Norwegian website for green care)

In addition to electronic and unpublished literature searches, we identified further relevant studies by examining the reference lists of included studies and any relevant systematic reviews identified, and by tracking citations of key papers using Google Scholar and Web of Science Citation Indexes. We used social/care farming and other relevant academic networks across Europe to contact research experts or farmers to request any evaluations they have conducted. Further relevant studies were identified through citation tracking activities. We hand‐searched the NJAS‐*Wageningen Journal of Life Sciences* (2000–2015) and the abstracts of Care Farm and Social Farm conferences held in the UK and Europe (2005–2015).

#### 5.3.3 Search terms

The searches identified studies of care farms or agricultural‐related therapies and rehabilitation practices within a farm setting. The searches were not limited to a particular study type or participant group. Scoping searches have indicated limited literature on “care farms”, and we therefore supplemented the “care farm” phrase searches with a broader search to identify agricultural‐related therapeutic and rehabilitative interventions that occur in farm settings. Our search strategy excluded references indexed as animal‐only studies due to the high number of veterinary science studies of therapies for farm animals.

The searches were comprised of a number of components and search terms using subject headings and text words, truncation, and phrase searching where appropriate (Appendix 12.1). Alerting systems were set up in databases (where available) to keep the reviewers aware of any studies published during the time frame of the review. The full search strategy can be found in Appendix 12.2.

### 5.4 Data collection and analysis

#### 5.4.1 Selection of studies

We used a two stage screening process to identify eligible studies.

- Screening 1: Titles and abstracts
  Two reviewers independently screened the titles and abstracts of articles and grey literature retrieved to assess eligibility, as determined by the inclusion and exclusion criteria listed above.
- Screening 2: Full text
  For those studies that were selected as potentially eligible for inclusion, full copies were retrieved and two reviewers independently assessed whether studies met the inclusion criteria.
  Any disagreements were discussed and resolved by consensus at each stage of the eligibility assessment. Multiple reports from the same study were coded separately before combining information across reports. We used the PRISMA chart to detail the process of study selection (Moher, Liberati, Tetzalaff, & Altman, 2009).
- Additional screening 3: Theories mentioned in care farming publications
  During full paper screening we also looked for theories that had been applied or mentioned within care farming studies. Even if the paper did not meet all of the inclusion criteria, it was retained so that we could use this as a source for identifying relevant theory. The aim was to collate all theories quoted in relation to care farming, which were then explored in greater detail and used as a basis for our theoretical framework that explores the mechanisms of the intervention.

#### 5.4.2 Data extraction and management

We stored all the references identified by the search in EndNote software (Version X7). We recorded (in a Microsoft Excel spreadsheet) all websites searched and the details of any reports found or further contacts made. When screening full papers, we used the EPPI‐Reviewer 4 (V.4.5.0.1) software to keep records of all eligibility decisions.

Information on study design, sample characteristics, intervention characteristics (including contextual information about the care farms) and outcomes were extracted from studies using an adapted version of the CPHG Data Extraction and Assessment Template (Higgins & Green, 2011) (see Appendix 13.3). We used separate data extraction forms for recording contextual data about care farming interventions (see Appendix 13.4), data from qualitative studies (see Appendix 13.5) and data from quantitative studies (see Appendix 13.6). Primary investigators were contacted to request information on missing data.

The data extraction for included information on the unit of analysis used in the studies, particularly where individual or cluster randomisation had occurred and whether individuals had received multiple interventions.

During data extraction, we only included qualitative themes that represented the views of the CF service users. However, in studies involving service users with communication difficulties, we included themes based on the recorded perspectives of significant others (care farmers, carers and parents) on the impact of the CF on the service users. We excluded themes from others that were about their own experiences, for example, care farmers’ views on running a farm.

For papers that reported theories related to care farming, we extracted any summaries explaining how care farms might work and the anticipated outcomes. If the identified paper failed to provide an adequate description of this process we sought to identify the seminal paper.

#### 5.4.3 Assessment of risk of bias in included studies

Qualitative studies were assessed using an adapted version of the consolidated criteria for reporting qualitative research (COREQ) tool (Long & Godfrey, 2004; Tong, Sainsbury, & Craig, 2007) (see Appendix 13.7). Three categories of reporting standards were established for each item: clearly met, unsure and not met. We did not exclude any qualitative studies based on bias.

The EPOC Risk of Bias tool was used to appraise RCTs (Higgins & Green, 2011) (see Appendix 13.8). The Effective Public Health Practice Project tool (EPHPP) was used to appraise other quantitative studies, such as controlled before and after studies or uncontrolled before and after studies (Armijo‐Olivo, Stiles, Hagen, Biondo, & Cummings, 2012) (Appendix 13.8). Studies with the majority of domains categorised as “unclear” in the EPOC Risk of Bias tool were coded as high risk of bias; similarly, studies with two domains categorised as “weak” in the EPHPP tool were coded as high risk of bias. We did not exclude any studies based on risk of bias.

We pilot tested the tools with a sub‐set of identified studies to ensure a consistent approach to assessment within the team. Two reviewers independently assessed risk of bias for each study. We resolved any disagreement by discussion or by involving an additional review team member.

#### 5.4.4 Measures of treatment effect

Where sufficient data was available we calculated effect sizes and 95% confidence intervals for each study using the Campbell Collaboration Effect Size Calculator for RCT's and CBA studies (Wilson, n.d. https://campbellcollaboration.org/escalc/html/EffectSizeCalculator‐SMD1.php). Where data, such as standard deviations or pre/post correlation data was unavailable, we contacted study authors. For studies without a control group and those where sufficient data was not available to calculate an effect size and study authors did not respond to requests for data, these studies have been included in the review and we report study authors’ findings. If meta‐analysis had been possible, these studies would not be eligible for inclusion in meta‐analysis.

There were not a sufficient number of studies that reported enough data to calculate effect sizes in any outcome category, thus quantitative studies were not quantitatively synthesised. The results of the quantitative studies are provided in Table 14 (Outcomes) and Table 15 (Proximal Outcomes) which present the effects of individual studies.

#### 5.4.5 Data synthesis of qualitative and quantitative findings

**Figure 2:**
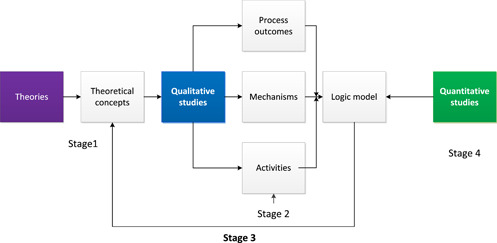
Process of data synthesis

We based our data synthesis on a sequential exploratory approach (Pluye & Hong, 2014) (see Figure 2). This method involves: (a) identifying the main concepts from within theories found in relevant literature to explain why the intervention may work, (b) synthesising the qualitative data and then (c) interrogating the quantitative data to test the qualitative findings. There were several stages within this synthesis which ultimately aided the construction of a logic model to explain how care farms might work for the heterogeneous study population as a whole and also for each population group. We based the design of our logic models on the description and definitions provided by the MRC's guidance for process evaluation of complex interventions (Moore et al., 2011). Here, a logic model is defined as:

*A diagrammatic representation of an intervention, describing anticipated delivery mechanisms (e.g. how resources will be applied to ensure implementation), intervention components (what is to be implemented), mechanisms of impact (the mechanisms through which an intervention will work) and intended outcomes*. Reproduced from Moore et al. (2011) (p. 8).

Given the range of outcomes studied in care farms research, we designed our logic models to distinguish between “endpoint” health outcomes and proximal outcomes or mediators which are likely to be on the path to the endpoint health outcomes.

The stages of the synthesis were as follows:

- Stage 1: Development of a preliminary theoretical framework to explain potential mechanisms of change
- Stage 2: Identification of the key mechanisms of change, activities or intervention components and proximal outcomes as reported by service users attending care farms within qualitative studies
- Stage 3: Mapping of the qualitative mechanisms and proximal outcomes to the theoretical concepts to create a logic model
- Stage 4: Mapping of the outcomes from the quantitative studies to the logic model to identify out where the evidence lay

##### Stage 1

In Stage 1, we examined papers that reported theories relating to care farming. If the included study reports did not provide adequate description of the theories, we retrieved the seminal articles of interventions which further described the theories. Two reviewers (J. M. and N. W.) extracted information on the issue being addressed and the proposed mechanism of change. Each summary was compared to identify areas of overlap in order to create a condensed set of concepts upon which to map the evidence. One reviewer (J. M.) conducted the analysis, and this was subsequently checked by a second reviewer (N. W.).

##### Stage 2

In Stage 2, two reviewers (N. W. and J. M. or H. E.) extracted themes from the qualitative studies to ensure that all relevant data had been captured. Where discrete themes were not presented in the papers, we looked for evidence of consensus among the participants as well as any discordant experiences. Negative as well as positive experiences were extracted. We opted for an inclusive approach to data extraction in the absence of discrete themes.

The same reviewers independently reviewed each of the extracted themes to identify which were composite and represented multiple discrete findings. These composite themes were independently deconstructed and the eventual findings were compared to ensure consensus on interpretation.

Each finding was entered into a spreadsheet, alongside its source and the client group studied. Three reviewers (J. M., N. W. and H. E.) independently categorised each finding as an intervention component (activity), mechanism, proximal and health outcome. These preidentified categories followed the definitions described in the MRC's 2011 model (Moore et al., 2011). Each finding was defined as:

- Intervention: These included the facilities, activities and structure provided as part of the farm.
- Mechanism: The process by which part of the intervention might result in a particular outcome. These tended to be subjective experiences such as feelings and perceptions. For example, having physical contact with the animals (the intervention) would provide a sense of warmth and calmness (mechanism). Explicit links between mechanism and part of the intervention were not always reported. There can be multiple and linear mechanisms leading to the same outcome.
- Proximal outcome: An immediate outcome derived from a particular mechanism within the intervention. Primary health outcomes, as previously defined in this review, would not be categorised as a proximal outcome here. For example, having time away (mechanism) would lead to a sense of calm and reflection (also a mechanism) and feeling reduced stress (a proximal outcome). The key here is that there can be multiple proximal outcomes which mediate between the intervention activity and the outcome.

On agreement between the reviewers, each finding was transcribed onto a Post‐It note in preparation for a clustering exercise (Backoff & Nutt, 1988).

The clustering exercise involved six additional reviewers (R. B., M. E., C. B., J. C., S. T. and D. S.), first, checking the groupings of intervention components, mechanisms and proximal outcomes on the Post‐It notes. Areas of disagreement were reviewed and amendments made if required. The Post‐It notes with recorded mechanisms were divided up among the six reviewers who were asked to place one note each on a blank wall. The reviewers were then asked to continue placing their Post‐It notes on areas of the wall according to emerging clusters of similar mechanisms. The exercise continued in silence until all the Post‐It notes had been allocated. Reviewers were then asked to stand back and review the clusters of Post‐Its on the wall and were given permission to move notes around without explanation. Once completed the reviewers then discussed the composition and meaning of each cluster. Each cluster (now assigned category) was labelled and entered onto the spreadsheet. Detailed additional analysis of the contents of each category was performed by three reviewers (J. M., N. W. and H. E.) to ensure that each of the findings had not been over‐interpreted (i.e., assumptions about what the mechanism might lead to) and thus placed in an unsuitable category. Given that the findings had been decontextualised during extraction and deconstruction of themes, this was an important iterative step that enabled the data to remain true to its source.

For the intervention components, one reviewer (J. M.) grouped the findings according to congruency and labelled each of the categories; this was subsequently checked by another reviewer (N. W.).

As a gauge of the potential relative importance of each of the categories of mechanisms, we assessed the spread of the categories (across all the studies) and the frequency of the findings within each category. We carried this out for all the studies (all population groups) and for each individual population group (wherever possible). We ordered the categories based on this assessment to explore the possibility that care farms might work in different ways for different populations.

##### Stage 3

The categories of, interventions, mechanisms and proximal outcomes were mapped to the theoretical concepts identified in Stage 1. This was performed by one reviewer (J. M.) and checked by a further two reviewers (N. W. and H. E.). The aim was to understand the ways in which change occurred and start testing out the theories using empirical data.

##### Stage 4

Two reviewers (N. W. and J. M. or H. E.) independently extracted all the quantitative results reported in the included studies. The quantitative data were summarised narratively according to the ESRC guidance (Popay, 2006). First, we assessed whether the care farms improved service user outcomes, caused harm to the service users or had no effect. If significant positive findings were observed, we searched for clinical cut‐offs to determine if the positive finding was clinically meaningful. Second, we presented results as they were presented in the original studies for our primary outcomes. For example, if three studies measured quality of life, we reported each study finding separately showing whether they had found positive or negative results. Due to the considerable difference between studies, in terms of population groups studied, outcome measures and study designs, the results of the quantitative studies were not combined. They have instead been presented as individual study results against each outcome category. When sufficient data were available, effect sizes were calculated using the Campbell Collaboration effect size calculation tool. Third, we evaluated the strength of the evidence using the findings from the risk of bias assessment. For example, if a study reported a positive finding, we then checked to see if that study was free from systematic error. Finally, we mapped these quantitative results separately for each study to the logic model. This helped to identify outcomes in our logic model supported by the evidence base.

#### 5.4.6 Sensitivity analysis

To measure the robustness of the results we planned to conduct sensitivity analyses. We intended to conduct sensitivity analyses according to study design (i.e., excluding controlled before and after designs and any other non‐RCTs) and according to the risk of bias, whereby we would assess sensitivity based on the inclusion and exclusion of studies with high risk of bias.

#### 5.4.7 Assessment of publication biases

We planned to use funnel plots for information about possible publication bias if we find sufficient studies (Higgins & Green, 2011). A minimum of 10 studies with a common outcome measure is needed to be able to distinguish chance from real asymmetry (i.e., true publication bias) within the funnel plots (Higgins & Green, 2011). If asymmetry was found to be present, we would consider possible reasons for this.

#### 5.4.8 Deviations from protocol

In addition to providing a summary of risk of bias across the various domains within the studies, we had planned to summarise the overall weight of evidence that each study would contribute the review findings. However, recent Campbell reviews have tended not to use an overall quality scale. This is based on the concern that assessments of overall risk of bias may not take into consideration specific domains and are too dependent on the type of quality scale used (Brody et al., 2015).

Following the search and data extraction process, it became clear that there were several additional population groups using care farms which we had not been identified when writing the protocol. Given that our review aimed to understand how care farms may “work” for disadvantaged groups we decided to include any group that could be considered disadvantaged in some way. In light of this we included “socially isolated older people” but added an exclusion for “participants not from a vulnerable or disadvantaged population”.

The process of review and data extraction helped us to further reflect on the definition of care farms. The definition of a CF used within the protocol was: “use of commercial farms and agricultural landscapes as a base for promoting mental and physical health through normal farming activities. Specifically, providing a structured supervised programme of health, vocational, social and/or farm related activities for vulnerable people.”

Within the review process, the importance of the “normal farming activities” became clearer and helped us to distinguish between interventions that were specifically designed as a “therapy”, for example, horticultural therapy or equine therapy, and care farming which primarily focused on farming activity to sustain the farm and production, rather than primarily as therapy.

The review process identified a diverse range of primary and proximal outcomes. The protocol stated that the primary outcome was “quality of life”. However, the review process identified a large number of studies (nine were included) that measured depression and anxiety. As these outcomes are frequently considered as “endpoint” health outcomes, we included these as primary outcomes in our presentation of results and the logic models.

The proximal and secondary outcomes identified during the review were varied and numerous. As described in the protocol we included any outcomes that used a recognised measure of health or wellbeing or behavioural factor and were assessed using self‐report or objective measures. This helped us to identify pathways to change for different disadvantaged groups and develop a logic model to explain these relationships. Being too restrictive in the secondary outcomes for our review would have limited our understanding of these potential mechanisms.

In addition to the extraction fields specified in the protocol, we also extracted data on “duration of follow‐up” (5.2.5) and “types of settings” (5.2.6). This enabled us to understand the importance of the setting and the sustainability of the impacts of cares farms on participant outcomes.

The protocol included a broad outline of the qualitative synthesis process. The detailed process of qualitative analysis using the four steps described in this report developed following further training of the review team on mixed methods systematic reviews.

## 6 RESULTS

### 6.1 Description of studies

#### 6.1.1 Results of the search

**Figure 3:**
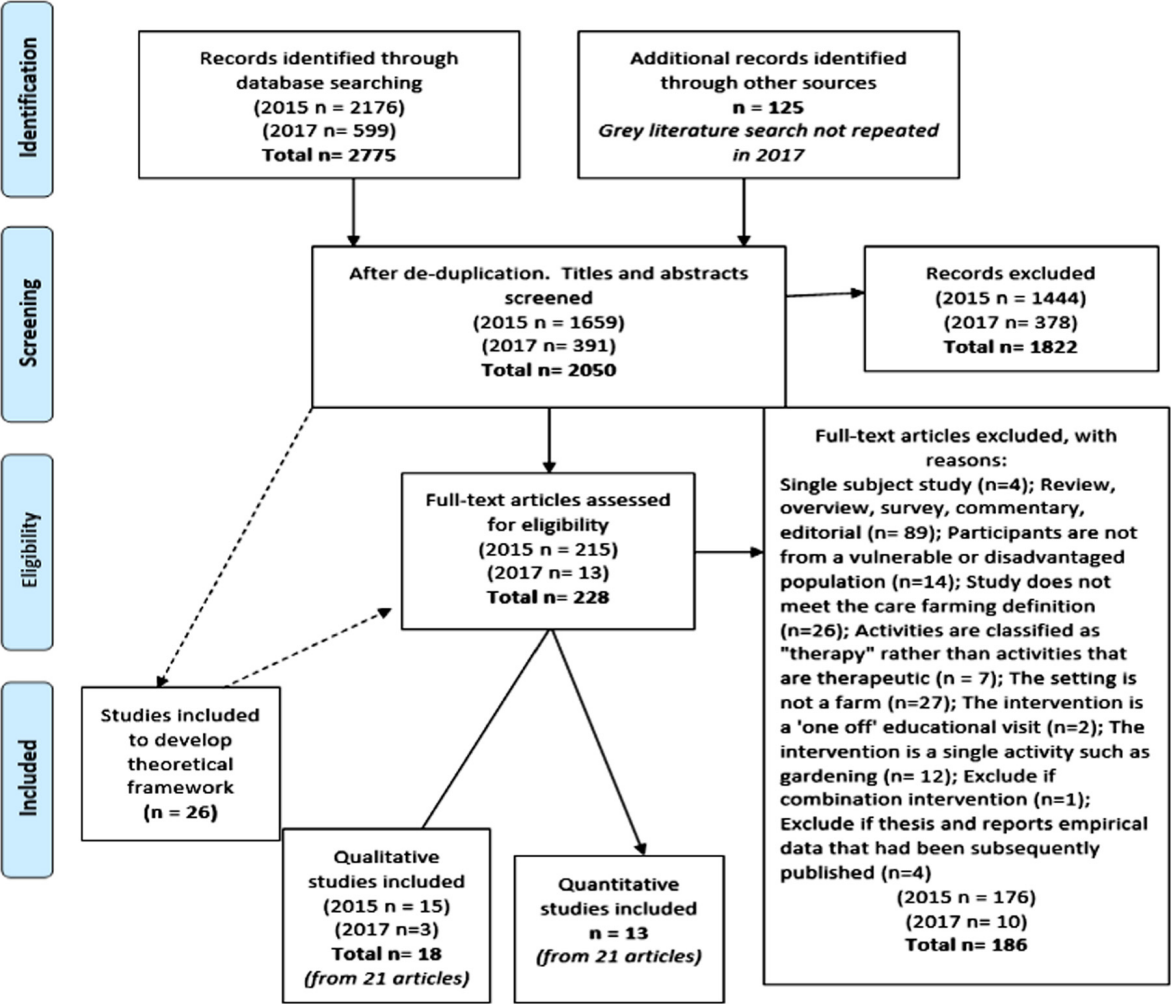
PRISMA diagram

We found 2,176 articles through searching of electronic databases and 125 via grey literature retrieval methods (see Figure 3). After duplicates were removed, we screened 1,659 references based on title and abstract. We obtained full copies of 215 articles and, of these, 31 studies (reported in 42 papers) met the inclusion criteria.

**Figure 4:**
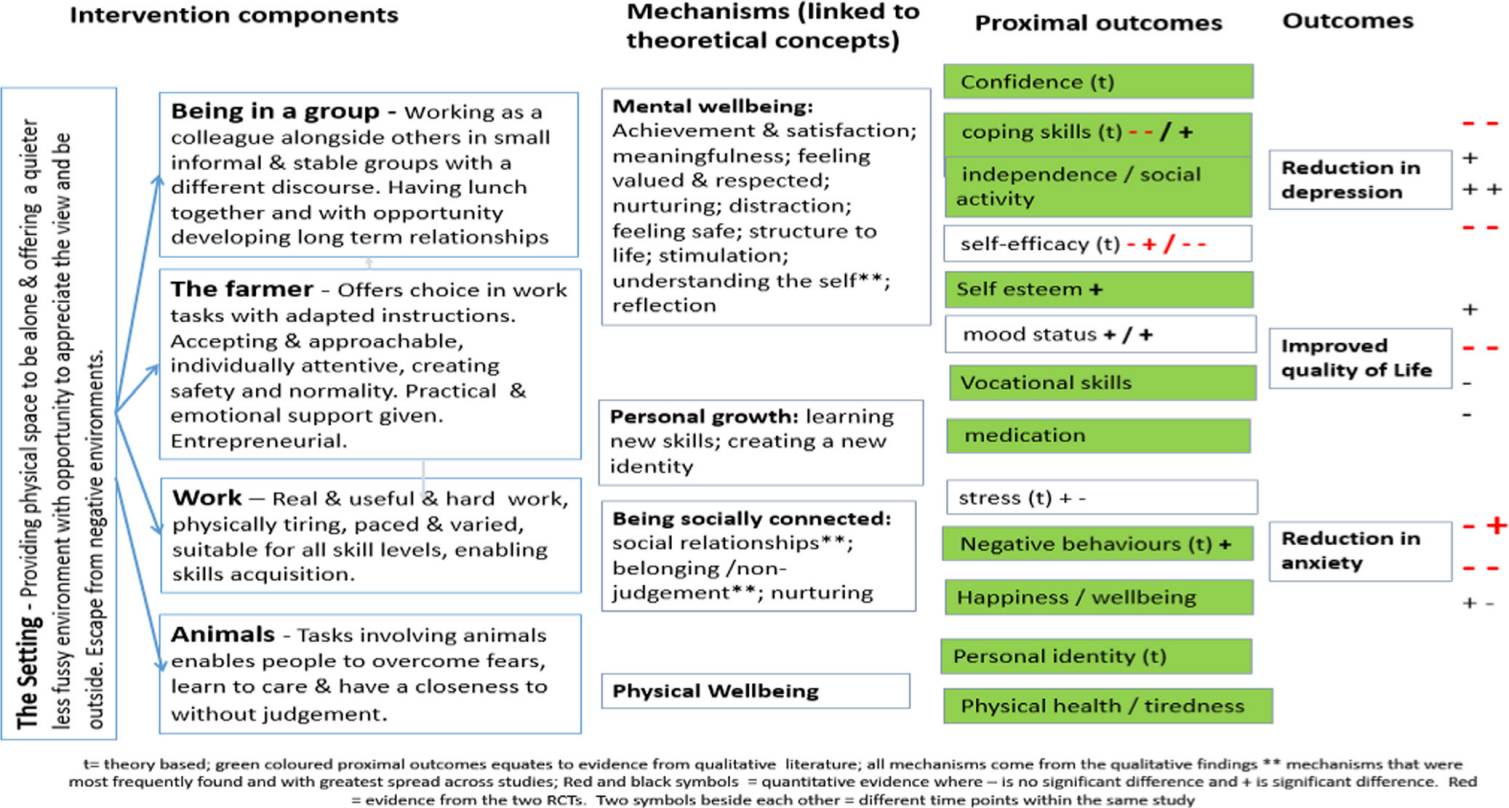
Logic model for all client groups

**Figure 5:**
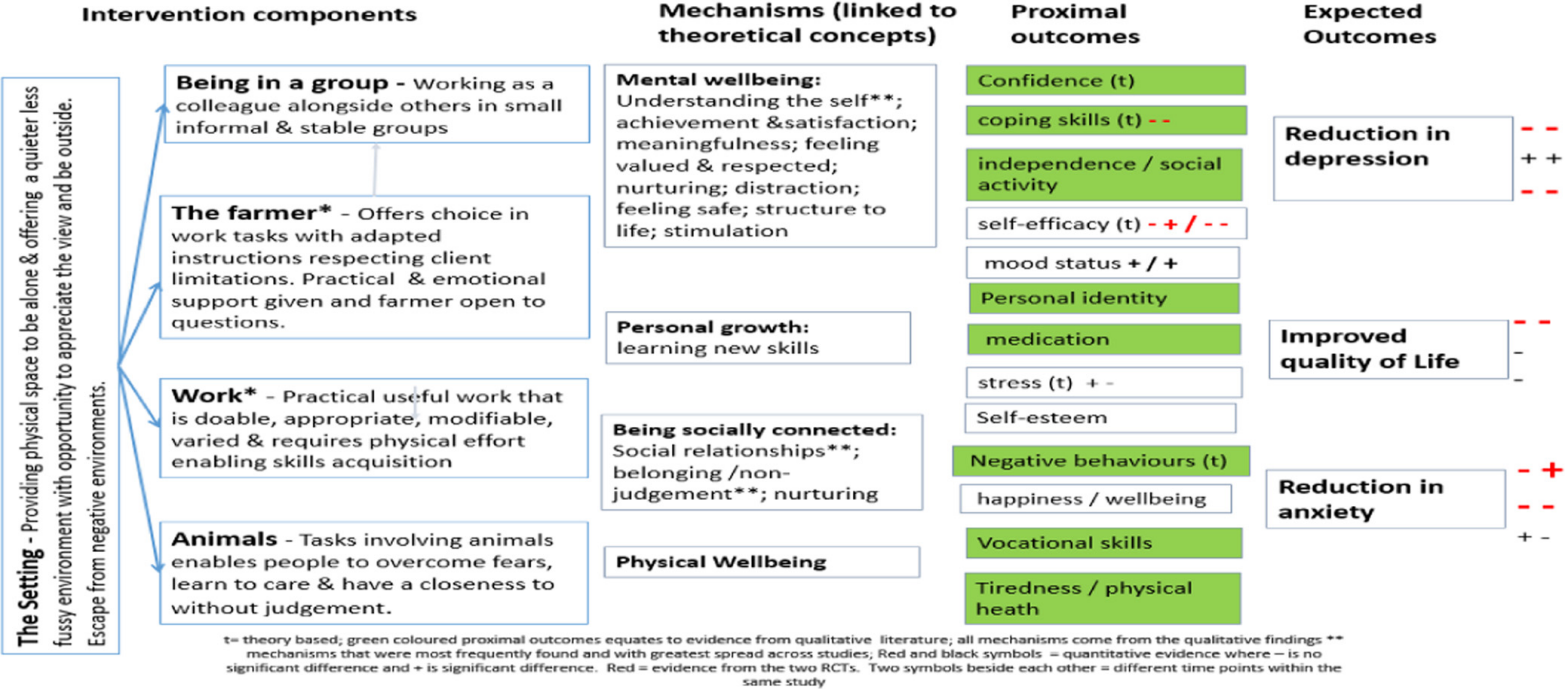
Logic model for mental ill‐health/substance misuse client groups

**Figure 6:**
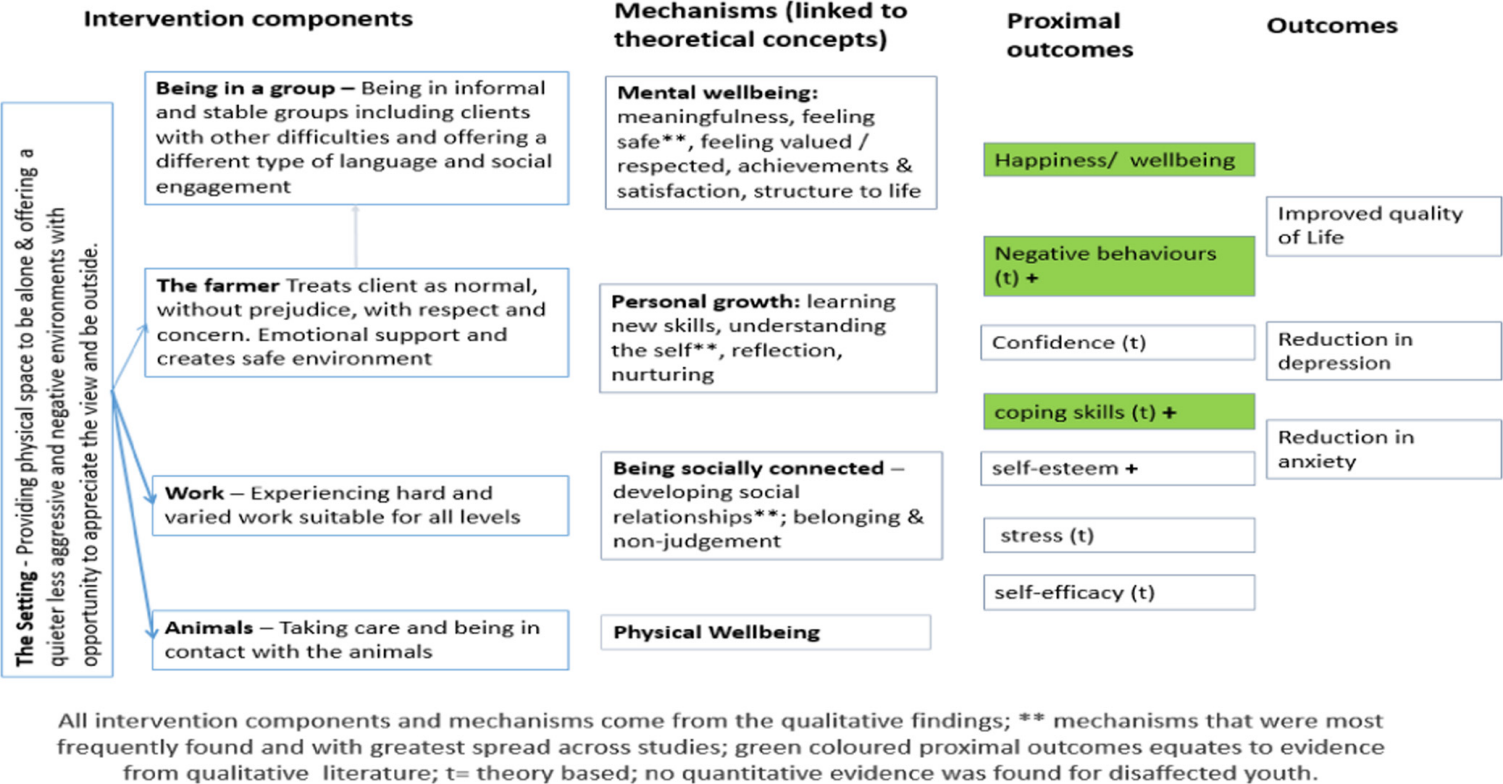
Logic model for disaffected youth group

**Figure 7:**
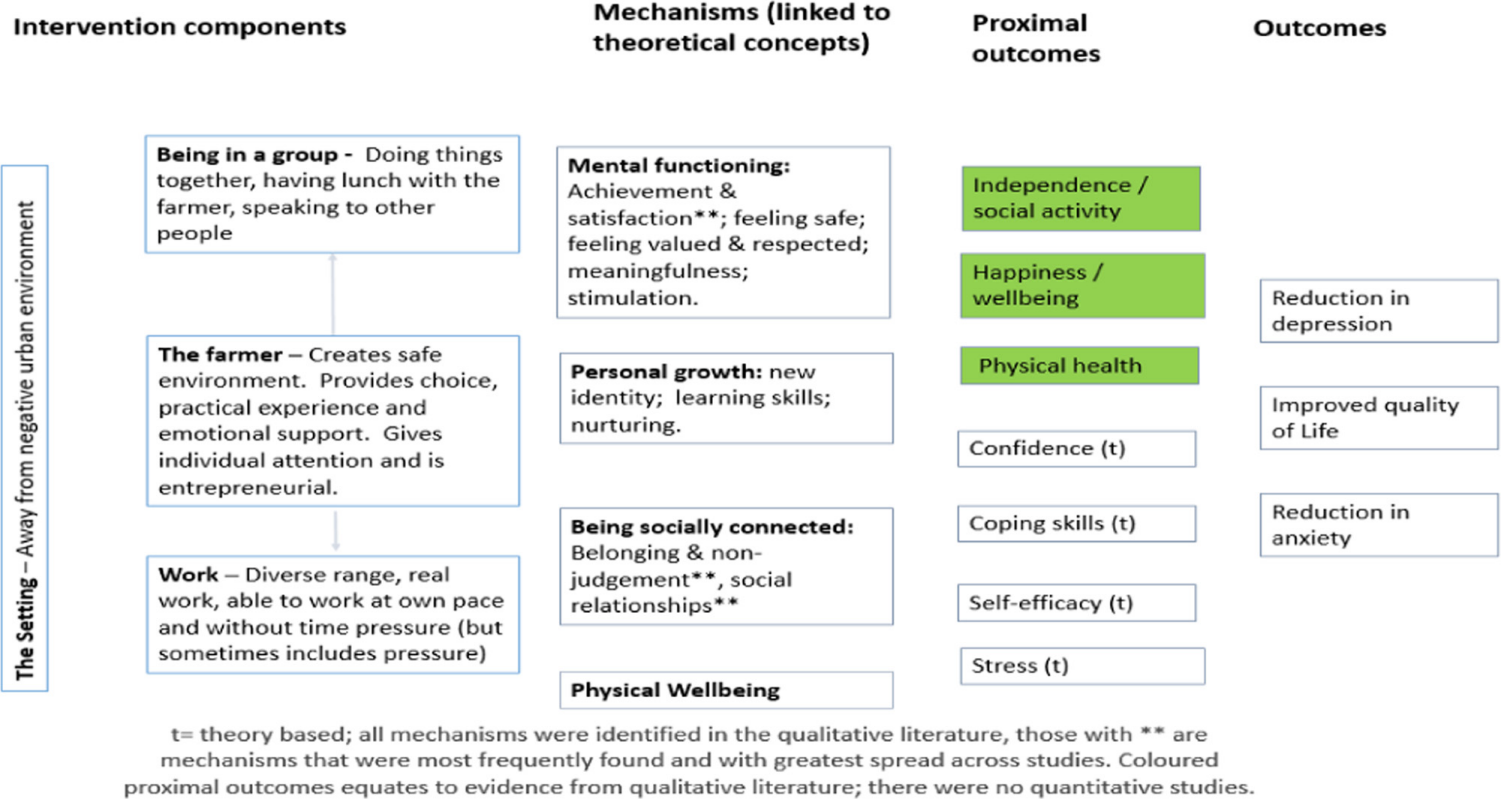
Logic model for learning disabilities client group

In a separate screening process, we were able to identify theoretical and contextual information about care farming interventions in 26 publications. Seven of these theory publications also reported empirical work, six had used qualitative methods and one was an uncontrolled before and after study. These seven studies were screened and included in the subsequent stages of the review, that is, in the 31 studies mentioned above. Those that were purely theoretical or did not meet our inclusion criteria for empirical studies, were used only for Stage 1 of the review process.

#### 6.1.2 Included studies

A total of 31 studies were included. Eighteen qualitative studies (reported in 21 papers) (Table 1), 13 quantitative studies (reported in 21 papers) (Table 2), and one mixed methods study (Elings et al., 2011) met the inclusion criteria for this review.

**Table 1:** Characteristics of qualitative studies

References	Country	Client group	Method	Numbers Of interviewees	Age, mean (range)	Gender	Quality criteria met
Baars et al. (2009)	The Netherlands	Mental ill‐health	Interviews and photography	8	39	4 male, 4 female	<50%
Bjørgen and Johansen (2007)	Norway	Mental ill‐health	Focus groups	15^a^	–	–	<50%
Elings (2004)	The Netherlands	Learning disabilities	Interviews, participatory observation	18^a^	–	–	<50%
Elings and Beerens (2012); Elings and Hassink (2008, 2010)	The Netherlands	Mental ill‐health: psychiatric; substance misuse	Focus groups	42	–	–	<50%
Elings et al. (2011)	The Netherlands	Mental ill‐health; substance misuse	Interviews and focus groups	55^a^	–	–	<50%
Ferwerda‐van Zonneveld et al. (2012)	The Netherlands	Children with autism spectrum disorders	Interviews	7^b^	–	1 male, 6 female	<50%
Granerud and Eriksson (2014)	Norway	Mental ill‐health: long‐standing severe psychotic disorders, personality disorders; substance misuse	Interviews	20	22–55	8 male, 12 female	>50%
Hassink, 2009; Hassink, Elings, Zweekhorst, van den Nieuwenhuizen, and Smit (2010)	The Netherlands	Mental ill‐health; disaffected/excluded youth; older people	Interviews	41^a^	–	30 male, 11 female	>50%
Iancu et al. (2014)	The Netherlands	Mental ill‐health	Interviews	26	–	16 male, 10 female	>50%
Kaley (2015)	UK	Learning difficulties	Interviews and video recording, and photographic method	7^a^	–	7 male, 0 female	>50%
Kogstad et al. (2014)	Norway	Disaffected/excluded youth	Interviews	9	22.5 (17–27)	2 male, 7 female	>50%
Leck et al. (2015)	UK	Mental ill‐health; substance misuse; disaffected/excluded youth; learning difficulties	Interviews and focus groups	33	–	26 male, 7 female	<50%
Pedersen et al. (2012a)	Norway	Mental ill‐health: people with depression	Interviews	8	37.6 (27–54)	1 male, 7 female	>50%
Schreuder et al. (2014)	The Netherlands	Disaffected/excluded youth	Interviews	11	(16–23)	9 male, 2 female	>50%
North Essex Research Network & South Essex Service User Research Group (2013)	UK	Mental ill‐health	Interviews	5	–	4 male, 1 female	<50%
De Bruin et al. (2015)	The Netherlands	Dementia	Interviews	21^a^	71 (±7.5)	18 male, 3 female	>50%
Anderson, Chapin, Reimer, and Siffri (2017)	USA	Mental ill‐health; cognitive impairment	Interviews	5^a^	37–87	–	<50
Ellingsen‐Dalskau et al. (2016)	Norway	Mental‐ill health	Interviews	10	20–42	2 male. 8 female	>50

^a^ Others also interviewed in the studies: Bjørgen and Johansen (2007), four officials from contracting agency; Elings (2004), 16 carers/farmers (most of the interviewing was conducted with farmers); Ferwerda‐van Zonneveld et al. (2012), seven farmers; Hassink (2009) and Hassink et al. (2010), 33 farmers and 27 health professionals; Kaley (2015), six car farm staff and seven carers (this data only supplemented the interviews with service users; de Bruin et al. (2015), 12 people on a waiting list for the CF and 17 people attending regular day care services.

^b^ These were farmers who provided information on behalf of the service users.

**Table 2:** Characteristics of quantitative studies

Study design	References	Country	Client group	Control group	Sample size	Age	Gender	Duration	Proximal outcomes (measures)	Outcomes (measures)	Follow‐ups
RCT	Berget et al. (2008, 2011, 2007)	Norway	Mental ill‐health: patients with psychiatric disorders	Treatment as usual	90 for Qol and 69 for BDI and STAI	34.7 ± 10.7 (18–58)	31 (34.4%) male, 59 (65.6%) female	3 hr, twice a week, for 12 weeks	Coping (Coping Strategies Scale); self efficacy (Generalised Self‐Efficacy Scale); work abilities^a^, ^b^	Quality of life (Norwegian version of Quality of Life Scale); depression (The Beck Depression Inventory); anxiety (The Spielberger State‐Trait Anxiety Inventory)	12 weeks (immediately after CF) and 6 months
RCT	Pedersen et al. (2012b)	Norway	Mental ill‐health: people with clinical depression	Wait list control group	29	Intervention: 40.5 ± 10.7; control: 34.0 ± 6.6	Intervention: 5 male, 11 female; control: 1 male, 12 female	1.5–3 hr, twice a week, for 12 weeks	Self‐efficacy (Generalised Self‐Efficacy Scale)	Depression (Beck Depression Inventory); anxiety (The Spielberger State‐Trait Anxiety Inventory‐State Subscale)	12 weeks and 3 months after the intervention
CBA	de Bruin (2009); de Bruin et al. (2012)	The Netherlands	Older people >65 with dementia	Regular day care facilities	88	Intervention: 77.6 ± 6; control: 81.9 ± 5.7	Intervention: 25 (83%) male, 5 (17%) female; control: 7 (30%) male, 16 (70%) female	6 hrs, 2–3 days a week	Cognitive functioning (Mini Mental State Examination); functional performance (The Barthel Index); Medication usage^b^; total number of diseases^b^; emotional well‐being^b^; number of clinically relevant behavioural symptoms^b^; medication usage^b^		6 and 12 months
CBA	Elings et al. (2011)	The Netherlands	Mental ill‐health; substance misuse	Day activity projects	113	Intervention: (31–50); control: (31–40)	Intervention: 80% male, 20% female; control: 62% male, 38% female	6 hr, approximately 3 days a week	Social functioning (The Social Functioning Scale); mental functioning (The Mental Health Inventory‐5); appetite and eating pattern (Simplified Nutritional Appetite Questionnaire)	Quality of life (WHOQOL‐brief)	6 and 12 months
UBA	Hassink et al. (2011)	The Netherlands	Disaffected/excluded youth	N/A	74	Male: 16.6 ± 1.0; female: 15.6 ± 1.2	66 male, 8 female	24 hr, for half a year at the farm	Problem behaviour (Youth Self Report); Coping (Utrecht Coping List); self‐determination (IPC LOC Scale)		6 and 12 months
UBA	Gonzalez et al. (2009, 2010, 2011a, 2011b); Gonzalez (2013)	Norway	Mental ill‐health: people with clinical depression	N/A	46	46.3 (25–65)	10 male, 36 female	3 hr, twice a week, for 12 weeks	Positive affect (Positive and Negative Affect Scale); stress (The Perceived Stress Scale); group cohesion (The Therapeutic Factors Inventory Cohesiveness Scale)	Depression (Beck Depression Inventory); anxiety (The State‐Trait Anxiety Inventory–State Subscale)	12 weeks and 3 months after the intervention (i.e., 6 months)
UBA	Pedersen et al. (2011)	Norway	Mental ill‐health: people with clinical depression	N/A	14	37.4 (23–54)	3 male, 11 female	1.5–3 hr, twice a week, for 12 weeks		Depression (Beck Depression Inventory); anxiety (State‐Trait Anxiety Inventory‐State Subscale)	12 weeks
UBA	Javed et al. (1993)	Pakistan	Mental ill‐health: diagnosis of schizophrenia	N/A	25	28.18 (20–60)	25 male, 0 female	–	Mental status (Brief Psychiatric Rating Scale); Rehabilitation (Morningside Rehabilitation Status Scale)		1, 2 and 3 year
UBA	Hine et al. (2008b, 2008c)	UK	Mental ill‐health; substance misuse; older people; offenders	N/A	72	(16–65)	55 (76%) male, 17 (24%) female	5.5 hr (2–8)	Self‐esteem (Rosenberg Self–Esteem Scale); mood (Profile of Mood State Questionnaire)	Depression (from the Profile of Mood State Questionnaire)	Immediately after the intervention
UBA	Hine et al. (2009)	UK	Mental ill‐health: asylum seekers and refugees, who are suffering from PTSD and depression	N/A	20	–	–	10–12 weeks		Quality of life (CORE OM)	End of intervention (10–12 weeks)
UBA	Lambert (2014)	UK	Learning difficulties; mental ill‐health: anxiety and/or depression; psychosis; personality and/or social issues; people with brain injury	N/A	83	40.7 ± 12.8	54 (65%) male, 29 (35%) female	11.5 days	General health and attitude^b^; occupational functioning^b^	Quality of life (EQ‐5D)	End of intervention and AM/PM
UBA	Marshall and Wakeham (2015)	UK	Offenders	N/A	10	–	–	5 hr and 15 min, over 18 sessions	Reoffending		12 months
UBA	Suprise (2013)	USA	Youth in foster care	N/A	18	(9–17)	18 male, 0 female	Up to two times per week with many coming for years	Prosocial behaviour and social competence^b^		6 weeks

Abbreviations: BSI, Beck Depression Inventory; CBA, controlled before and after study; CF, care farm; PTSD, posttraumatic stress disorder; RCT, randomised controlled trial; STAI, State‐Trait Anxiety Inventory; UBA, uncontrolled before and after study.

^a^ Work ability is a composite score comprised intensity and exactness based on observational measurement.

^b^ These outcomes were not included in the logic models as the measure was not defined or the outcome had been modified without adequate description or validation.

#### 6.1.3 Identified theories for inclusion in the logic models

From the 26 theory publications we identified 17 theoretical or philosophical concepts quoted in connection to care farming. Nine had been applied within the included empirical studies, while the remaining eight had been mentioned alongside descriptions of care farming. Of these 17 theories, 15 offered a potential explanation for how care farms might work to bring about change in various client groups (Table 3). The most commonly applied theoretical concept mentioned in studies was the recovery model (mentioned in four studies) (Anthony, 1993). Two concepts were philosophical rather than theoretical and did not offer a mechanistic explanation for how care farming might contribute to well‐being; namely, “existential issues” and “anthroposophy” (O'Connor & Chamberlain, 1996; Steiner, 1925). These were excluded from the process of developing a theoretical framework.

**Table 3:** Description of theories

Name of theory (reference) [included empirical study that refers to this theory]	Description	Theoretical concepts
Attention Restoration Theory (Kaplan & Kaplan, 1989) [1 study: Leck et al. (2015)]	According to Kaplan and Kaplan, there are two kinds of attention: involuntary attention and directed attention. Involuntary attentions requires no effort, while directed attention requires a person to exert effort to avoid other distractions. For some people, the frequent use of directed attention to focus can contribute to mental fatigue. Consequently, a person without directed attention is more likely commit “human error”, feel distracted and be less competent. Kaplan and Kaplan hypothesise that resting ones directed attention could recover a person who is experiencing mental fatigue	Restorative effects of nature
Psychoevolutionary Theory (Ulrich, 1983) [1 study: Leck et al., 2015]	Ulrich argues that being in contact with nature can reduce stress. He argues that affective reactions (i.e., feelings) precedes cognitive responses. An affective reaction is an immediate emotional response, that is naturally triggered such as joy, like or dislike. The affective reaction shapes the subsequent conscious processing, physiological responding and behaviour. According to Ulrich, natural settings triggers positive affective reactions, followed by positive physiological response or positive behaviour	Restorative effects of nature
Biophilia (Wilson, 1984) [2 studies: Pedersen et al., 2012a and Leck et al., 2015]	Biophilia is a fundamental and biologically based human need and a propensity to affiliate with life and lifelike processes. According to Wilson biophilia is inherent in every person, put another way, it is a biological need. Biophilia is part of people's evolutionary heritage (i.e., our ancestors evolved in natural environments)	Restorative effects of nature
Presence Theory (Baart, 2001; Droës & van Weeghel, 1994; Kal, 2002)	Caring involvement in response to the need for intimacy and involvement. People thrive on company but feel isolation if they lack intimacy. In presence approach, the “carer” offers a way out of isolation through being a caring presence. There are no hierarchical differences, no particular goal or intervention/treatment route… Care worker is just “attentively present”. It requires trust, meaningful relationships, where client feels seen and counted. It is about being there, being together, doing things together	Being socially connected; mental Well‐being
Social Support and Social Interactions (Cobb, 1976; House, 1981) [1 study: Ellingsen‐Dalskaua et al., 2016]	There are four main domains of support. Informational support includes giving advice, information and instructions. Emotional support is about having concern, listening and providing trust. Appraisal support involves affirmation and feedback and is likely to be a part of the contact between the farmer and the participant. Instrumental support is practical support and in the case of care farming the provision of, for example, tools, food and equipment. Social support is information which lets us feel cared for and loved; esteemed and valued; a member of a network of mutual obligations. Having social support facilitates coping with crisis and adaptation to change. Since humans are innately drawn to animals, animals serve as a medium through which social interactions can transpire	Being socially connected; mental well‐being
Self‐efficacy Theory (Bandura, 1977)	Expectations of personal efficacy (i.e., the conviction that one can successfully execute the behaviour required to produce the outcome) can be derived from:	Personal growth
	● Performance accomplishment: repeated success ● Vicarious experience: seeing others perform ● Verbal persuasion: telling them what to expect ● Emotional arousal: achieved through participant modelling or cognitive re‐evaluation	
SHIFT‐Desistance Theory (Evans & Evans, 2014) [1 study: Marshal & Wakeham, 2015]	Long‐term abstinence from criminal behaviour can be achieved by:	Personal growth; mental well‐being; being socially connected
	Building and sustaining hope Recognising and developing people's strength Respecting and fostering agency Improving social capital Developing human capital Recognising and celebrating progress	
Salutogenic Theory (Antonovsky, 1979, 1996) [1 study: Schreuder et al., 2014]	Having a positive outlook or optimistic attitude contributes to better health. The SOC is used to explain why some people remain healthy under stress. The SOC includes three dimensions:	Personal growth; mental well‐being
	Comprehensibility: believe that the challenge is understood Manageability: believe that resources are available to cope Meaningfulness: believe that the challenge is worthy of commitment It is hypothesised that people with higher SOC scores are more able to remain health under stress	
Spiritual Experience Process Funnel (Fox, 1999)	When people start to feel relaxed in wilderness they become open to opportunities for spiritual experience and become more connected to nature. Over time this spiritual experience can develop into spiritual growth which can contribute towards significant changes in attitude and adoption of new behaviours	Restorative effects of nature
Recovery Model (Anthony, 1993) [3 studies: Granerud and Eriksson, (2014) and Elings et al. (2011); Hassink, 2009; Hassink et al., 2010, Iancu et al., 2014; Kogstad et al., 2016)	This is a person‐oriented perspective whereby people with mental disorders go through a personal journey and adapt to a new status quo and learn to find personal meaning despite and beyond the limitations imposed by their mental ill‐health:	Being socially connected; personal growth; mental well‐being
	● Moratorium: denial of the mental diagnosis, confusion, helplessness ● Awareness: awareness of a possible identity beyond that of a “sick person” ● Preparation: focus on one's values, strengths and weaknesses ● Rebuilding: actively pursuing a positive identity, stablishing goals and taking responsibilities ● Growth: living beyond disability and being resilient	
Ecological Model of Aging (Lawton & Nahemow, 1973)	Through providing an environment that is a good fit with needs/abilities. Purports that this is achieved through an environment that is compensatory, constant, predictable and stimulating (Lawton, 1989)	Being socially connected; physical well‐being
Attachment Theory (Bowlby, 1969)	Aims to address trust and security issues. Through the use of animals to create healthy attachments and promote development of prosocial behaviours by restoring a sense of trust and security in interpersonal relationships	Being socially connected
Intentionally designed experiences (Sheard & Golby, 2006)	Taken from adventure playground literature but considered that green are activities are examples of IDEs with engagement with the natural world working at all levels: looking at nature, being active in nature, shaping nature and interacting with animals and the IDEs conceptualise how activities provide a chain of events where care farms are vectors for health benefits including first order outcomes achievement, restoration, resilience and empowerment and second order outcomes stress reduction, self‐efficacy, identity formation and social support	Restorative effects of nature; mental well‐being; being socially connected, personal growth
Therapeutic Landscape Concept (Gesler, 1992) [1 study: Kaley, 2015]	A therapeutic landscape is one win which “physical and built environments, social conditions, and human perceptions combine to produce an atmosphere which is conductive to healing…healing induces cure in the biomedical sense (physical healing), a sense of psychological well‐being (mental health) and feelings of spiritual renewal (spiritual healing)”	Restorative effects of nature; mental well‐being
Behavioural theory (Lewinsohn, 1974)	Certain environmental changes and avoidant behaviours inhibit individuals from experiencing environmental reward and reinforcement and subsequently leads to the development of depressive symptoms. By encouraging individuals to take part in activities that create a sense of pleasure or mastery, avoidant behaviours can be reduced	Personal growth; mental well‐being

Abbreviations: IDE, intentionally designed experience; SOC, sense of coherence.

#### 6.1.4 Characteristics of included qualitative studies

All of the included qualitative studies (See Table 1) were conducted across three European countries (nine in the Netherlands; five in Norway; and three in the UK) and one in the US. Six of the studies focused solely on people with mental health problems, including people with depressive, psychotic or personality disorders. Four studies included two client groups: those with mental health problems and/or drug and alcohol problems. Other studies involving single client groups included two with learning disabilities, two with disaffected youth, and one including people with autism. Two studies included more than two client groups, one of which also included older people. There were 364 service users included in the 18 studies. The maximum study group size was 55 and the minimum was five service users. Included studies also included 100 other participants (such as officials, health professionals, farmers and family carers) where the findings from these groups focused on questions unrelated to the impact of care farms on client groups they were not included in the synthesis within this review. Most of the studies used individual interviews (*n *= 12) as the sole method for data collection. Two used focus groups, two combined focus groups and individual interviewing, and a further two included video and photography combined with interviewing. There were three studies that involved “significant others” in eliciting the experiences and effects of care farming from service users with communication difficulties. In the first of these three studies, the sole source of information was care farmers (Ferwerda‐van Zonneveld, Oosting, & Kijlstra, 2012). In the second study, limited information was gathered from service users (Elings, 2004), and in the third study, accounts of farmers, carers and parents supplemented the visual elicitation methods adopted by the researcher (Kaley, 2015). Ten studies failed to provide information on the age of the study participants, and gender was not reported in five studies. Excluding those studies where gender was not reported, there were almost twice as many male service users participating in the studies as females (ratio of 1.8:1).

#### 6.1.5 Characteristics of included quantitative studies

The 13 studies were conducted in five different countries: four in Norway; four in the UK; three in the Netherlands, and one each in Pakistan and the United States (see Table 2). There were two RCTs and three controlled before and after studies (CBAs), with the remaining nine using an uncontrolled before and after design (UBAs). The two RCTs involved single target groups, both focusing on mental illness. Ten studies evaluated the effects of care farming on a targeted single client group: six were on service users with mental health problems; two on disadvantaged youth; one on older people with dementia and one on offenders. Within the mental illness studies, three specifically focused on clinical depression, with the remaining studies including a range of disorders, including schizotypal and affective disorders, posttraumatic stress disorder and psychosis. Three studies used a mixed client group, with two including four different groups. Data on ratio of male to female participants attending the care farms were provided in 10 studies (one of which provided percentages rather than numbers); there were more than twice as many males compared to females (*n *= 261 males; 117 females). The mean ages of participants in the studies ranged from 9 to 78 years. However, age was not reported in two studies (Hine, Barton, & Pretty, 2009; Marshall & Wakeham, 2015).

The intensity and duration of interventions varied, but most commonly involved half day (1.5–3 hr) or full day (5–6 hr) sessions two to three times per week over a 12 week period. In the two studies involving disadvantaged youth (Hassink et al., 2011; Suprise, 2013), the duration of intervention was substantially longer, with one study mentioning 6 months and the other with an open‐ended contract. Studies involving service users with mental health problems most commonly stated a 12 week intervention period.

##### Data collection time points

One CBA study involving offenders on a community order completed follow‐ups mostly just prior to the end of the intervention to maximise retention in the study (Elsey, Murray, & Bragg, 2016). Four UBA studies (Hine et al., 2009; Hine, Peacock, & Pretty, 2008b; Lambert, 2014; Pedersen, Nordaunet, Martinsen, Berget, & Braastad, 2011) performed follow‐ups immediately after the intervention. The RCTs reported follow‐ups at 6 months (from baseline) (Berget, Ekeberg, Pedersen, & Braastad, 2011; Pedersen et al., 2012b). The remaining studies reported outcomes at 12 months (four studies), 6 months (two studies) and 3 months (one study). Only one study did not report the time point of follow‐up (Suprise, 2013). The longest follow‐up period reported was three years from a UBA study (Javed, Chaudhry, Suleman, & Chaudhry, 1993) involving service users with mental health problems; however, the duration of the intervention was not provided.

##### Outcomes

Twenty four different defined outcome measures were applied across a spectrum of psychological, social, cognitive and physiological domains; six measured primary outcomes, 17 measured proximal outcomes and one included measurement of both a proximal and as part of a subscale of a primary outcome). The maximum number of validated outcome measures applied within a single study was eight, with a mean number of four measures across the 14 studies. In addition to these reported defined outcome measures, four studies also reported eight outcomes without naming or providing adequate description of the measures. These were excluded from the analysis.

Four measures were used across four studies to measure quality of life: Norwegian version of Quality of Life Scale, WHOQOL‐brief, CORE‐OM and EQ5D. Anxiety was measured in four studies (two RCTs and two UBA studies) using a single outcome measure (State Trait Anxiety Inventory); this probably reflects the fact that three of the studies involved the same authors. Similarly, two measures of depression (Beck Depression Scale and a subscale within the Profile of Mood State) were applied across five studies. Again, three of the four studies using the Beck Scale were written by the same authors.

Proximal outcomes included: coping (measured in two studies using different measures), self‐efficacy, cognitive functioning, functional performance, number of clinically relevant behavioural symptoms in dementia, social functioning, mental health and well‐being, mental functioning, appetite and eating pattern, self‐esteem, positive effect, stress, group cohesion, mental status, psychiatric rehabilitation, mood and reoffending.

#### 6.1.6 Contextual information about care farming interventions

Three included studies did not provide contextual information about either the contents of the intervention or the organisational set‐up (Ferwerda‐van Zonneveld et al., 2012; Lambert, 2014; Leck, Upton, & Evans, 2015). Across the client groups there were no obvious differences overall in the types of activities undertaken. This may, however, reflect the lack of detail provided about the interventions within the papers (Table 4). For example, the physicality of the work is likely to vary according to age, physical ability and mental health, but some studies only mentioned working with animals as an activity. The types of activities reported on care farms fell into four categories:

- Horticultural or land maintenance work—in addition to the more traditional growing of vegetables and fruit, activities also included hedge cutting, conservation work, tree planting and mending fencing. All client groups were reported to have participated in these types of activities.
- Conventional farm animal care—this involves working with animals traditionally associated with farming (e.g., cows, sheep and pigs). There were some examples where the work was truly agricultural, emulating the role of the farmer (Berget, Skarsaune, Ekeberg, & Braastad, 2007; Marshall & Wakeham, 2015), whereas with others the emphasis seemed to be about just being in contact with the animals, and interacting but without real agricultural purpose (Little Gate Farm, 2015). Some studies suggested farms offering both ways of working with animals, depending on the abilities of the client (Berget et al., 2007).
- Additional farm animal‐based activities—beekeeping, fish farming, maintaining a mini zoo and working with donkeys.
- Other activities—these included working in the shop, outdoor recreational activities (camping, campfires, outdoor trips and den building) and indoor activities (baking, meal preparation, crafts, games, general household work and tractor driving).

**Table 4:** Descriptions of care farming interventions

Study	Background information about the farm	Activities	Contractual arrangements
Mental ill‐health/substance misuse			
Javed et al. (1993), Pakistan	A therapeutic community providing suitable programmes to achieve better management goals for chronic schizophrenics in the community	Fish farming, poultry farming, teddy goat farming, mini zoo, maintenance of honey bee houses, gardening and cultivation of crops and vegetables	No details
Berget et al. (2008); Berget et al. (2007), Norway	Study includes a number of farms. It is unclear if this is an existing animal assisted therapy intervention to support psychiatric patients or whether it was established just for the purpose of the research study since only two of the 15 farmers had experience of psychiatric patients prior to the study	The main productions were dairy cows, specialised meat production with cattle, sheep or horses. All dairy farms had meat production with cattle in addition. Some also had sheep or horses. All farmers had small animals like rabbits, poultry, pigs, cats or dogs as part of the far. The patients were only working with the animals, performing ordinary stockman work under supervision of the farmer; they were not allowed to do other kinds of farm work. The farmers were told that the work should depend on the patient's coping ability interest, and that patients should have opportunity for physical contact with the animals, for example, patting, brushing, washing; moving the animals between different places in the cowshed; feeding adult animals, or milk feeding the small animals; cleaning the cowshed or washing buckets and bottles; milking	No details
Bjørgen and Johansen (2007), Norway	Involves a number of farms that aim to help people with mental ill‐health	Tending to livestock and vegetable gardens, baking, carpentry, mountain trips or visits to other farms. Every day starts with a cup of coffee and discussing the day's tasks, and every farm has group lunches	The programme is contracted by the relevant municipality in Norway or the Labour and Welfare Administration from farms that are prepared and willing to offer it
Elings (2004), The Netherlands	No details	Working on the farm, caring for animals (pigs and cows), making cheeses, picking eggs caring for hens, horticultural activities	No details
Elings et al. (2011), The Netherlands	Aim depending on the client but mostly: day‐activity, resocialisation, work rehabilitation. Study involved 44 different farms offering different work activities	Limited information on activities. Next to the agricultural production, farms often have more multifunctional activities like: a farm shop, camping site or nature conservation	Some farms have collaborate with a health care institution. Some have an individual AWBZ^a^ accreditation. Some farms have an antroposophical or Christian background. Funding can also be through personal budget
Gonzalez et al. (2010), Norway	No details provided	Therapeutic horticulture. Active and passive participation in gardening activities. The active parts of the programme included sowing, seed germinating, potting, planting and cultivating vegetables, flower and herbs. The passive parts included sitting on benches, and watching and listening to birds, the weather and the landscape	No details provided
Granerud and Eriksson (2014), Norway	Enabling people with physical, mental and emotional functional limitations of different kinds to integrate socially. Study focused on people with mental ill‐health and substance misuse	Specific to the study participants, activities often performed in small groups, with each group taking turns engaging in different types of work on a farm (can include looking after animals, cutting wood or working with plants) or in a farmhouse, such as cooking for all members of their group, laying the table or washing up dishes	
Hine et al. (2009 2008b), UK	Study involves seven farms providing a wide variety of activities. Individual aims of farms not reported	Activities varied widely but included: cleaning out turkeys and put fresh straw down; working with the donkeys, sheep and horses; feeding and grooming horses; weeding; taking fences down: planting trees; cleaning out stables; milking; mixing animal feeds; collecting eggs; feeding and watering cows, pigs, goats; mucking out; farm maintenance	No details
Hine et al. (2009), UK	As an urban farm it aims^a^ to provide educational, recreational and therapeutic activities that: (a) support disadvantaged and disabled people to boost their confidence and aspirations; (b) promote emotional, mental, social and physical well‐being; (c) develop environmental awareness and action; (d) strengthen community cohesion; (e) create enjoyment for members of the public	Psychoeducation, movement therapy, sharing food and gardening	
Iancu et al. (2014) The Netherlands	13 farms chosen for study. No other details about individual aims provided	Most activities on the farms were related agricultural production; training of users for integration into the labour market in two farms and other daytime activities for people living under supported housing (*n *= 1). On the 12 private farms, supervision was provided by farmers (*n *= 3), by farmers previously trained as mental health nurses or social workers (*n *= 4), by professional activity supervisors (*n *= 3) or by both trained farmers and professionals (*n *= 2)	One care farm was owned by a mental health organisation, and employed a farmer and several professional activity supervisors for the guidance of users. The 12 remaining care farms were all privately owned and run by farmers and their families
North Essex Research Network & South Essex Service User Research Group (2013), UK	The service aimed to work with the service users to build their resilience, develop their skills and support them to establish a meaningful life	Working in the woodland, ice cream making; painting the fences; camp fires; grass cutting; working with animals	Placements commissioned as a pilot study by the local then PCT
Pedersen et al. (2011, 2012a, 2012b); Norway	Three separate studies but all involved between 6 and 8 dairy farms from different counties	Milking, feeding, fetching feed, cleaning, moving animals, milking/feeding calves, handfeeding animals, technical preparation before feeding, grooming, mucking, physical contact with animals, observing animals, inactivity, dialogue with the farmer, talking to the animals, taking care of the calves. They could also choose to spend their time in physical contact with the animals	No details
Learning disabilities			
Baars et al. (2009) The Netherlands	Described as a therapeutic work and living community which is part of a health care institute providing therapy and clinical day activity and treatment	Farm work and other related activities like working in the farm shop, working in the household and kitchen and do odd jobs like, for example, cutting wood	Taking people with psychiatric problems but with no psychosis. Funding through part of general health costs.
Offenders			
Marshall and Wakeham (2015), UK	To provide a range of activities that encourage participants to value learning, including: build a prosocial drug free lifestyle, increase self‐confidence, improve interpersonal skills, develop their own potential, challenge their current norms and behaviours, support reduction of and abstinence from the use of illegal substances. They register all participants so that they can achieve National Open College Network qualifications	Dagging sheep, building walls, delivering lambs, rounding up, feeding and managing sheep and cattle, shearing, littering pens, tractor driving, ploughing, cutting weeds and hedges, investigating wildlife in ponds and rivers	Contract through local probation service.
Disengaged youth			
Hassink et al. (2011), The Netherlands	Decrease of behavioural problems, less recidivism, less substance abuse, fewer appeals to youth care, back to school or work, restoring contact parents/enhanced contact parents, restore daily schedule, improving choice of friendships	The concept consists of three steps. (1) Survival. (2) Stay on the farm (living and working). During this stay on the farm the young people have to: take care of their residential unit; write a dairy; learn to listen to the farmer and do assignments. (3) Guidance—not otherwise specified	
Kogstad et al. (2014), Norway	Offers employment schemes for youth to improve their opportunities for entering the workforce or to aid them in continuing their education	Feeding and caring for the animals, cleaning the stable, weeding the vegetable garden, splitting firewood	Employment schemes are financed by the labour and welfare sector
Schreuder et al. (2014), The Netherlands	General objectives of the programme are to develop more positive perspectives in the domains of “functioning” (e.g., school, work or family life), while developing a workable relationship between youth and parents	Living and working on the farm (6 months) followed by a 6 month aftercare programme. Actual farming activities are not described	No details
Suprise (2013), USA	To end the cycle of violence by creating a “truly humane society” and through activities and teaching help children to learn empathy	No details	Referrals via welfare reform agencies. Many of the referred foster youth have mental ill‐health diagnosis; some of the most common include PTSD, depression, anxiety and attachment disorders
Older people/dementia			
Schols and van der Schriek‐van Meel (2006), The Netherlands	Aim is similar to institutional day care which is to offer extra care and meaningful activities, increase well‐being; offer respite, alleviating some of the burden of family caregivers, social network and homecare services; and prevent or postpone nursing home admissions. Includes the concept of normalisation or socialisation of chronic care with its aim to enable people to live their lives in their own homes for as long as possible	Working in the garden, sowing and harvesting their own vegetables, helping to prepare their own meals, using their own vegetables, taking care of the animals	The care farm was operationally entrusted to a nursing home. Nursing staff are employed on the farm
De Bruin (2009); De Bruin et al. (2012), The Netherlands	Aim to provide an adequate day structure and a meaningful day programme to frail and/or community dwelling elderly people, so as to prevent social isolation and to offer respite care to informal caregivers at home	Activities do not contribute to agricultural production. They include farm or animal related activities (watching or feeding animals, cleaning pens and cages, picking eggs); garden or yard related activities (sweeping yards, gardening, working in greenhouse); games (party games, memory games, quizzes, billiards, shovelboard); crafts (flower arranging, decorating postcards, knitting, making nest boxes, sanding or painting fences); other leisure and recreational activities (dancing, singing, gymnastics, going for a walk, reading, participating in group discussions); domestic activities (peeling potatoes, chopping fruit and vegetables, laying the table, dish washing, shopping); sitting or pottering while watching and/or chatting (no involvement in organised activity); resting (sleeping or napping in chair or in bed)	Farms are often co‐operatives with regular health care institutions. Their services are financed by the Dutch national insurance system
Mixed groups			
Little Gate Farm (2015), UK	To enrich the lives of children with special needs and give them the opportunity to gain independence and confidence, while at the same time having a lot of fun and learning lot of new things, such as farming, animal care and where food comes from. To support learning disabled adults to learn practical farm and woodland skills	Farming, animal care, animal feeding and handling, making our own pizza dough bases and topping; chick cleaning and holding, craft (making bird feeders and bird cake, decorating a flower pot and planting a sunflower); woodland den building; animal cleaning and feeding. Animal care, horticulture, woodland management, traditional skills, enterprise and conservation	Charity funding
Iancu et al. (2014), The Netherlands	Five farms studied in detail with varying aims: (a) To provide day time occupation to residents of supported housing; (b) to ensure an enjoyable workplace with social and work skills can be learned; (c) to provide work and facilitate reintegration between users and the community; (d) to provide an enjoyable structured workplace that facilitates social interaction; (e) to provide a safe environment for work, contact with animals and opportunities to be outside	Three farms—dairy production; three farms—selling produce in farm shop; two farms—farm work; two farms—taking care of animals; remaining only reported in individual farms—pottery, textiles, carpentry, maintenance of equipment, guiding school groups, serving service users in a cafe	Two institutional farms (owned by healthcare organisations); two contracted farms (private care farms working in collaboration with health care organisations); one independent farm (financing their service through personal budgets of service users)

*Note*: AWZB accreditation—The Dutch “general law on exceptional medical expenses” (AWBZ) provides general insurance covering special health care needs. Care is either provided “in kind” through certified health care institutions or can be hired by clients through a personal budget. Some farms in the Netherlands are registered as certified health care institutions (Elings, 2007). Studies in which no details about the intervention included: Leck et al. (2015); Di Acova (2013); Ferwerda‐van Zonneveld et al. (2012); Lambert (2014).

Abbreviation: PTSD, posttraumatic stress disorder.

^a^ Information obtained from farm website: http://vauxhallcityfarm.org/about/what‐we‐do/.

There was a general lack of information regarding contractual arrangements of care farms. A range of models were in place: care farms as part of a nursing home or mental health care organisation; privately‐owned farms working in collaboration with health care organisations (the Netherlands and UK) or the welfare sector (Norway) or probation (UK); and privately owned farms with income generated through personal budgets, charitable donations or grants.

#### 6.1.7 Excluded studies

One hundred fifty‐one studies were excluded after examining the full text. Four excluded studies consisted of single subject studies. Eight studies were excluded because the participants were not from a vulnerable or disadvantaged population; for instance, the participants were school children visiting a farm for educational purposes. Twenty‐four studies were excluded because the studies did not meet the care farming definition. Some studies classified activities as “therapy” rather than activities that are therapeutic, so we excluded four studies. Twelve studies were excluded on the grounds of setting; these studies were not delivered at a farm, but instead at a prison or a hospital. Four studies were excluded because the intervention exclusively consisted of single activities such as gardening or horse riding. Some studies combined different interventions, for example, care farming activities combined with learning music at a recreation centre. For these studies, it was difficult to separate the true effect of the care farms, so three studies were excluded. Two studies consisted of “one‐off” educational visits to the CF and were excluded. Eighty‐five studies were excluded because they were reviews, overviews, surveys, commentaries or editorials. Five PhD theses were excluded because their findings had been subsequently published elsewhere and the peer‐reviewed publication was included in this review.

### 6.2 Risk of bias in included studies

#### 6.2.1 Qualitative studies

Nine studies (50%) fully met more than 50% of the 37 quality assessment criteria (Table 5). Two studies (Ellingsen‐Dalskaua et al., 2016; Pedersen et al., 2012a) met more than 60% of the criteria. One study (Baars, Elings, & Hassink, 2009) met <20% of the criteria.

**Table 5:** Qualtiy assessment of qualitative studies

Section of tool (number of items)	Items most often addressed (number of studies plus number partially addressing item)	Items least often addressed (number of studies, plus number partially addressing item)
Background, research team and reflexivity (8)	Is it clear what is being studied (18) Is it clear which author(s) conducted the interviews or focus groups? (10, plus 1) Is the gender of the researcher clear? (10, plus 1)	Were the characteristics of the interviewer reported? (1) Evidence of relationship established between researcher/interviewer and participant before the study commenced? (1 plus 1) Did the researcher/interviewer indicate if there was a pre‐existing relationship with the participant and if so, was this described? (1)
Study design (16)	Does the study state how many took part in the interviews/focus group/observations? (15 plus 1) Does the author say how many interviews/focus group/observations were carried out? (13) Was audio or visual methods used to record/collect the data? (12)	Does the researcher state if anyone else was present during the interviews? (5, plus 1) Was data saturation discussed? (4)
Data analysis and findings (13)	Do the quotations reflect the findings? (16) Were major themes clearly presented in the findings? (18)	Does the study report the number of coders involved? (5) Did the authors report checking back with informants over interpretation? (3)

Clarity about the nature of the investigation, the presence of quotations reflecting the findings, and the presentation of clear major themes were the criteria most often addressed. Conversely, openness about the researcher's bias and assumptions, and evidence of pre‐existing or newly established relationships were only addressed by one study each. Two criteria fundamental to all research practice are evidence of ethical approval and of informed consent. These were not reported in nine (50%) studies.

We observed that eight of the ten studies that met (fully or partially) more than 50% of the quality criteria used a theoretical framework. Conversely, only one (Leck et al., 2015) of the eight studies scoring <50% in the quality assessment used a theoretical framework. The implications this might have on the quality of the results are unclear. Studies involving service users with mental health problems that used the recovery model reported greater variability in the extracted findings, specifically the range of mechanisms, compared to those who did not use a framework.

#### 6.2.2 Quantitative studies

All the included quantitative studies had many limitations and were assessed as having a high risk of bias. A summary of the risk of bias of the quantitative studies can be found in Tables 6 and 7.

**Table 6:** EPOC risk of bias tool for randomised controlled trials

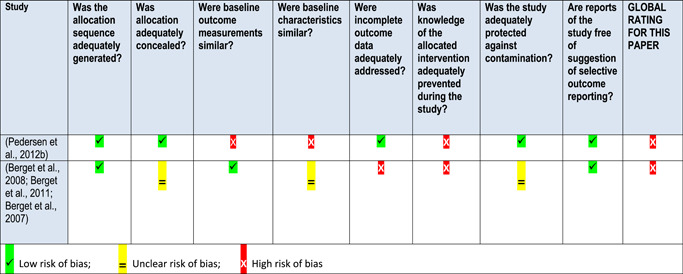

**Table 7:** EPHPP risk of bias tool for CBA and UBA

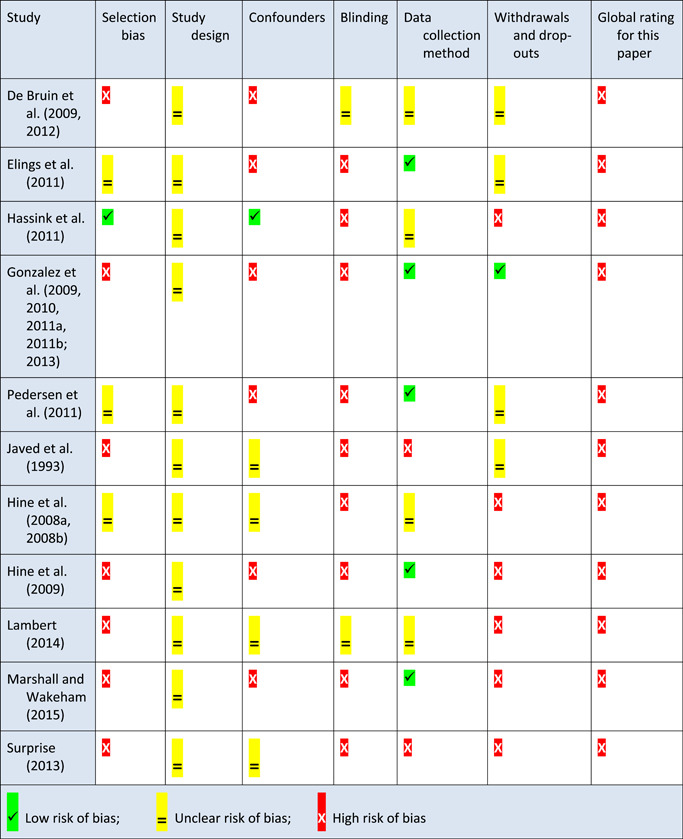

Abbreviations: CBA, controlled before and after study; EPHPP, Effective Public Health Practice Project; UBA, uncontrolled before and after study.

##### Randomised controlled trials

###### Allocation

The method of random sequence generation was described clearly in both RCTs. For example, Berget et al. (2008) used computer‐generated random numbers. However, only one study clearly described the allocation concealment. Berget et al. (2008) did not address allocation concealment whereas in Pedersen et al. (2012b) randomisation was conducted by a researcher blinded to farm and participants.

###### Baseline outcomes

Patient outcomes were measured at baseline in both studies, and one study reported no important differences across intervention groups (Berget et al., 2008). However, Pedersen et al. (2012b) reported higher depression scores and anxiety scores in the control group at recruitment, and higher self‐efficacy scores in the intervention group at recruitment, but these differences at baseline were not adjusted in the analysis.

###### Baseline characteristics

Pedersen et al. (2012b) reported differences in baseline characteristics between the intervention and control groups. For example, there were more men, and participants were older and better educated in the intervention group. It is unclear whether the baseline characteristics were similar in the study conducted by Berget et al. (2008). For instance, some characteristics are mentioned in text, but no data were presented for the intervention and control groups separately.

###### Incomplete outcome data

Both studies reported attrition rates and the number of participants excluded from the analysis. In both studies, proportionally more people dropped out of the CF arm than in the control arm: 32% and 50% (Berget et al., 2011, 2008) versus 7% and 15% (Pedersen et al., 2012b). It should be noted that in the latter study (Pedersen et al., 2012b), the number of included participants were very small (*n *= 29), the control group was a wait‐list group, and half of those dropping out of the CF arm did so before the intervention started. The reasons for drop out were little interest in animals and boredom (Berget et al., 2008). Furthermore, it was reported that significantly higher drop‐out rates were observed in those using sleeping medication (*p *= .05), and hospitalised patients (*p *= .006) (Berget et al., 2008).

###### Blinding

Primary outcomes variables were not assessed blindly in both studies (Berget et al., 2007; Pedersen et al., 2012b). This was reported as a limitation in the discussion section of both studies.

###### Contamination

Pedersen et al. (2012b) used a wait‐list control group and it is unlikely that the wait‐list control group received the intervention prior to the intervention group. However, it is uncertain whether there was contamination in Berget and colleagues’ study. They report that the control group received treatment as usual, but do not give any additional description.

###### Selective outcome reporting

There was no evidence that the outcomes were selectively reported in both studies; for instance, all the outcomes described in the methods section were reported in the results section (Berget et al., 2008; Pedersen et al., 2012b). Neither study published a protocol detailing outcomes to be measured a priori.

##### Controlled before and after studies and uncontrolled before and after studies

###### Selection bias

Only one study had selected individuals that were likely to be representative of the target population. Three studies had selected individuals that were somewhat likely to be representative of the target population, for example, through referral from clinicians in a systematic way. Seven studies did not use a systematic process to select individuals.

###### Study design

We assessed the likelihood of bias due to the allocation process; all eleven controlled before and after studies and uncontrolled before and after studies were rated at moderate risk of bias as the investigators did not use a robust process to select participants.

###### Confounders

Only one study controlled for at least 80% of relevant confounders. Four studies controlled for approximately 60–79% of relevant confounders. Six studies either controlled <60% of relevant confounders or did not report any confounders.

###### Blinding

In the majority of studies (nine studies), the outcome assessors were aware of the intervention status of participants. Two studies did not describe blinding.

###### Data collection method

Five studies used valid and reliable tools to collect data. Four studies did not describe the reliability of the data collection tools and two studies did not describe the data collection tools used to measure outcomes.

###### Withdrawals and drop‐outs

Only one study reported a follow‐up rate >80%. Four studies reported follow‐up rates between 60–79%. Six studies either reported follow‐up rates <60% or failed to report withdrawals and drop‐outs rates.

### 6.3 Synthesis of results

#### 6.3.1 Stage 1: development of a preliminary theoretical framework

##### Theories and theoretical concepts

Theories (see Table 3 for a complete list and descriptions) differed in scope and in the extent to which they explained causation, thus contributing to the development of a theoretical framework in different ways. With regards to scope, some theories provided a rich, focused, description for how multiple but seemingly disparate dimensions of life could combine to produce a specific outcome. For example, the recovery model (Anthony, 1993) describes aspects of identity, achievement and social connectedness for improved mental health. In comparison, the ecological model of aging (Lawton & Nahemow, 1973) starts with a broader premise and offers a more superficial description of possible mechanisms drawing again on disparate entities, but with a set of defined outcomes relating to cognition, psychology and physiology rather than just one.

Some theories were complex, and so mapping the farming mechanisms derived from the qualitative studies to them in their original state was impractical. Instead we distilled out the key concepts from each theory and identified areas of overlap, which enabled us to transition from 15 theories to five theoretical concepts. The final concepts and the theories from which they are derived are listed below:

- Restorative effects of nature: (attention restoration theory, the biophilia hypothesis, psychoevolutionary theory, Spiritual Experience Process Funnel theory, intentionally designed experiences and therapeutic landscape concept).
- Being socially connected: through belonging and friendships (social support theory, attachment theory, ecological model of aging, recovery model, presence theory, desistence theory/SHIFT, therapeutic landscape concept and intentionally designed experiences).
- Personal growth: through increasing confidence, self‐efficacy, sense of achievement, spiritually, empowerment, having a better identity and being positive (self‐efficacy theory, desistence theory/SHIFT, salutogenic theory, intentionally designed experiences, recovery model and behaviour theory).
- Physical well‐being: improving or maintaining physical activity (ecological model of aging).
- Mental well‐being: coping, cognitive stimulation, meaningful life (salutogenic theory, social support theory, behavioural theory, ecological model of aging, SHIFT desistance theory, recovery model, presence theory, intentionally designed experiences and therapeutic landscape concept).

##### Primary outcomes suggested by the theories

Primary outcomes explicitly suggested by these theories are depression and anxiety (behaviour theory; recovery model; SHIFT desistance theory) and quality of life (salutogenic theory).

##### Proximal outcomes suggested by the theories

Proximal outcomes suggested by theories relate to confidence (SHIFT desistance theory), stress (attention restoration theory; psychoevolutionary theory; intentionally designed experiences), coping (social support theory; SHIFT desistance theory) and self‐efficacy (self‐efficacy theory), prosocial behaviours (attachment theory; SHIFT desistance theory).

This list of outcomes and proximal outcomes is not definitive since arguably many supposed outcomes might actually be part of the mechanisms contributing to the theory. For example, the recovery model talks about being “in work” as part of the recovery from mental illness rather than necessarily seeing it as an outcome in its own right. The aim here is to look at the role of various theories in explaining how care farms might work rather than defining the developing logic models by the theories themselves.

#### 6.3.2 Stage 2: identification of care farming components, mechanisms and proximal outcomes from qualitative studies

Through the process of deconstruction of reported themes, we identified 85 intervention components (grouped into four categories), 164 mechanisms (grouped into 15 categories) and 24 proximal outcomes.

##### Care farming components

Five categories of components were identified (see Table 8):

- Being in a group—comprised mostly positive findings about the benefits of working with other people. Findings included “relatively stable and informal group working”, “working together” and “interacting with different people”. This category also included two negative findings (from two different studies both involving people with mental health problems) about this aspect of the care farming intervention, and these included “not wanting to interact with others” and “finding it challenging to deal with disabled users”.
- The farmer—all findings were positive and related to how the farmer and farm staff supported the service users through the activities they provided and individually. Findings included “being able to express how they felt”, the farming “seeing them as normal” and “providing practical experience”.
- The work—findings relating to the actual activities revealed commonalities, but also diversity in preferences. The pressure of the work was valued in some studies, while in others being able to do work at one's own pace was expressed as important. Doing “real” and varied work was also reported as a benefit. There was one negative finding about “not enjoying some of the tasks because it was a working farm”.
- The animals—none of the findings about animals were negative experiences. Being able to touch, be responsible for and overcoming fear of animals were reported findings in this category.
- The setting—quietness and space to be alone were common features of the setting that service users identified. Being outside and experiencing nature were also reported. There was one negative finding involving mental health service users who felt that they were “on display” because of the educational visits.

**Table 8:** Intervention categories derived from qualitative studies according to client group

Intervention category	Number of findings in each category (%)			
	All client groups	Mental ill‐health and substance misuse	Disaffected youth^a^	Learning difficulties^b^
Being in a group	15 (16)	12 (17)	4 (16)	4 (19)
The farmer	25 (27)	19 (27)	5 (20)	6 (29)
The work	29 (32)	20 (28)	7 (28)	10 (48)
The animals	12 (13)	11 (15)	3 (12)	0
The setting	11 (12)	9 (13)	6 (24)	1 (5)
All	92	71	25	21

*Note*: Twenty‐two of mental ill‐health and substance misuse findings also included disaffected youth and service users with learning disabilities and older people.

^a^ Only five of 25 findings were solely disaffected youth.

^b^ Sevenof 21 findings included service users from other groups.

Overall, care farming intervention components relating to the farmer and the work appeared to be prominent features in the findings (Table 8). Despite the fact that data were infrequently reported for single client groups we did observe some differences in the types of components mentioned that may indicate differences in either the types of activities made available to disparate client groups or the level of importance of those activities to types of service users. For example, studies involving predominantly people with learning disabilities did not mention activities relating to the animals or the setting. However, studies involving disaffected youth reported a preponderance of work and setting related activities.

##### Mechanisms

Through the iterative clustering exercise, mechanism based findings were organised into 15 categories of mechanisms (Table 9 for description of each category). Across the studies the number of findings relating to mechanisms ranged from 3 to 22. In general, theory‐based studies identified more mechanism findings (Table 10). In terms of frequency and spread of findings, “understanding the self”, “social relationships” and “belonging and non‐judgement” represented the most common categories across all studies (represented in bold in Table 10). “Creating a new identity” and the farm as a “distraction” were least often observed across the studies.

**Table 9:** Description of mechanism categories and frequency of findings within each category

Mechanism categories	Description	Frequency of findings in each category (all groups from across all qualitative findings)
Achievement and satisfaction	Working at the farm gives service users a sense of satisfaction. At the farm participants learn to perform activities, hence they spent their days being constructive. There is satisfaction with using their bodies and spending time outdoors	17
Belonging and nonjudgement	The care farm is seen by service users as a place of belonging and mutual acceptance. Feelings of solidarity are created through shared experiences. Service users enjoy working with the animals who are perceived to be nonjudgemental	23
Creating a new identity	service users view themselves in a new light as a worker, principally as a farmer	3
Distraction	The farm creates physical work which offers both a practical and mental distraction from service users own negative thoughts. Conversations centre on work which offers further distraction	6
Feeling valued and respected	Service users feel valued, appreciated and needed by the farmer (and the animals) and consider that they are respected “for who they are”	15
Feeling safe	The atmosphere at the farm creates a feeling of safety and security, providing a mental shield between illnesses and addictions. For some service users this experience is enhanced through physical contact with the animals but for others there is a need to overcome fear of animals which can then lead on to a feeling of safety	12
Learning skills	Care farms give service users the opportunity to learn new skills ranging from growing crops to looking after animals which enables some to gain qualifications enabling then to (re)enter the work place	11
Meaningfulness	Service users perceive tasks as meaningful because they are judged to be useful to others and are *needed* to conduct day to day activities at the farm. service users also see their role as personally meaningful, contributing to society giving them a sense of purpose, happiness and fulfilment	13
Nurturing	Through helping each other and caring for the animals/plants service users become consider of other peoples’ needs and recognise they are doing good for other living creatures	5
Physical well‐being	Through physical activity on the farm service users improve their physical strength. There is a sense of “good” tiredness from physical work. service users start to feel more independent and healthier	10
Reflection	The care farm environment is quiet and peaceful allowing service users to stop and reflect about their problems, their social influences and also the progress they have made. For young people, working at the farm gives space and time away from their family and friends	4
Social relationships	Care farms provide opportunities for participants to interact with the farmer, and other service users. For instance, often service users were working together in groups which helped them to develop their communication skills. As the intervention progressed the service users deepened their relationships with the farmer and considered him as a role model. Once service users gained social confidence, their social networks grew. In particular, they found that in social functions talking about their farm work was more interesting rather than talking about their illness. However, a few service users did not want to interact with others and found it difficult to deal with the diverse range of service users at the farm	17
Stimulation	Service users find tasks stimulating giving them more energy, encouraging a mindful approach to work especially around animals which are unpredictable. Working with animals offers a sensory experience and the energy derived from the work enables them to work through their own problems better. The experience of being in nature is energising	7
Structure	The daily farming activities provided a predictable work environment to the service users. This consistency helped the service users to gain a normal rhythm. Moreover, the farmers also allowed service users to work at their own pace as they understood that the service users can have a “bad day” and may not be able to work at full capacity. Similarly, farmers involved participants in deciding tasks for the day	8
Understanding the self	The care farm environment has allowed service users to better understand themselves. Participant's self‐awareness grew while at the care farm. For example, learning to master an activity at the farm increased their self‐respect and positive self‐image. At the farm, participants were free to be themselves, they also had the opportunity to learn and when they made mistakes they were given time and guidance to learn from their mistakes. This gave them the understanding that tasks at the farm are manageable which enhanced their self‐efficacy and self‐confidence. Some found caring and cuddling animals helped them to deal with problems	26

**Table 10:** Mechanisms and proximal outcomes identified in qualitative studies

References	No. of mechanism findings	Categories of mechanisms	Target groups	Outcomes reported by participants	Theoretical basis
Baars et al. (2009)	16	Achievement and satisfaction; belonging/nonjudgement; distraction; feeling valued/respected; learning skills^a^; nurturing; physical well‐being; social relationships; structure; understanding the self^a^	Mental ill‐health	Less medication	None stated
Bjørgen and Johansen (2007)	5	Distraction^a^; feeling safe; social relationships^a^	Mental ill‐health	Increased confidence, physical health, vocational rehabilitation	None stated
Elings (2004)	20	Achievement and satisfaction; belonging/nonjudgement; creating a new identity; feeling safe; feeling valued/respected^a^; learning skills; meaningfulness; stimulation; structure; understanding the self	Mental ill‐health: service users with intellectual disabilities	Improved self‐esteem	None stated
Elings and Beerens (2012); Elings and Hassink (2008, 2010)	16	Achievement and satisfaction; belonging/nonjudgement; distraction; learning skills; meaningfulness; physical well‐being; stimulation; understanding the self^a^	Mental ill‐health: psychiatric; substance misuse	Increased confidence	Anthroposophy^c^
Elings et al. (2011)	3	Feeling safe; meaningfulness; structure	Mental ill‐health; substance misuse	–	None stated
Ferwerda‐van Zonneveld et al. (2012)	5	Feeling safe; reflection; stimulation; structure; understanding the self	Children with autism spectrum disorders	–	None stated
Granerud and Eriksson (2014)^b^	13	Achievement and satisfaction; belonging/nonjudgement^a^; feeling safe; feeling valued/respected; meaningfulness; physical well‐being; structure; understanding the self	Mental ill‐health: long‐standing severe psychotic disorders, personality disorders; substance misuse	Improved well‐being, self perception; social life and confidence	Recovery Model
Hassink et al. (2010), Hassink (2009)^b^	10	Belonging/nonjudgement^a^; feeling safe; feeling valued/respected; learning skills; social relationships; structure; understanding the self	Mental ill‐health; disaffected/excluded youth; older people	–	Recovery Model
Iancu et al. (2014)^b^	7	Belonging/nonjudgement; learning skills^a^; meaningfulness; physical health; social relationships; understanding the self	Mental ill‐health	Increased confidence, mood and less tiredness	Recovery Model
Kaley (2015)^b^	22	Achievement and satisfaction; belonging/nonjudgement^a^; creating a new identity; feeling valued/respected; meaningfulness; nurturing; social relationships; stimulation; understanding the self	Learning difficulties	Increased independence, well‐being and reduced anxiety and healthy lifestyle	Therapeutic Landscape Concept
Kogstad et al. (2014)^b^	6	Feeling safe; meaningfulness; physical well‐being; reflection; social relationships; understanding the self	Disaffected/excluded youth	–	Recovery Theory
Leck et al., (2015)	16	Achievement and satisfaction; belonging/nonjudgement; feeling safe; learning skills; meaningfulness; nurturing; physical well‐being; social relationships^a^; structure	Mental ill‐health; substance misuse; disaffected/excluded youth; learning difficulties	Increased happiness and improved prosocial behaviours	ART, Biophilia, PET
Pedersen et al. (2012b)^b^	16	Achievement and satisfaction^a^; belonging/nonjudgement; distraction; feeling safe; feeling valued/respected; learning skills; meaningfulness; nurturing; physical well‐being; stimulation; understanding the self	Mental ill‐health: people with depression	Increased confidence and independence	Biophilia; Self‐efficacy Theory
Schreuder et al. (2014)^b^	5	Feeling valued/respected^a^; reflection; understanding the self^a^	Disaffected/excluded youth	–	Salutogenic Theory
The North Essex Research Network (2013)	4	Achievement and satisfaction; social relationships; stimulation; understanding the self	Mental ill‐health	Increased independence	None stated
De Bruin et al. (2015)	5	Feeling valued/respected; meaningfulness	Older people with dementia	–	None stated
Anderson et al. (2017)	1	Social relationships	Older people with cognitive impairment or clinical depression and younger adults with traumatic brain injury	Increased confidence and independence	None stated
Ellingsen‐Dalskau et al. (2016)	7	Understanding the self; reflection; achievement and satisfaction; belonging/nonjudgement; feeling valued and respected	Mental ill‐health	Feeling happier and having more energy	Self Determination Theory

a Category with most findings.

^b^ studies scoring higher on quality assessment.

^c^ philosophical concept rather than theory.

##### Comparing mechanisms across client groups

Where there were sufficient data, we ordered the categories of mechanism according the frequency with which they were reported for each client group (Table 11).

**Table 11:** Numerical representation of qualitative findings of mechanisms for how care farming might work in different client groups

Category (containing qualitative findings)	Rank* (*n*th of 15 categories) across different client groups**			
	All client groups (*n *= 18 studies/*n *= 177 findings)	MH/SM (*n *= 12 studies/*n *= 118 findings)	DY (*n *= 4 studies/*n *= 37 findings)	LD (*n* = 4 studies/*n *= 59 findings)
Achievement and satisfaction	3	3	11	**3**
**Belonging/nonjudgement**	**2**	**2**	4	**1**
Creating a new identity	13	15 (no findings)	13 (no findings)	7
Distraction	10	11	13 (no findings)	12 (no findings)
Feeling safe	2	5	**3**	6
Feeling valued/respected	4	8	5	5
Learning skills	3	9	8	7
Meaningfulness	2	6	8	4
Nurturing	5	12	12	10
Physical health	2	7	5	11
Reflection	4	14 (no findings)	8	12 (no findings)
**Social relationships**	**5**	**3**	**1**	**2**
Stimulation	5	13	13 (no findings)	7
Structure	4	10	5	7
**Understanding the self**	**1**	**1**	**2**	5

Abbreviations: ASD, autism spectrum disorder; DY, disaffected youth; LD, learning difficulties; MH, mental ill‐health; SM, substance misuse.

* Rank represents the frequency of the findings in each category and the spread of the findings across the studies for that client group.

** Older people and autism spectrum disorder not separately represented due to very low numbers of findings. The most common categories across all studies are highlighted in bold.

As all of the substance misuse findings were reported with mental illness findings, we report these as one client group. As the largest group, with 105 findings from 10 studies, a similar pattern to the overall findings was present in the mental health problems and substance misuse group. No findings relating to “reflection” or “creating a new identity” were found in this combined client group.

For disaffected youth, “feeling safe” was more frequently reported than “belonging and non‐judgement”. “Achievement and satisfaction” was frequently mentioned in both the mental health problems/substance misuse group and the learning disabilities group, but it was reported less often in the disaffected youth group. “Reflection” was also reported more often in the disaffected youth group compared to the others.

In the learning disability client group, “understanding the self” was reported less frequently than “social relationships”, “belonging and non‐judgement”, “social relationships” and “meaningfulness”. “Physical health” was also reported much less frequently in this client group than in the others. “Creating a new identity”, which described how people with learning disabilities aligned themselves with the farmer, was the seventh most often reported category, but did not appear in either the mental illness/substance misuse or the disaffected youth groups.

As there were only 10 and five findings from the older people and autistic spectrum disorder client groups respectively, we did not order the mechanisms according to frequency of reporting.

##### Proximal outcomes

We extracted 24 proximal outcomes (Table 10), identified by participants in the qualitative studies as benefits of being on a care farm. Most (*n *= 11) related to emotions, such as increased confidence and self‐esteem, which mainly arose from studies underpinned by the recovery model for mental health. Improved coping and feelings of well‐being were also mentioned in numerous studies, as was independence. In five studies there were no reported outcomes. There were many more benefits reported by service users than those explicitly proposed by the theories, but as already mentioned in Section 6.3.1 (Stage 1), this may reflect the emphasis on theories on the mechanisms. In a study involving disaffected youth, only two proximal outcomes (happiness and changing behaviours) were reported.

#### 6.3.3 Stage 3: mapping of qualitative data to theoretical framework and creation of logic models

The categories of mechanisms from the qualitative studies were mapped to the five theoretical concepts (Table 12). Some of the categories fit across more than one concept. So, for example, “belonging/non‐judgement” included findings such as “being in an inclusive environment” and “animals are safe and do not judge”. We considered that the former example fitted better with the theoretical concept of “being socially connected” while the latter finding fit with “mental well‐being” (Table 13).

**Table 12:** Mechanisms mapped to theoretical concepts

Theoretical concept	Categories of mechanisms
Restorative effects of nature	
Being socially connected	Belonging/nonjudgement, feeling valued and respected, social relationships, feeling safe and nurturing
Personal growth	Learning skills, understanding the self, reflection, nurturing, achievement and satisfaction, meaningfulness and creating a new identity
Physical well‐being	Physical well‐being
Mental well‐being	Feeling safe, structure, belonging/nonjudgement, meaningfulness, reflection, feeling valued and respected, achievement and satisfaction, stimulation and distraction

**Table 13:** Representation of theoretical concepts in categories of mechanisms reported in qualitative studies

Theoretical concept	Number of qualitative mechanism findings			
	All client groups	MH/SM	Disaffected youth	Learning disabilities
Restorative effects of nature	0	0	0	0
Being socially connected	43	28	12	18
Personal growth	44	25	9	13
Physical well‐being	10	9	3	2
Mental well‐being	80	52	13	25
All	177	118	37	58

Abbreviations: MH, mental ill‐health; SM, substance misuse.

Only four single findings within the mechanism categories of “reflection”, “stimulation” and “feeling safe” appeared to map to the theoretical concept of “restorative effects of nature”. These findings were “silence in nature”, “peace”, “enjoying the sensory experience of being with animals” and “cuddling the animals gives a sense of security”. We considered that these primarily mapped to the theoretical concept of “mental well‐being”, but had links to the “restorative effects of nature”. The dearth of findings that map to this theoretical concept occurred despite “the setting” of a farm, which could be considered as “nature”, being mentioned frequently in the qualitative studies as an important component of the intervention. The theoretical concepts of “mental well‐being”, “being socially connected” and “personal growth” were best represented by the qualitative mechanisms overall. Across the three main client groups (mental health problems/substance misuse; disaffected youth; learning difficulties), there were some differences. In the mental health problems/substance misuse group, the number of mechanism findings that mapped to “mental well‐being” was almost double that of any other theoretical concept. In the other client groups, “being socially connected” and “mental well‐being” were similarly represented by the mechanisms. The categories of mechanisms were then combined with the intervention components and proximal outcomes to create a logic model for the following client groups:

- All client groups (Figure 4)
- Mental health problems and substance misuse (Figure 5)
- Disaffected youth (but includes some qualitative findings from other client groups) (Figure 6)
- Learning difficulties (but, as above, includes some qualitative findings from other client groups) (Figure 7)

In general, there was a lack of sufficient evidence detailing which intervention component linked to which categories of mechanisms, and thereafter which proximal outcomes and outcomes. Therefore, the logic models only provide a single connecting arrow between each of these aspects.

#### 6.3.4 Quantitative results

The quantitative evidence was mapped onto both the proximal outcomes and the endpoint health outcomes in the logic models to. Based on our overall logic model built from theory and the qualitative evidence, we expected to find empirical evidence suggesting that care farms would improve:

Endpoint outcomes:

- Quality of life (primary outcome identified from theory)
- Anxiety (primary outcome identified from theory and qualitative studies)
- Depression (primary outcome identified from theory)

Proximal outcomes:

- Self‐efficacy (theory)
- Confidence (theory and qualitative studies)
- Coping skills (theory and qualitative studies)
- Independence (qualitative studies)
- Social activity (qualitative studies)
- Self‐esteem (qualitative studies)
- Self‐image^2^ (theory and qualitative studies)
- Physical well‐being (including having more active lifestyles and being physically tired (all from qualitative studies)
- Happiness or well‐being (qualitative studies)
- Vocational skills (qualitative studies)
- Stress (theory)
- Negative behaviours (theory and qualitative studies)
- Medication usage (qualitative studies)

No quantitative studies were found that evaluated the impact of care farms on confidence, personal identity and physical well‐being (including tiredness). Changes in negative social behaviours were measured, but only one form (reduction in reoffending) was clearly defined. Additionally, vocational skills may have been measured in the form of occupational functioning and work abilities. However, as these outcomes were either not defined or incorporated highly subjective measurements, there is no clear result.

We found evidence relating to quality of life, self‐efficacy, coping skills, independence, social activity, well‐being, anxiety, depression, stress and medication usage. In addition to the outcomes identified from theory and qualitative evidence in the logic model, four further outcomes were found from the quantitative studies, namely cognitive functioning, improvements in psychiatric status (from chronic psychiatric illness), positive affect and appetite and eating pattern. These were added to the logic models.

The majority of the evidence was derived from studies involving service users with mental health problems and substance misuse problems. This meant that quantitative results relating to disaffected youth and users with learning difficulties could not be mapped against these logic models.

Given the heterogeneity of the studies in terms of study design, participant groups, outcome measures, synthesis of quantitative results through meta‐analysis was not appropriate. Quantitative endpoint and proximal outcomes are provided in Tables 14 and 15.

**Table 14:** Results of primary outcomes

Outcomes	Instrument	Reference	Study design and sample size	Target group	Baseline mean (*SD*)	First follow‐up mean (*SD*)	Second follow up mean (*SD*)	Result as reported by authors	Effect size [95% CI]*	Summary of effects
Depression	The Beck Depression Inventory	Berget et al. (2011, 2008)	RCT CF = 60 (41 at first follow‐up); control = 30 (28 at first follow‐up)	Mental ill‐health	CF: 20.4 (1.74) CO: 18.9 (1.79)	CF: 17.9 (1.82) CO: 16.9 (2.16)	CF: 15.3 (1.84) CO: 14.4 (1.86)	There was no significant change in anxiety between groups	First follow‐up: *d *= 0.04 [−0.44, 0.52]; second follow‐up: *d *= 0.1 [−0.38, 0.58]	The results were not statistically significant
	Beck Depression Inventory	Pedersen et al. (2012b)	RCT CF = 16 CO:13	Mental ill‐health	CF: 23.9 (9.3) CO: 34.2 (8.8)	CF: 17.3 (12.6) CO: 28.2 (11.0)	CF: 17.8 (12.0) CO: 27.3 (13.0)	There was no significant group difference at any of the follow‐ups (*F* (2,9/80,9) = 0.66, *p* = .58)	*d *= 0.3 [−0.43, 1.04]	The results were not statistically significant
	Beck Depression Inventory	Gonzalez et al. (2011a)	UBA *N *= 46	Mental ill‐health	Cohort 1: 27.3 (6.8) Cohort 2: 24.1 (8.4)	Cohort 1: 17.6 (7.4) Cohort 2: 19.6 (8.0)	Cohort 1: 20.8 (9.0) Cohort 2: 20.4 (10.3)	Positive significant difference in depression in both cohorts at first and second follow‐up (*f *= 20.94, *p *= .001; *f *= 13.76 *p *= .001)	–	–
	The Profile of Mood State Questionnaire	Hine et al. (2009, 2008b)	UBA *N *= 72	Mental ill‐health	41.71 (5.12)	39.45 (3.89)	–	There was a positive significant difference in the depression scores (*t* (50) = 4.50, *p *< .001)	–	–
Anxiety	The Spielberger State‐Trait Anxiety Inventory	Berget et al. (2011, 2008)	RCT CF = 60 (41 at first follow‐up); control = 30 (28 at first follow‐up)	Mental ill‐health	CF: 51.2 (2.07) CO: 44.8 (2.10)	CF: 49.30 (2.12) CO: 45.7 (2.06)	CF: 44.6 (2.08) CO: 46.7 (2.76)	No significant change in anxiety between groups at first follow‐up but they found a statically significant positive effect at second follow‐up	First follow‐up *d *= 0.02 [−0.46, 0.5]; second follow‐up: *d *= 0.51 [0.23,1]	The results were not statistically significant at first follow‐up. However, at the second follow‐up there was a statistically significant medium size positive effect
	The Spielberger State‐Trait Anxiety Inventory‐State Subscale	Pedersen et al. (2012b)	RCT CF = 16 CO:13	Mental ill‐health	CF: 55.2 (8.7) CO: 62.3 (7.5)	CF: 49.4 (13.9) CO: 55.5 (13.1)	CF: 48.5 (12.4) CO: 56.5 (14.3)	There was no significant group difference at any of the follow‐ups (*F* (1,9/52,4) = 0.12, *p* = .88)	*d *= 0.12 [−0.6, 0.86]	The results were not statistically significant
	The State‐Trait Anxiety Inventory‐State Subscale	Gonzalez et al. (2011b)	UBA *N *= 46	Mental ill‐health	Cohort 1: 56.8 (8.8) Cohort 2: 55.4 (11.4)	Cohort 1: 49.3 (9.4) Cohort 2: 52.7 (9.2)	Cohort 1: 53.1 (10.4) Cohort 2: 52.7 (11.4)	In both cohorts there was a positive significant difference at first follow‐up (*f *= 9.49, *p *= .004), but not significant at second follow‐up (*f *= 2.82, *p *= .101)	–	–
QOL	Norwegian version of Quality of Life Scale	Berget et al. (2011, 2008)	RCT CF = 60 (41 at first follow‐up); control = 30 (28 at first follow‐up)	Mental ill‐health	CF: 64.3 (14.93) CO: 63.2 (14.06)	CF: 64.3 (17.09) CO: 64.4 (13.52)	CF: 66.7 (16.86) CO: 66.0 (15.25)	No significant change in quality of life between groups at both follow‐ups	First follow‐up: *d *= 0.17 [−0.31, 0.65]; second follow‐up: *d *= 0.15 [−0.33, 0.63]	The results were not statistically significant
	WHOQOL‐brief	Elings et al. (2011)	CBA *N *= 113	Mental ill‐health	Not provided	Not provided	Not provided	Authors reported no significant change in quality of life between groups, at follow‐ups	Insufficient information to calculate effect sizes	–
	CORE‐OM	Hine et al. (2009)	UBA *N *= 20	Mental ill‐health	Not reported	Not reported	–	Authors report that there were no differences in mean scores between before and after the intervention	–	–
	EQ‐5D (VAS)	Lambert (2014)	UBA *N* = 83	Other client groups	52.85	69.93	–	There was a 17.08 points improvement in quality of life from baseline to end of the intervention for the mixed client group	–	–

Abbreviation: CBA, controlled before and after study; CF, care farm; QOL, Quality of Life; RCT, randomised controlled trial; UBA, uncontrolled before and after study; VAS, Visual Analogue Scale.

**Table 15:** Results of proximal outcomes

Outcomes	Instrument and definition	Reference	Study design and sample size	Target group	Baseline mean (*SD*)	First follow‐up (posttest) mean (*SD*)	Second follow up mean (*SD*)	Result as reported by authors	Effect size [95% CI]^a^	Summary of effects
*Mental health outcomes*										
Self‐efficacy	The Generalised Self‐Efficacy Scale: assess an individual's optimistic self‐beliefs to respond to difficult situations in life	Pedersen et al. (2012b)	RCT *N *= 29 (CF = 1; CO:13)	Mental ill‐health	CF: 23.0 (4.9) CO: 18.9 (6.4)	CF: 25.6 (6.7) CO: 21.5 (6.6)	CF: 26.1 (6.9) CO: 21.5 (8.3)	There was no significant group difference at any of the follow‐ups (*F* (3,2/86,0) = 0.38, *p* = .78)	*d *= 0.23 [−0.5, 0.96]	The results were not statistically significant
		Berget et al. (2008)	RCT CF = 60 (41 at first follow‐up); control = 30 (28 at first follow‐up)	Mental ill‐health	CF: 23.1 (5.12) CO: 25.6 (6.40)	CF: 23.5 (6.56) CO: 25.3 (6.62)	CF: 25.7 (5.93) CO: 25.4 (5.92)	Statistically significant positive improvement from baseline to second follow‐up in the intervention group (MD = 2.6, *t* = 3.68, *p *= .001)	First follow‐up: *d *= 0.35 [−0.45, 0.52]; second follow‐up: *d *= 0.5 [0.02, 0.99]	The results were not statistically significant at first follow‐up, but at second follow‐up there was a significant effect
Self‐esteem	Rosenberg Self Esteem Scale: measures a person's self‐worth by assessing positive and negative feelings about the self	Hine et al. (2009, 2008b)	UBA *N *= 72	Mental ill‐health; drug and alcohol problems; older people; offenders	21.47 (5.80)	19.65 (6.43)	–	Mean difference was 1.82 points (*p *< .01)	–	–
		Hassink et al. (2011)	UBA *N *= 48	Disaffected/excluded youth	28.9 (5.8)	33.5 (5)	–	Statistically significant positive effect on self‐esteem (MD = 4.5, *p *< .001)	–	–
Stress	The Perceived Stress Scale: the degree to which situations in one's life are appraised as stressful	Gonzalez et al. (2011b)	UBA *N *= 46	Mental ill‐health	14.1 (2.3)	13.0 (2.3)	13.3 (2.4)	Statistically significant reduction in stress at first follow‐up (MD = 1.1, *p *= .003) but this was not maintained at second follow‐up (MD = 0.8, *p *= .063)	–	–
Coping	Coping Strategies Scale: measured control and planning ability in daily life (control coping) and also coping by means of social support	Berget et al. (2008)	RCT CF = 60 (41 at first follow‐up); control = 30 (28 at first follow‐up)	Mental ill‐health	CF: 31.6 (8.51) CO: 32.2 (7.38)	CF: 32.8 (8.67) CO: 31.4 (S8.69)	CF: 34.3 (8.10) CO: 31.6 (8.02)	ANOVA analysis revealed no treatment effect for any of the follow‐up periods (*f* = 0.79, *p *> .05)	First follow‐up: *d *= 0.16 [–0.32, 0.64]; second follow‐up: *d *= 0.33 [–0.15, 0.82]	The results were not statistically significant
Mood	The Profile of Mood State Questionnaire: measured anger, confusion, depression, fatigue, tension and vigour (the lower the score, the better the overall mood)	Hine et al. (2009, 2008b)	UBA *N *= 72	Mental health; drug and alcohol problems; older people; offenders	165.47 (36.40)	147.04, (25.94)	–	Author reported a highly statistically significant improvement in participants’ mood (*t* (50) = 6.30, *p *< .001)	–	–
Mental status	Brief Psychiatric Rating Scale: this instrument is used to assess psychotic disorders, especially schizophrenia. Decreasing effects suggests that the participants symptoms are improving	Javed et al. (1993)	UBA *N *= 25	Mental ill‐health	70 (9.35)	Yr 1 = 55 (4.6)	Yr 2 = 43 (5.3) Yr 3 = 33.18 (8.13)	Significant improvement (*MD *= 36.82, *p *< .01)	–	–
Mental functioning	Mental Health Inventory: measured a person's mental status including anxiety, depression, behavioural control, positive effect and general distress	Elings et al. (2011)	CBA *N *= 113	Mental ill‐health/drug and alcohol problems	19.5 (5.6)	20.5 (5.3)	–	Mental functioning improved slightly (MD = 1), however it was not statistically significant	Insufficient information to calculate effect sizes	–
Positive affect	Positive and Negative Affect Scale: the extent to which participants currently experienced the following affects: interested, enthusiastic, inspired, proud, alert, strong and active	Gonzalez et al. (2011a)	UBA *N *= 46	Mental ill‐health	2.25 (0.82)	2.51 (0.79)	2.36 (0.89)	Statistically significant improvement at first follow‐up (MD = 1.1, *p *= .024) but this was not maintained at second follow‐up (MD = 0.8, *p *= .225)	–	–
Cognitive functioning	Mini Mental State Examination: measures a person's mental impairment including memory, attention and language	de Bruin (2009)	CBA *N *= 88	Older people >65 with dementia	CF: 19.4 (male) 19 (female) Co: 20(male) 18.2 (female)	Not provided	–	Authors state that there was no significant change in cognitive functioning at 6‐month follow‐up	Insufficient information to calculate effect sizes	–
Self‐determination	IPC LOC Scale (internal locus)	Hassink et al. (2011)	UBA *N *= 45	Disaffected youth	38.6 (6.2)	37.0 (7.6)	–	There was no statistically significant difference (MD = −0.22)	–	–
*Social outcomes*										
Social functioning	The Social Functioning Scale: measured social engagement, interpersonal communication, independence and competence	Elings et al. (2011)	CBA *N *= 113	Mental ill‐health/drug and alcohol problems	Not provided	Not provided	–	Authors report that there was no effect on social functioning between the two groups	Insufficient information to calculate effect sizes	–
Group cohesion	The Therapeutic Factors Inventory Cohesiveness Scale: measured a person's sense of belonging to the group and experience of acceptance, trust, and group cooperation	Gonzalez et al. (2011a, 2011b)	UBA *N *= 46	Mental ill‐health	5.66 (0.97)	5.89 (0.96)	–	Authors found that group cohesion improved (*F* = 3.21, *p* = .054)	–	–
Reoffending	The number of new convictions	Marshall and Wakeham (2015)	UBA *N *= 10	Offenders	–	–	–	65% reduction in offending	–	–
Problem behaviour	Internalising problems, anxiety/depression, reserved, externalising problems and delinquent behaviour	Hassink et al. (2011)	UBA *N *= 45	Disaffected youth	62.2 (10.3)	52.2 (8.7)	–	The authors reported significant, positive effect on problem behaviours at 6‐month follow‐up (MD = 1.05, *p *< .001)	–	–
*Physical outcomes*										
Functional performance	The Barthel Index: an individual's dependence on a caregiver	de Bruin et al. (2012)	CBA *N *= 88	Older people >65 with dementia	Change over 6 months—MD Cohort 1: CF: 6.4 (11.5) CO: 0.8 (6.8); Cohort 2: CF: 3 (6.7) CO: 0 (5.7) Cohort 3: CF: 2.2 (7.7) CO: 1.7 (3.2)	–	–	The authors reported no significant change in functional performance between groups	Insufficient information to calculate effect sizes	–
Appetite and eating pattern	Simplified Nutritional Appetite Questionnaire: measures an individual's dietary intake and predicts weight loss	Elings et al. (2011)	CBA *N *= 113	Mental ill‐health/drug and alcohol problems	Not provided	Not provided	–	Authors report that no differences in appetite and eating patterns between groups	Insufficient information to calculate effect sizes	–
Medication usage	Provided by the Central Indication Committee for Care	de Bruin et al. (2012)	CBA *N *= 88	Older people >65 with dementia	Change over 6 months—MD Cohort 1 CF: 0.2 (0.8); CO 0.5 (0.8) Cohort 2 CF 0.8 (1.4) CO 0.3 (2.0) Cohort 3 CF: 0.1 (1.0) CO: 0.5 (0.8)	–	–	The authors reported no significant change in medication use	Insufficient information to calculate effect sizes	–
Rehabilitation	Morningside Rehabilitation Status Scale: assess the functioning of a person, including: independence/dependence, activity/inactivity, social integration/isolation and effect of current symptoms on lifestyles	Javed et al. (1993) CBA‐check	UBA *N *= 25	Mental ill‐health	22.51 (*SD*, 3.01)	Yr 1 = 19.3 (1.8)	Yr 2 = 15 (2.3) Yr 3 = 11.37 (2.47)	The authors reported a statistically significant improvement in rehabilitation	–	–

Abbreviations: ANOVA, analysis of variance; CBA, controlled before and after study; CF, care farm; IPC LOC, Internal Powerful others and Chance Locus of Control Scale; QOL, Quality of Life; RCT, randomised controlled trial; UBA, uncontrolled before and after study; VAS, Visual Analogue Scale.

^a^ Effect sizes were calculated using aggregate data provided in the original article when possible.

#### 6.3.5 Stage 4: mapping quantitative outcomes to the logic models

##### Mapping outcomes to the mental health/substance misuse logic model

###### Primary outcomes

Three studies evaluated the effectiveness of care farms on quality of life, for service users with mental health or substance misuse problems. Berget et al. (2008) reported no significant change in quality of life between groups, at 12 week and 6‐month follow up. Similarly, Elings et al. (2011) also found no significant change in quality of life between groups, at 6‐ and 12‐month follow up. Furthermore, Hine et al. 2009 reported a positive change in mean scores between baseline and end of intervention (approximately 10 weeks), but this was not statistically significant.

Three studies (two RCTs and a UBA study) assessed participants’ anxiety at two follow‐up points (see Table 2). The first RCT found no significant change in anxiety between groups at 12 week follow‐up (Berget et al., 2011). However, at 6‐month follow‐up, they found a statistically significant positive effect of the intervention in reducing anxiety compared to the control group. The authors reported that this positive effect is also clinically significant because the participants were diagnosed with severe anxiety at baseline, which improved to moderate anxiety at 6‐month follow‐up. In the second RCT, Pedersen et al. (2012b) found no significant change in anxiety between groups at the end of the intervention follow‐up (12 weeks) and 3 months after the intervention (Pedersen et al., 2012b). Gonzalez et al. (2011a, 2011b) reported a statistically significant but transient reduction in anxiety at 12 week follow‐up, but anxiety levels were still within the clinically severe range (remaining above the estimated clinical cut‐off of ≥45) (Spielberger, 1983). At 3‐month follow‐up, change in anxiety scores were no longer statistically significant.

Four studies reported depression outcomes immediately after completion of the intervention. Both RCTs reported no significant change in depression between groups at 12 week follow‐up (Berget et al., 2011; Pedersen et al., 2012b). A UBA study found a statistically significant reduction in depression at the end of the intervention (12 weeks), and 3 months after the intervention (Gonzalez et al., 2011a, 2011b). The results were clinically significant as the participants BDI scores moved from moderate to mild depression between baseline and first follow‐up. However, the results at second follow‐up were no longer clinically significant as the participants returned to baseline moderate level (Beck, Steer, & Brown, 1996). In a further UBA study (Hine, 2008), a statistically significant decrease in the depression scores of participants from the start to the end of the intervention was reported; however, no further follow‐ups were reported.

Overall, the studies did not indicate that care farms can improve quality of life for people with mental health problems. Also, the evidence on the effectiveness of care farms to reduce anxiety and depression within mentally unwell service users and those with substance misuse problems is inconsistent and therefore inconclusive.

###### Proximal outcomes

Two RCTs measured self‐efficacy and both found no significant change in self‐efficacy, between groups, at 12 week follow‐up (Berget et al., 2008; Pedersen et al., 2012b). However, at 6‐month follow‐up, Berget et al. (2008) found a statistically significant improvement in self‐efficacy.

Self‐esteem was measured in one UBA studie (Hine et al., 2008b) the authors claim a statistically significant improvement in self‐esteem at the end of the intervention, with no further follow‐ups reported. A statistically significant reduction in stress was also found at the end of the intervention (12 weeks); however, this effect was not maintained 3 months after the intervention (Gonzalez et al., 2011a, 2011b). In addition, Berget et al. (2008) reported no significant effect on coping, compared to the control group, at 12 week and 6‐month follow‐up.

Hine et al. (2008b) reported a statistically significant improvement in mood (i.e., anger, confusion, depression, fatigue, tension and vigour) at the end of the intervention. Similarly, Javed et al. (1993) reported a statistically significant improvement in mental status and rehabilitation among service users with schizophrenia, at three‐year follow‐up.

Additionally, Gonzalez et al. (2011a, 2011b) measured positive affect, which is the extent to which participants experienced the following affects: interested, strong, enthusiastic, inspired, proud, alert, strong and active. At 12 week follow‐up, there was a statistically significant improvement in positive affect, but this was not maintained 3 months after the intervention.

Social outcomes were measured in two studies. Social functioning (including social engagement, interpersonal communication, independence and competence) was measured in one CBA study and at 12‐month follow‐up, there was no effect on social functioning between the participants that went to care farms compared to participants that attended day activity projects (Elings et al., 2011). Gonzalez et al. (2011a, 2011b) assessed participants’ group cohesion using the Therapeutic Factors Inventory Cohesiveness Scale which captured a person's sense of belonging to the group and experience of acceptance, trust, and group cooperation. During the length of the intervention (12 weeks), they found that the participants’ group cohesion significantly improved.

One study measured participants’ appetite and eating patterns and at 12‐month follow‐up, found no differences in appetite and eating patterns between service users attending care farms versus those at day activity projects (Elings et al., 2011).

Overall, across all secondary outcomes there is inconsistency in the findings at immediate, 3 months, 6 months and longer‐term follow‐ups. Most studies measured immediate follow‐up with few addressing longer‐term impacts. The impact of care farms on psychological, social and physical outcomes in service users with mental health problems or substance misuse problems remains unclear.

##### Mapping outcomes to the disaffected youth logic model

Three outcomes were reported for disaffected youth both at 6‐ and 12‐month follow‐ups (Hassink et al., 2011). The authors reported a significant positive effect (MD = 1.05) on problem behaviours (i.e., internalising problems, anxiety/depression, being reserved, externalising problems, and delinquent behaviour) at 6‐month follow‐up. Four of seven aspects of coping questionnaire showed significant, positive improvements, including: seeking social support, passive expectancy, self‐esteem and active problem solving. No difference was found in self‐determination at both follow‐ups. The evidence on the impact of care farms for disaffected youth is scant.

##### Evidence for other client groups/mixed groups

Lambert (2014) observed a 17.08 points improvement in quality of life as measured by the EQ‐5D health state score from baseline to end of the intervention for the mixed client group. However, the author did not report whether this overall score is statistically significant, or provide a standard deviation. Nevertheless, Lambert (2014) conducted subgroup analyses and found statistically significant improvement in quality of life among people with anxiety or depression, personality or social issues, and psychosis, but not for people with learning difficulties.

In a CBA study involving older people, de Bruin (2009) reported no significant change in cognitive functioning at 6‐month follow‐up between those attending care farms compared to a control group that attended day care facilities.

In a very small UBA study, Marshall and Wakeham (2015) reported a 65% reduction in expected 12‐month reoffending rates for offenders attending a CF as part of their community order.

De Bruin et al. (2012) assessed whether older peoples’ functional performance (an individual's dependence on a caregiver) and medication use would change after attending the care farm. At 6‐month follow‐up, the authors reported no significant change in functional performance and medication use, compared to a control group that attended day care facilities.

Evidence on the impact of care farms for other client groups was scant. No conclusions could be drawn from the evidence that was available.

## 7 DISCUSSION

### 7.1 Summary of main results

The studies included approximately 980 participants from a range of client groups. The largest single client group (albeit spanning a range of conditions within the group) was those with mental health problems (12 of 31 studies).

Based on data from 18 qualitative studies and information from 15 theories, we were able to develop logic models to describe potential mechanisms for change for four client groups, namely those with mental health and/or substance misuse problems,^3^ disaffected youth, and people with learning disabilities. While there were some data on older people and individuals with autistic spectrum disorder it was insufficient to develop a client specific logic model. The developed overall model (for all client groups) highlights the importance of being in a nonjudgmental, structured, stimulating and safe environment that allows for reflection, thus helping individuals to: understand themselves; feel that they belong, are valued and respected; develop social relationships; have a sense of achievement, satisfaction and meaningfulness; learn new skills; allow for the development of and nurture a new identity if wanted; and become physically healthy. These mechanisms are a good fit with a number of theories, and this review provides the first attempt to map evidence from quantitative and qualitative studies against the concepts of these theories in relation to care farms.

Although we ordered mechanisms based on frequency and spread, we do not suggest that any one mechanism is any more important than any other at an individual level. However, based on available data, we observed potential differences in the way care farms work for particular client groups. While this may reflect differences in the focus of the topics covered in the qualitative methods used by different authors, these differences are worth further exploration. For example, a sense of achievement and satisfaction appeared to be more important to the substance misuse/mental illness and the learning disabilities service users groups compared to the disaffected youth service group, where feeling safe may be a priority. In this latter client group, having the opportunity to reflect seemed to be valued. While we do not have sufficient data to be able to robustly link the intervention components to the mechanisms, we do tentatively suggest that in the disaffected youth group the emphasis on reflection appears to fit with the greater focus on the “setting” aspect of the intervention. As with the causal pathway between intervention components and mechanisms, the relationship between many of the mechanisms and proximal outcomes/outcomes is unclear. For example, “understanding the self” (a mechanism category), which included findings such as increasing self‐respect and understanding of tasks that are manageable, could potentially be linked to proximal outcomes relating to self‐efficacy and improved confidence. However, with others which were seemingly important mechanisms such as “belonging and non‐judgement”, the connection to outcomes is less clear. It is likely that many of these mechanisms interact in a way that is not yet understood to influence outcomes. These hidden features of complex interventions are commonly observed within logic models.

A key finding within this aspect of the review was that the theoretical concept “restorative effects of nature” was represented by the intervention components (but to a notably lesser extent than “the work” and “the farmer” components), but was not represented at all in the categories of mechanisms. This was somewhat surprising given that, informally at least, one of the most lauded attributes of care farming is its nature‐based approach. Only four findings of the 164 that mapped to the theoretical concept about mental well‐being could potentially relate to nature. We suggest that the absence or near absence of “the restorative effects of nature” is not a true absence; rather, nature is the essential platform which allows other more overt mechanisms to be acted out. Thus, as individuals recall their experiences on the farm, it is primarily the mechanisms promoted through the work and the interactions with the farmer that are at the forefront. It is not clear exactly what individuals were asked about in the qualitative studies, but given that the aims were primarily about exploring the experience and benefits of care farming, more specific questioning about nature may not have been part of the topic guides.

Despite being able to develop the logic model for the disabilities client group, the lack of quantitative studies with this group meant that we could not map outcome data to the model. While more quantitative data was available for the substance misuse/mental illness groups and the disaffected youth logic models, very limited mapping of secondary outcomes was possible with the latter group. Based on limited quantitative evidence from only two small RCTs, we did not find sufficient evidence to conclude any significant positive effects of care farms in improving quality of life. We did find some limited and inconclusive evidence to suggest that care farming can reduce anxiety. For depression, while there appeared to be significant reductions following the intervention as assessed in UBA studies, the RCT found no significant differences between intervention and control groups, however the small sample size may have undermined the power of this study to detect a difference.

For proximal/secondary outcomes, there were no significant positive effects for self‐efficacy and coping (measured in the RCTs) at the end of the intervention. However, a significant improvement in self efficacy (but not coping) was reported at follow‐up. The possibility that there may be some delayed benefits (as with anxiety) for self‐efficacy requires confirmation by future studies. A number of UBAs reported significant improvements in self‐esteem, stress, affect, mood and group cohesion at the end of the intervention. However, only stress and affect were measured at follow‐up (3 months after the intervention ended), and improvements were not sustained. Most of the primary and secondary or proximal outcomes were limited to immediately postintervention, with only three (social functioning, eating and appetite, and mental status) reported beyond 6 months. With respect to disaffected youth, there was some suggestion that coping might be improved, but no impact identified on self‐esteem.

### 7.2 Overall completeness and applicability of evidence

Most of the studies were conducted within three European countries, in particular in the Netherlands (*n *= 12). This was followed by Norway (*n *= 9) and then the UK (*n *= 7), with two studies in the United States and one study in Pakistan. We know that other countries are active in care farming, particularly Italy, Germany, Denmark, Spain, Sweden, and France, but it would appear that studies measuring health outcomes or exploring health aspects qualitative have not as yet been published in the academic or grey literate. Important demographic information was missing from many of the studies so we cannot comment on the applicability of the evidence across, for example, different ethnic or socioeconomic groups. Most of the studies reported sex disaggregated data. This highlighted that almost double the number of males participated compared to females. It is likely that this reflects what is seen in practice, given that the age ranges of people and the range of client groups in the studies were similar to that seen in practice in the UK at least (Bragg et al., 2014). Most of the studies focused on care farming for mental health problems, with fewer for disaffected youth and older people, and no quantitative studies for people with learning disabilities/autistic spectrum disorder. In the UK, more care farms support people with learning disabilities/ASD than any other client group. Within studies that explored the effectiveness of care farms for people with mental health problems, there was a range of conditions (including anxiety, depression, personality disorder, schizophrenia), and because of small sample sizes it was not possible to say which conditions within this realm would derive most benefit from care farming interventions. Generally in the UK, care farming is for individuals with mild to moderate depression with only a minority specialising in more severe conditions. With respect to this client group, therefore, the research may not reflect usual practice.

One of our aims was to create logic models to describe how care farms may work for different client groups. This was only possible for the mental health problems and/or substance misuse group, disaffected youth and, to a lesser extent, people with learning disabilities. The identified theories included those that attempted to integrate care farming with a particular condition or issue such as the recovery model for mental health problems and the SHIFT model for offending. However, there were other theories that discussed, for example, nature or social support without reference to issues that arise in particular client groups. Having a more integrated theory provided more guidance on expected (proximal and endpoint) outcomes which when combined with outcomes derived from the qualitative literature allowed for a more comprehensive logic model. This was the case with the mental health problems logic model.

While there was a reasonable body of qualitative evidence relating to mechanisms for disaffected youth, findings on proximal or endpoint outcomes were very limited, with only two found. Only one theory (attachment theory) (Bowlby, 1969) was specifically mentioned in relation to adolescents and applied within an excluded overview about animal assisted therapy (Geist, 2011). This did suggest a theoretical relationship between early years parental attachment and socioemotional and behavioural outcomes, but it is not clear if this theory fits better with the behavioural disturbances to disaffected youth (i.e., a delinquency type behaviour) or to a more emotional disorder.

Overall there was little quantitative evidence so our testing of the logic models was limited to mapping quantitative results, as presented in the papers, to the identified outcome measures. This is partly due to the fact that in all quantitative studies with mixed client groups, outcomes were not reported separately. This meant that only limited information on client groups other than those with mental illness was available to be mapped to the logic models. Of particular note was the lack of RCTs, particularly any well‐designed and appropriately powered. This is unsurprising—the third sector, in which care farming resides, presents many methodological and logistical challenges to carrying out this type of research. It may be that natural experiments may prove a valuable design in this context, however, no such studies were found in this review. Even CBAs, which are less demanding in terms of resources and methods, but not as rigorous as RCTs, were few in number. Thus, much of mapping of outcomes relied on highly biased uncontrolled studies.

### 7.3 Quality of the evidence

#### 7.3.1 Qualitative studies

More than half of the qualitative studies met <50% of the quality assessment criteria and only two met more than 60% of the criteria. Studies performed well in relation to clarity about the area of study, number of interviews performed, and the provision of clear themes and quotes supporting their findings. However, areas that were poorly addressed include the provision of details about relationships between the researcher and the interviewees. Although this type of research involves very vulnerable client groups, only one study demonstrated evidence that they had sought to embed themselves in the setting prior to data collection, to foster a trusting relationship that would facilitate a more in‐depth research data collection. Likewise, standard good practice of obtaining informed consent and ethical approvals was only reported in six studies. Although not specifically a quality criterion, we observed a clear connection between study quality and the use of contextual theories to guide the research question and analysis. Those that used a theory much more often met more of the quality criteria. Again, provision of basic demographic data (age and gender), which was also not a specific quality criterion, was often absent in studies. Six of the qualitative studies were not published in academic journals and missed the opportunity for rigorous external peer‐reviewing. Some were locally commissioned without the intention of publishing in a journal and this may explain the lack of good quality reporting.

In the qualitative studies, the vast majority of themes did not separate the experiences of different client groups.

#### 7.3.2 Quantitative studies

There was much heterogeneity across the studies in terms of the client groups, duration and intensity of the intervention, outcomes and outcome tools, periods of follow‐up and overarching study design; hence, we were unable to conduct a meta‐analysis.

Heterogeneity was also observed in the outcomes and measures applied in the quantitative studies. Twenty three different outcomes were measured over 12 studies, probably reflecting the range of client groups and the varied way in which care farms might be considered to impact on lives. Quality was also compromised by the use of unvalidated outcomes within a number of studies. The majority of quantitative studies in general did not offer a theoretical basis or even suggest a mechanism by which the intervention might work, questioning the basis of decisions on types of outcomes.

Most of the quantitative evidence was derived from UBA studies, which do not control for threats to internal validity and thus causal inferences cannot be made from these studies. Furthermore, most of the outcome data were restricted to immediately after the intervention, potentially offering inadequate latency for observed effects. Only three outcomes were reported at 6 months, and a further three for 12 months and beyond.

All of the quantitative studies had a high risk of bias. In the two RCTs, three and four of the seven quality assessment domains were unclear. Studies did not demonstrate any evidence of bias in the selection of outcomes reported, and all data on attrition was reported. However, neither study blinded outcome measurement, and one of the two studies lacked clarity about potential contamination between the groups and about differences in baseline characteristics. Furthermore, in one study the differences in baseline outcomes were not adjusted for in the analysis.

Similarly all other CBA and UBA studies were found to be at high risk of bias. In particular, only one study reported data on attrition. As with the qualitative studies, six (one CBA and five UBA) of the 13 quantitative studies (including the mixed methods study in the total) were reports that were not published in a peer‐reviewed journal, and therefore were not subjected to the rigors of an external review processes. In general, samples sizes across most of the studies were small and so were likely underpowered, thus increasing the risk of type II error.

### 7.4 Limitations and potential biases in the review process

We used a comprehensive search strategy, which we believe identified all published studies of care farms. We supplemented our electronic search by asking research collaborators across Europe to identify relevant networks, other colleagues and websites for unpublished reports. In addition, Care Farming UK emailed all care farms in the UK for any unpublished reports. We found one PhD thesis and subsequently found the published paper relating to this. We found 126 articles via our grey literature retrieval methods. This was not unexpected—care farms often fall within the third sector, so we anticipated that many evaluations would be conducted for the purposes of obtaining funding, and therefore would remain unpublished. We used multiple reviewers and rigorous approaches during all key stages of the review.

With respect to testing our model for the disaffected youth group, we observed that the measured outcomes did not necessarily reflect the supporting theory or the qualitative evidence. One way to address this might have been to extract information from the introduction of the studies to identify expected outcomes, but this was not an anticipated finding, and therefore was not built into the methods. Likewise, in reviewing the identified theories we only explored those theories that had been mentioned in connection with care farming. The main aim of the review was to look at the effectiveness of care farming for improving quality of life, and secondarily to understand how care farms might work for different client groups. A more detailed critique of the theories of change for each individual client group and an understanding of how they could inform care farming would fit with a more realist approach.

Although we ordered the categories of mechanisms according to frequency and spread of findings across the studies for each of the different client groups, we recognise that this does not necessarily represent levels of importance for individuals. Furthermore, it is possible that with more interview data or the use of different theoretical frameworks to inform the qualitative research, the order of mechanisms might change. We would suggest, however, that even across studies that used different theoretical frameworks, the same types of findings were reported, suggesting that despite a researcher's agenda, service users still pursue issues that are important to them individually. The categorisation of qualitative mechanisms might be open to bias; however, we mitigated this possibility by using multiple reviewers and conducting several iterations, checking back to the papers to ensure that categorisation remained true to the original meaning and context of the finding. Additionally, during the initial clustering process, we reminded reviewers to take the findings at face value and not to over interpret them; thus, again remaining close to the paper's original meaning. With respect to understanding the components of complex interventions and developing logic models to explain their mechanisms, we consider this method to be transparent and replicable, particularly in the absence of any gold standard.

### 7.5 Agreements and disagreements with other studies or reviews

We only know of one other published review that has specifically targeted care farming as an intervention for people with mental health problems (Iancu et al., 2013), which included five studies, three of which were RCTs. One of the RCTs was excluded from our review because the intervention was horticulture therapy delivered by a health care professional, rather than therapeutic horticulture delivered by a care farmer (Kam & Siu, 2010). The other UBA study (Cerino, Cirulli, Chiarotti, & Seripa, 2011) was not found by our search, but would not have met our eligibility criteria, being a single activity (therapeutic horse riding). Overall, for the included studies, the reviews are in agreement in so far as quality, scope of outcomes and findings. We agree with Iancu's (2013) view that care farming as a work‐based intervention should be evaluated as a form of vocational rehabilitation, and yet as a robust measure this is lacking from the studies. Iancu (2013) also found three key qualitative themes from three studies relating to disability (distraction, stress release and participation), recovery (viewing the self differently and being socially included), and specific farm experiences (absorption in work and connecting with nature). Our synthesis was more in‐depth and involved more studies but we did find the themes to which Iancu (2013) refers.

Other reviews (one systematic and the other a simple literature review) with a broader nature‐based remit (Annerstedt & Währborg, 2011; Bragg & Atkins, 2016), and also with a narrow but overlapping focus on conservation or horticulture therapy and gardening, exist (Clatworthy, Hinds, & Camic, 2013; Kamioka et al., 2014; Lovell, Husk, Cooper, Stahl‐Timmins, & Garside, 2015). One of the broader reviews, involving 38 papers, included nature‐assisted interventions, wilderness and horticulture therapies, but not care farms, and focused on a wide range of vulnerable groups (Annerstedt & Währborg, 2011), but mostly related to disaffected youth, and those with mental health problems or dementia. The main difference here is the application of a “therapy”, implying the delivery of an intervention by a professional (often health‐based), rather than offering an intervention that is “therapeutic”, as is the case with our review. Some of the studies also included an additional therapeutic component such as psychotherapy or cooking activities, mostly for participants with addiction problems. The contribution of the nature element in these interventions is unclear. As with our review, the authors found that the quality of the studies was mostly low, with often small sample sizes and short term follow‐up (at the end of the intervention). However, most studies reported finding positive outcomes, and the authors conclude that there is a small body of evidence to support the use of nature‐assisted therapies for a range of conditions and social circumstances. The second broad literature review looked at social and therapeutic horticulture, care farming and environmental conservation (Bragg & Atkins, 2016). These interventions were separately covered by the other reviews so are not discussed here. The systematic review on conservation involved volunteers, so did not specifically address impacts of nature‐based interventions on vulnerable populations. The review on horticulture therapy (an intervention that can be included within care farming) included four RCTs involving people with dementia, severe mental illness such as schizophrenia, bipolar disorder, and major depression, as well as frail elderly people in nursing homes and hemiplegic patients after stroke. As with all the reviews reported here, including our own, meta‐analysis was not possible due to heterogeneity in outcomes and across the interventions. Again, the studies were found to be of low quality, but overall there was evidence of effectiveness for improved mental health and behavioural outcomes.

## 8 AUTHORS' CONCLUSIONS

### 8.1 Implications for practice and policy

By far the most studied client group in care farming research is people with mental health problems. In the UK currently, there are more care farms providing support for people with learning difficulties (93% of farms) and ASD (84% of farms) than there are for those with mental health problems (75%) (Bragg et al., 2014). However, only four of the 18 qualitative studies explored the experience of care farming for learning disabilities and autistic spectrum disorder. Similarly, disaffected youth who are supported by around 64% of UK care farms (Bragg et al., 2014) were again the focus of only four studies, with two being quantitative.

Reasons for the intense research interest in mental health problems above other client groups likely reflect a growing concern about increasing mental health problems in modern society (Murray et al., 2012), a lack of choice and availability of treatment options (MIND, 2013) and the impact on the economy through benefit support, absenteeism and unemployment (Centre for Mental Health, 2010). Although the use of nature to support recovery from a range of mental health conditions is not new, the way it is used has evolved over time. Once an adjunct to institutional psychiatric care, it has become part of a community‐based multifunctional “green care” service. However, the evidence for nature as a mental health “treatment option” has not evolved at the same rate as for other more medical approaches. Only recently, through the application of social prescribing, have health care providers and commissioners started to translate the longstanding knowledge that many mental health problems are underpinned by social circumstance (Marmot et al., 2010) and begun to commission services that provide social interventions (CRD, 2015).

Yet even within this approach, green care services are used relatively infrequently when compared to traditional approaches (Bragg & Leck, 2016). Given that, in the UK at least, care farms are underutilized relative to the spaces available on the farms' structured programmes (Bragg et al., 2014), lack of capacity across the broader green care service is not the issue. Lack of access may contribute specifically within more urban areas with fewer green spaces, higher deprivation and lack of transport. Lack of understanding and awareness is however likely to be a major factor. In countries such as Norway, Sweden and the Netherlands, where care farming is well‐established and research is most active, there is greater integration with statutory services (Elsen and Finuola, 2013). In the North and Republic of Ireland there has been an active push to market care farming directly to commissioners combined with the establishment of a network of farms supported by EU funding (Social Farming Across Borders, 2015), and this could be an option in areas where engagement has been low. In addition, in other countries access to care farming has been written into their constitution (https://www.cliclavoro.gov.it/Normative/Legge_18_agosto_2015 n.141).

However, the need to communicate how care farms work and who they are appropriate for is needed in the UK, where healthcare commissioners lack awareness and understanding about care farming and who might benefit (Bragg, Egginton‐Metters, Leck, & Wood, 2015), but this is just one side of the problem. In addition to securing funding through commissioners, there is the dual task of communicating directly to frontline providers, specifically primary care staff and social prescribing facilitators, who have the role of identifying interventions for patients with complex social needs that present as mental health problems. Here, the skill is matching needs to service response, and while some interventions have a clear fit (e.g., debt services, housing support and relationship counselling), others, particularly care farming, may be more challenging to place. There is also a lack of awareness and understanding from patients as to the potential benefits of green care, including care farms, and so as a client‐led approach, green interventions may not be a considered an option. Having developed a theoretical framework and a set of logic models to describe potential mechanisms behind care farming, we now have a basis upon which to inform health and social care commissioners how care farms may work theoretically and for whom they might be suitable.

The studies included in the review had twice as many male as female participants. Reports on the care farming sector in the UK (Bragg et al., 2014) indicate that this is a reflection of the use of care farms by men and women. This preference for care farming by men is of interest to commissioners of mental health services. There is a gender inequality in utilisation of mental health services, where usage is much higher among females (Health and Social Care Information Centre, 2014). It may be that green care interventions are perceived by men to be a less intrusive, and therefore a more acceptable form of support. Findings from the qualitative studies included in this review would seem to support this, where service users refer to the benefits of not forcing early social interactions, where conversations centre on work rather than illness, and where distraction is welcome. This may mean that care farms may be preferred by those wanting less intense personal interventions to improve their mental health.

In regards to “treatment” costs and duration of the intervention, studies included in the current review suggested an intervention duration (averaging around 12 weeks) that is representative of practice and comparable with talking therapies. Although not considered in the current review, the costs of talking therapies are also not dissimilar to care farming (Bragg et al., 2014; MIND, 2013). There is a need to identify a wider range of interventions to address mental ill‐health and allow tailoring to individuals’ personal treatment needs. Providing a greater range of intervention options, such as care farming, would provide choice where there is currently little on offer and has the potential to reduce waiting lists for talking therapies (MIND, 2013). Furthermore, it could help redress gender inequalities in terms of accessing support for mental health problems. Further studies are needed to explore the effectiveness of alternative mental health interventions, such as care farming, with exploration of who they may work for and how.

For the other client groups, the implications for policy and practice from this review are limited. Disaffected youth, particularly those at risk of exclusion from school, potentially represent those most likely to offend, are more likely to have future mental and physical health problems and fewer employment prospects (Parker et al., 2014). Care farms could potentially offer an alternative form of education with qualified educators supporting the delivery of qualifications such as Open College Network Qualifications (Bragg et al., 2014). While this review did not search specifically for CF studies with educational outcomes, in those studies included here none had measured educational outcomes alongside health outcomes. Understanding the impacts on young people's education, behaviour and any inter‐relationships with health would be a valuable future area for study.

The European studies included in the review indicate that systems appear to be in place that allow people with learning disabilities to access green care where it is wanted, or where it is accessible, with funding often provided through local authority personal budgets. It is interesting that people with learning disabilities is the largest client group attending care farms in the UK, but the question of benefit accrued has not been explored in great depth. It is unclear whether individual carers who are in pursuit of support actively seek out organisations such as care farms or whether local authorities are more informed about services available in the community. Regardless, there appears to be a working mechanism that enables those with learning disabilities to have the opportunity to benefit socially and physically from farm work, and this seems to be supported by the qualitative literature.

The most recent patient group to engage with care farms is people with dementia. Although we found little research, we are aware of a number of programmes throughout the UK that are starting to engage people with dementia in nature‐based activities. As part of the King's Fund Enhancing the Healing Environment initiative, a selection of UK hospitals have been working to increase contact with nature (http://www.kingsfund.org.uk/projects/enhancing‐healing‐environment), based on the premise that agitation and number of falls can be reduced. Stepney City Farm's Furry Tails initiative in London has also recently been involved in piloting a scheme to deliver animal handling sessions in an attempt to reduce social isolation in older people and in those with dementia (http://furry‐tales.org.uk/). There are also opportunities within social prescribing schemes to refer older people experiencing social isolation and those with perhaps the earlier stages of dementia to attend care farms, but as with mental health problems, the benefits are yet to be demonstrated.

### 8.2 Implications for research

Contextual descriptions revealed a wide range of activities provided for service users on care farms (see Table 4); however, there was insufficient information to establish whether effects differed according to these. Information was not sufficiently detailed to allow us to determine client specific activities, although logic dictates that some more vulnerable and less independent service user groups are less likely to be involved in heavy traditional farming activities that contribute to productivity. Knowledge about this is important for helping to understand the ways in which care farming might work for different client groups; this is clearly of value to commissioners and other funders of care farms. We know from the qualitative studies that there might be some differences in the intervention components as interpreted by the service users and that there may be differences in the mechanisms of change, but because many studies include mixed client groups and failed to report separate themes, we have limited information.

Care farming research has become an active field in recent years; however, well designed studies are still lacking. There is some evidence, albeit inconsistent, that as a theoretically underpinned intervention, care farming might improve mental health outcomes. The need for a robust evidence base seems most urgent in the mental health field where there is growing concern about the increasing individual and economic burden that mental illness imposes and the limited range of interventions available (Centre for Mental Health, 2010). To progress the evidence, the quality of the research needs to improve.

Our review highlighted how different population groups experience and may benefit from care farms differently. Going forward, research studies should collate data on single population groups so as to provide answers to health and social care commissioners who tend to commission services for specific client groups. We recognise that for care farms, working with only one single population group or not combining groups in activities may be challenging and impractical. However, research can be designed to build the evidence base relevant to different population groups. Evidence on the impact on health is particularly important to the care farming sector as well as health commissioners. Often situated in the third sector, care farms balance income from a range of sources, including grants from charities and private organisations, revenue from selling farm produce, but an important source of income for many care farms in Europe is through funding from public health and social care. Thus demonstrating their contribution to health and social outcomes to secure one of their potentially long term funding sources is important.

One of the aims of this review was to understand how care farming worked for these different client groups. We have observed some differences across the groups with “achievement and satisfaction” and “feeling safe” being potentially more or less important in some groups compared to others. How these convert or contribute to outcomes is unclear, and indeed the general conversion of mechanisms to outcomes is an invisible part of all logic models. What we can glean from these logic models is a sense of which outcomes might be most appropriate for which client group. The mental illness/substance misuse logic model provided the most obvious path from theory to mechanisms and then to outcomes. However, vocational rehabilitation was not adequately addressed and only “work ability” (Lambert, 2014) was measured, but without adequate clarity about its reliability. Returning to work/taking up work could offer important individual financial and well‐being gains, but also, from an economic perspective, can potentially reduce the burden on society from a reduction in health service utilisation and benefits; however, included studies lacked data on these outcomes. This is an area in which commissioners are becoming increasingly interested, so care farming research needs to demonstrate its impact more broadly. More reliable and objective proxy measures for returning to work would be of interest. In addition to broadening its impact in line with anticipated outcomes that fit with explanatory theories, longer‐term follow‐ups beyond 6 months are required. There was some indication that positive outcomes, such as improvements in anxiety and self‐efficacy, may take time to manifest, but this needs to be confirmed.

For disaffected youth, the path from theory to outcomes was not followed, as measured outcomes did not adequately fit with the model. We would suggest that care farming interventions involving disaffected youth use these models to determine the most appropriate outcomes.

The disaffected youth client group was the only one to report findings relating to “reflection”. Children at risk of exclusion from school are at high risk of entering into an adult criminal lifestyle (Audit Commission, 2010), and desistance theory suggests that a period of reflection is a critical early step in the rehabilitation of offenders (Cusson & Pinsonneault, 1986; Farrall & Bowling, 1999), but only if it is supported with interventions that take them beyond this. In this respect, care farming may have the capacity to rehabilitate young people who are at risk of committing offences later in life. In line with this, the other category of mechanism that was present in this client group but not the mental health problems group was “creating a new identity” which again fits with desistance theory. This category was also found in the learning disabilities group and related more to how this client group envisaged themselves as a farmer.

Studies included in the current review used a wide range of measures and concurs with the findings from a previous review of care farming interventions (Iancu et al., 2013). In an area of research where individual studies tend to be underpowered, there is a greater need to be able to combine findings in a meta‐analysis. In the current review, the most commonly applied mental health outcome measures were the Beck Depression Inventory (Beck et al., 1996) and the State‐Trait Anxiety Inventory (Spielberger, 1983), both of which appear to be acceptable to the population group. More fundamentally, this review identified a number of small scale evaluations which used tools that had not undergone psychometric evaluation. We would suggest that researchers select existing reliable and validated tools.

Adopting robust study designs must be matched with capacity to undertake the research, and this is where care farming studies may need to compromise. A lack of service infrastructure across the care farming sector and peripheral relationships with statutory services means that methodically robust large RCTs are very difficult to perform, particularly where income for the intervention is not guaranteed and single client groups at individual farms are quite small in number. In the absence of available studies where data can be combined, larger studies that involve multiple care farms, possibly operating in a network, are an option. These would ideally require agreed standardised criteria for referrals across multiple healthcare organisations.

In general, we recommend that a more cohesive approach to care farming research be adopted. This means understanding the needs of commissioners and thinking beyond individual CF research studies. Green care has potentially much to offer, but currently cannot prove its worth until more robust methodologies and strategically aligned research are conducted.

## ROLES AND RESPONSIBILITIES

- Content: J. M., N. W., H. E., R. B., M. E. and M. G. L.
- Systematic review methods: J. M., N. W., and H.E.
- Information retrieval: J. W. and T. V.
- Manuscript preparation: J. M., N. W., H. E., R. B., M. E., M. G. L., C. B., Z. R., J. C., D. S. and S. T.

## Supporting information

Supplementary information

## Footnotes

1 Learning disabilities is an umbrella term for a range of conditions including Down's syndrome, fragile X syndrome and cerebral palsy. ASD can also be included here, but not all those with ASD have a learning disability.

2 Positive changes in self‐image reported in one paper (Granerud & Eriksson, 2014) suggested to mean changes in personal identity.

3 Studies reported combined data for mental ill‐health and substance misuse so there is one logic model for both groups.
