# Supplementary material for: The impact of care farms on quality of life, depression and anxiety among different population groups: A systematic review
Source: Campbell Syst Rev. 2019 Nov 26;15(4):e1061. doi: 10.1002/cl2.1061 (PMC8534033; doi:10.1002/cl2.1061)
Supplement: Supplementary file 1 — Supplementary information [file CL2-15-e1061-s001.docx]

# Appendices

## Components of search forming electronic search strategy

| Care Farms | Farm settings | Specific Agricultural /Gardening therapies | Agriculture, Gardening or Countryside activities | Rehabilitation or Therapy | Items to remove |
| --- | --- | --- | --- | --- | --- |
| care farms  care farming  social farms  social farming  care ranches  rehabilitation farms  rehabilitation farming  rehab ranches  residential farm  educational farm  community farm  therapeutic farm  re-entry gardening program  horticultural vocation program  horticultural treatment | farms  ranches  land  agricultural setting  agricultural environment  agricultural facility  aquaculture  market garden  horticultural unit  horticultural garden  small holding  nursery garden  hydroponics  green space | nature therapy  green care  green exercise  healing garden  sensory garden  horticultural therapy  animal-assisted therapy  equine-assisted therapy  hippotherapy  onotherapy  ecotherapy | agriculture (Mesh only)  gardening  horticulture  cart driving  crafts  woodwork  woodland restoration  woodland management  conservation  paddock maintenance  farm mechanics  bird watching  nature watching  beekeeping  Apiary | rehabilitation (Mesh only)  rehabilitation centres  correctional  rehabilitation floating sub heading  therapy  therapies  therapy floating sub heading  delivery of health care | veterinary medicine  animal experiment  animal –only studies (no human involvement) |

## electronic search strategy

Ovid MEDLINE(R) <1946 to November Week 2 2014>

1 (care adj3 (farm or farms or farming or ranch or ranches)).tw. (61)

2 (rehab* adj3 (farm or farms or farming or ranch or ranches)).tw. (6)

3 ("social farm*" or "therapeutic farm*").tw. (2)

4 or/1-3 [care/rehab farms or ranches] (69)

5 (farm* or farms* or ranch or ranches or land).tw. (69177)

6 (agricultur* adj2 (setting* or environment* or facility or facilities)).tw. (990)

7 (aquacultur* or hydroponic* or "market garden*" or "horticult* unit*" or "horticult* garden*" or (small adj3 holding*) or (nurser* adj1 garden*)).tw. (6152)

8 Agriculture/ and *"Delivery of Health Care"/ (45)

9 "green space*".tw. (266)

10 or/5-9 [farms] (75335)

11 exp nature/ (615)

12 (nature* adj1 (therap* or intervention* or rehabilitation)).tw. (94)

13 (green adj2 (care or exercise)).tw. (48)

14 ((Heal or healing or sensory) adj3 garden*).tw. (19)

15 exp Horticultural Therapy/ (14)

16 exp animal assisted therapy/ or exp equine-assisted therapy/ (218)

17 "animal* assisted".tw. (172)

18 ((riding or equine) adj1 (therap* or intervention* or rehabilitation)).tw. (71)

19 (horticultur* adj1 (treatment* or "vocation* program*" or therap* or intervention* or rehabilitation)).tw. (31)

20 (forest adj (therap* or intervention*)).tw. (10)

21 onotherap*.tw. (1)

22 hippotherap*.tw. (72)

23 ecotherap*.tw. (3)

24 social horticultur*.tw. (0)

25 or/11-22 [specific agric/gardening therapies] (1218)

26 exp agriculture/ (61996)

27 exp *Gardening/ (336)

28 (garden* or horticultur*).tw. (7720)

29 ((cart or carts) adj2 (drive or driving)).tw. (0)

30 craft*.tw. (3143)

31 woodwork*.tw. (319)

32 (woodland* adj3 (restor* or manage*)).tw. (29)

33 exp "Conservation of Natural Resources"/ (32365)

34 conservation*.tw. (51521)

35 (paddock* adj2 maintenance).tw. (0)

36 farm mechanics.tw. (0)

37 ((bird* or nature) adj3 watch*).tw. (44)

38 (apiar* or beekeep* or "bee keep*" or "bee yard*" or beehive*).tw. (674)

39 or/26-38 [Agriculture, Gardening, Countryside activities] (144409)

40 rehabilitation/ or "activities of daily living"/ or animal assisted therapy/ or equine-assisted therapy/ or art therapy/ or bibliotherapy/ or dance therapy/ or early ambulation/ or exercise therapy/ or motion therapy, continuous passive/ or muscle stretching exercises/ or plyometric exercise/ or resistance training/ or occupational therapy/ or recreation therapy/ or rehabilitation, vocational/ (119772)

41 rehabilitation centers/ (7047)

42 rh.fs. (173336)

43 correctional.tw. (2183)

44 (therapy or therapies).tw. (1313901)

45 th.fs. (1496682)

46 or/40-45 [Rehabilitation or Therapy] (2692671)

47 39 and 46 [Agric/Garden/Countryside Rehab or Therapy] (5028)

48 25 or 47 [All Agric related therapies or rehab] (6198)

49 10 and 48 [Farms and Agric related therapies or rehab] (386)

50 4 or 49 [Care Farms or Agric therapy/rehab at farm setting] (444)

51 exp Horticultural Therapy/ or "re-entry garden*".tw. [feedback from campbell, with less emphasis on farm or Agri setting] (14)

52 4 or 49 or 51 [Care Farms or Agric therapy/rehab at farm setting] (458)

53 exp Veterinary Medicine/ or exp Animal Experimentation/ (28317)

54 exp animals/ not (exp animals/ and exp humans/) (4094644)

55 53 or 54 [Items to remove] (4110647)

56 52 not 55 (290)

## Screening form (inclusion /exclusion

| **Study ID:** | **Report ID :** | Date form completed: |
| --- | --- | --- |
| First author: | Year of study: | Reviewer: |
| Citation: | | |

**1. General Information**

| Publication type Journal Article ⬜ Abstract ⬜ Other (specify e.g. book chapter)___________________ | |
| --- | --- |
| Country of study: | |
| Funding source of study: | Potential conflict of interest from funding? Y / N / unclear |

**2. Study Eligibility**

| **Study Characteristics** | | | | **Page/ Para/ Figure #** |
| --- | --- | --- | --- | --- |
| **Type of study** | A.  ⬜ RCT with individual randomisation  ⬜ RCT with cluster randomisation | B.  ⬜ Quasi randomised or  ⬜ Quasi cluster randomised   - (randomisation by eg case number / DoB) | |  |
|  | C.  ⬜ Interrupted Time Series (ITS)  ⬜ At least 3 time points before and 3 after the intervention  ⬜ Clearly defined intervention point | D.  ⬜ Prospective cohort study  ⬜ At least 6 months duration  ⬜ Drop-out figures / characteristics detailed | |  |
|  | E.  ⬜ Case control study⬜ Baseline characteristics comparable  ⬜ Potential confounders reported | F.  ⬜ Controlled Before and After (CBA) study  ⬜ Contemporaneous data collection  ⬜ Comparable control site | |  |
|  | G.  ⬜ Other design with estimated effect sizes (specify):  …………………………………………………….. | H.  ⬜ Uncontrolled Before and After study including process evaluations | |  |
|  | I.  ⬜ Qualitative (specify):  (Please complete Section 2 and thereafter use the Qualitative data extraction form) | J.  ⬜ Single subject study  ⬜ review, commentary, editorial | |  |
|  | k. ⬜ Descriptive quantitative studies (may include comparator setting but without statistical analyses comparing outcomes. *Complete Section 1 only* |  | |  |
|  | *Boxes A to I meet the inclusion criteria*  *Box J is an exclusion criterion*  Decide to include: Yes ⬜ No ⬜ 🡪**Exclude** Unclear ⬜ | | |  |
|  | Description in text: | | |  |
| **Participants** | Describe the participants included:  ⬜ Offenders serving community order  ⬜ Drug / alcohol problems (specify):  …………………………………………………………..  ⬜ Learning difficulties  ⬜Other (specify):  ……………………………………………………………… | | ⬜ Mental health problems (specify):  ……………………………………………………….  ⬜ Long term conditions (specify):  …………………………………………………………..  ⬜Young people challenging behaviour  ⬜ Palliative care |  |
|  | *Do the participants meet the criteria for inclusion?* | | Yes ⬜ No ⬜ 🡪**Exclude** Unclear ⬜ |  |
|  | *Is the intervention inside a prison or hospital setting?* | | No ⬜Yes ⬜ 🡪**Exclude** Unclear ⬜ |  |

| **The intervention** | Definition of care farm:  *use of commercial farms and agricultural landscapes as a base for promoting mental and physical health through normal farming activities. Specifically providing structured supervised programme of health, vocational or social activities a farm related activities for vulnerable people.* | |  |
| --- | --- | --- | --- |
|  | Do the activities take place on a farm that meets the above criterion? | Yes ⬜ No ⬜ 🡪**Exclude** Unclear ⬜ |  |
|  | Are the activities classified as ‘therapy’ rather than activities that are therapeutic? | Yes ⬜🡪**Exclude** No ⬜ Unclear ⬜ |  |
|  | *If ‘therapy’, specify* | ………………………………………………………………………. |  |
|  | *Is the intervention a petting farm?* | Yes ⬜🡪**Exclude** No ⬜ Unclear ⬜ |  |
|  | *Is the intervention a ‘one off’ educational visit?* | Yes ⬜🡪**Exclude** No ⬜ Unclear ⬜ |  |
|  | *Is the intervention a single activity such as gardening, horse riding* | Yes ⬜🡪**Exclude** No ⬜ Unclear ⬜ |  |
|  | *Does the intervention also include substantial component of other non-horticultural intervention offered outside of the farm ĕ.g music at a recreation centre* | Yes ⬜🡪**Exclude** No ⬜ Unclear ⬜ |  |

**Summary of Assessment for Inclusion**

| **Include in review ⬜ Exclude from review ⬜ Lack of consensus / requires discussion ⬜** | |
| --- | --- |
| Consensus resolved Yes ⬜ No ⬜ | Further details required? Yes ⬜ No ⬜ |
| Contact details of authors: | Reviewer responsible for contacting authors:  Date contacted: ………………………………………….. |
| **Notes:** | |

**DO NOT PROCEED WITH THIS FORM IF THE PAPER EXCLUDED FROM REVIEW**

## Description of care farm programmes (and comparison settings)

**CARE FARM PROGRAMME**

| 1. **Farm Programme** | |  |
| --- | --- | --- |
| Name & location: | |  |
| Aims: | |  |
| Entry/referral criteria to farm: | |  |
| Activities available: | |  |
| Activities specific to study participants (for each indicate if this was indoor /outdoor / not specified : | |  |
| Group work: yes ⬜ no ⬜ not specified ⬜ | |  |
| Hrs per day on farm / time already spent at start of study / Planned duration on farm (e.g 25 sessions): |  |  |
| Details of political / organisational context / any partnerships / contracts referred to |  |  |
| Source of funding to attend care farm (self / organisation contract / personal budget) |  |  |
| Age  Details: specify if the age is given as a median, mean, SD and range if possible) | Intervention:  Control:  Details: |  |
| Sex | Intervention:  Control: |  |

| 1. **Comparison** |  |
| --- | --- |
| Name and details of comparison program/intervention: |  |
| Aims: |  |
| Setting: |  |
| Entry/referral criteria: |  |
| Duration of comparison intervention: |  |
| Activities available: |  |

**Do not complete subsequent sections if the study is purely descriptive**

## Qualitative Data Extraction

| ID: | Yr: | Reviewer: |
| --- | --- | --- |
| Authors: |  | Country: |

| 1. **STUDY METHODS** | | **Pg / Para / Fig** |
| --- | --- | --- |
| Stated aim of study: |  |  |
| Independent study:  Yes / No | A process evaluation (part of larger study): Yes / No  Reference: ……………………………………….. |  |
| Data collection (circle): | Interviews (individual) / focus groups / diary records / Observation/ other (state: …………………………………) |  |
| Theoretical concepts & reference (e.g Attention restoration theory) | *Please include reference for the key paper describing the Theory or Conceptual framework used – i.e. from the Reference list in the paper being reviewed*.  *If none stated, please record* |  |
| Qualitative framework (e.g grounded theory) | *If none or not applicable, please record*  *please specify whether this qualitative framework was used throughout the study, or only for one component e.g. the analysis* |  |

| 1. **STUDY PARTICIPANTS** | *(if several groups, please indicate which group each section of data corresponds to)* | **Pg / Para / Fig** |
| --- | --- | --- |
| Selection criteria: |  |  |
| Method of recruitment (approached / invited |  |  |
| Sampling (e.g. convenience, purposive, theoretical) |  |  |
| No. interviews / observations per participant & duration between data collection points |  |  |
| Total participants (n): |  |  |
| Gender (n/%): |  |  |
| Education(n/%) |  |  |
| Socio-economic status(n/%) |  |  |
| Ethnic make-up (n/%) |  |  |
| Age: *Please indicate whether mean(SD)/median/range (all that apply)* |  |  |

| 1. **RESULTS** | | | | | | |
| --- | --- | --- | --- | --- | --- | --- |
| Indicate ‘Y’ in Quotation box if supported by ***verbatim text.***  ^*^1^st^ order construct (from the participants – more inductive) / 2^nd^ order construct (author defined from theory or preconceptions – more deductive) | | | | | | |
|  | Theme | Pg / Para /Fig | 1^st^ /2^nd^ order construct^*^ | Quotation (Yes/ No) | Outcome or mechanism | Outcome Domain: Health /Well-being /Social/ Criminal Justice |
| 1 |  |  |  |  |  |  |
| 2 |  |  |  |  |  |  |
| 3 |  |  |  |  |  |  |

## Quantitative Data Extraction

**STUDY DETAILS**

| **Study intention** | **Descriptions as stated in the report/paper** | **Page/ Para/ Figure #** |
| --- | --- | --- |
| Aim of study | *What was the study designed to assess? Are these clearly stated?* |  |

**METHODS**

|  | **Descriptions as stated in the report/paper** | **Page/ Para/ Figure #** |
| --- | --- | --- |
| Method/s of recruitment of participants  *(How were potential participants approached and invited to participate? Where were participants recruited from? Does this differ from the intervention setting?)* |  |  |
| Inclusion/exclusion criteria for participation in study |  |  |
| Representativeness of sample: Are participants in the study likely to be representative of the target population? |  |  |
| Total number of intervention groups |  |  |
| Sample size calculation:  What assumptions were made?  Were these assumptions appropriate? | *Yes/No/Unclear*  *If Yes provide details:* |  |
| If RCT/quasi-RCT, what was the unit of randomisation? (record if not applicable)  Allocation by individuals or cluster/groups |  |  |
| What was the unit of analysis?  **Is this the same as the unit of randomisation?** | *(Yes/No/Unclear)* |  |
| Statistical methods used and appropriateness of these methods | *(Check with your statistician if unsure about appropriateness)* |  |

**RESULTS**

| **Participants**  *Include if relevant* | **Include information for each group (i.e. intervention and controls) under study** (record ‘not reported’ if relevant but missing; record ‘not applicable’ if not relevant) | **Page/ Para/ Figure #** |
| --- | --- | --- |
| 1. What percentage of selected individuals agreed to participate? |  |  |
| 1. If RCT, total number randomised (or total pop. at start of study for NRCTs) |  |  |
| 1. Number allocated to each intervention group (no. of individuals) |  |  |
| 1. For cluster trials, number of clusters, number of people per cluster |  |  |
| 1. Where there any significant baseline imbalances? | Yes ⬜ No ⬜ Unclear ⬜  Details: |  |
| 1. Number and reason for (and socio-demographic differences of) withdrawals and exclusions for each intervention group |  |  |
| 1. Were participants who entered the study adequately accounted for? |  |  |
| 1. What percentage of participants completed the study? |  |  |
| 1. What percentage of participants received the allocated intervention or exposure of interest? |  |  |
| 1. Is the analysis performed by intervention allocation status (intention to treat) rather than the actual intervention received? Have any attempts been made to impute missing data? |  |  |
| 1. Age   Details: specify if the age is given as a median, mean, SD and range if possible) | Intervention:  Control:  Details: |  |
| 1. Sex | Intervention:  Control: |  |
| 1. Race/Ethnicity |  |  |
| 1. Education   Details: give specifics of the units/categories | Details: |  |
| 1. Socio-economic status 2. Details: give specifics of the units/categories | Details: |  |
| 1. Place of residence 2. Details: give specifics of the units/categories | Details: |  |
| 1. Social Capital – See Note A at end of section 2. Details: give specifics of the units/categories | Details: |  |
| 1. **Subgroups** | *Enter a description of any participant subgroups from this paper to be analysed in the review.* |  |

**Intervention Group** (copy and paste table for each Intervention group)

| **Group name:** | *(State brief name for this intervention group.)* | **Page/ Para/ Figure #** |
| --- | --- | --- |
| Details of intervention or control condition *(Include if relevant in sufficient detail for replication)* | | |
| - Theoretical basis (include key references) |  |  |
| - Did the intervention include strategies to address diversity/disadvantage? | *Enter a description of any relevant strategies* |  |
| - Delivery (eg. Stages (sequential or simultaneous), timing, frequency, duration, intensity, fidelity – proximal indicators) |  |  |
| - Providers (who, number, education/training in intervention delivery, ethnicity etc. if potentially relevant to acceptance and uptake by participants |  |  |
| - Co-interventions |  |  |
| Duration of follow-up |  |  |
| Was sustainability discussed by the authors? Was is a consideration in study development? |  |  |
| Economic variables ie costs of the intervention, and changes in other (eg health care) costs as result of intervention^^[[1]](#footnote-1)^♠^ | Yes ⬜ 🡪**List in Outcome section if appropriate**  No ⬜ Unclear ⬜  Details: |  |
| Other economic information (from a societal, non-healthcare view – e.g. lost wages, time) | Yes ⬜  No ⬜  Details: |  |
| Resource requirements to replicate intervention (e.g. staff numbers, hours of implementation, equipment?) |  |  |
| **Subgroups** | ***Enter a description of any intervention subgroups from this report to be analysed in the review.*** |  |
| What are the moderators/mediators of changes stated in the study? |  |  |
| Was a process evaluation conducted? | *What components were included in the process evaluation? (eg. dose, frequency, consistency, implemented as intended etc)* |  |

**Outcomes**

*(This table is set up for 2 outcome measures to save space. Copy and paste table as often as required*)

| **Question** | **Outcome 1** | **Page/ Para/ Figure #** | **Outcome 2** | **Page/ Para/ Figure #** |
| --- | --- | --- | --- | --- |
| Is there an analytic framework applied (e.g. logic model, conceptual framework)? |  |  |  |  |
| Outcome definition (with diagnostic criteria if relevant) |  |  |  |  |
| Type of outcome: Heath, Well-being, Social, Criminal Justice, Other (specify) |  |  |  |  |
| What measure/instrument was used to assess outcome |  |  |  |  |
| Is this a primary outcome? Yes, no, unclear |  |  |  |  |
| Time points measured |  |  |  |  |
| Time points reported |  |  |  |  |
| Is there adequate latency for the outcome to be observed? |  |  |  |  |
| Is the measure repeated on the same individuals or redrawn from the population / community for each time point? |  |  |  |  |
| Unit of measurement (if relevant) |  |  |  |  |
| For scales – upper and lower limits and indicate whether high or low score is good |  |  |  |  |
| How is the measure applied? Telephone survey, mail survey, in person by trained assessor, routinely collected data, other |  |  |  |  |
| How is the outcome reported? Self-reported or observation or clinical test |  |  |  |  |
| Is this outcome/tool validated? |  |  |  |  |
| …And has it been used as validated? |  |  |  |  |
| Is it a reliable outcome measure? |  |  |  |  |
| Is there adequate power for this outcome? |  |  |  |  |
| Outcomes measured at population level or individual level? |  |  |  |  |
| Were outcomes analysed by subgroup? Indicate the letters of those that outcomes were analysed by (place of residence, race, occupation, gender, religion, education, SES, social capital (see note A) |  |  |  |  |

Note A: Examples of social capital that are reported in surveys (taken from ONS (http://www.ons.gov.uk/ons/guide-method/user-guidance/social-capital-guide/the-social-capital-project/guide-to-social-capital.html):

- **levels of trust** - for example, whether individuals trust their neighbours and whether they consider their neighbourhood a place where people help each other
- **membership** - for example, to how many clubs, societies or social groups individuals belong
- **networks and how much social contact individuals have in their lives** - for example, how often individuals see family and friends

**Results for comparison group(s) (Cont’d)**

Copy and paste the appropriate table for each outcome and subgroup at each timepoint, including baseline

**For RCT/CRCT/Quasi-RCT**

| **Dichotomous/ Binary outcome** | | | | | page/para/fig |
| --- | --- | --- | --- | --- | --- |
| Describe Comparison (i.e usual care etc) |  | | | |  |
| Outcome |  | | | |  |
| Subgroup |  | | | |  |
| Timepoint |  | | | |  |
| **Results** | **Intervention** | | **Comparison** | |  |
|  | Events | No. participants | Events | No. participants |  |
|  |  |  |  |  |  |
| No. of missing participants and reasons |  | |  | |  |
|  |  | | | |  |
| Reanalysis required? (specify -  (e.g. correlation adjustment) |  | | | |  |
| Reanalysis possible? | *yes/no/unclear* | | | |  |
| Reanalysed results |  | | | |  |

**For RCT/CRCT**

| **Continuous outcome** | | | | | | | page/para/fig |
| --- | --- | --- | --- | --- | --- | --- | --- |
| Describe Comparison (i.e usual care etc) |  | | | | | |  |
| Outcome |  | | | | | |  |
| Subgroup |  | | | | | |  |
| Timepoint |  | | | | | |  |
| Post-intervention or change from baseline? |  | | | | | |  |
| **Results** | **Intervention** | | | **Comparison** | | |  |
|  | Mean | SD (or other variance) | No. participants | Mean | SD (or other variance) | No. participants |  |
|  |  |  |  |  |  |  |  |
| No. missing participants and reasons |  | | |  | | |  |
| Any other results reported |  | | | | | |  |
| Reanalysis required? (specify) |  | | | | | |  |
| Reanalysis possible? | *yes/no/unclear* | | | | | |  |
| Reanalysed results |  | | | | | |  |

**For RCT/CRCT**

| **Generic inverse variance method (Odds ratio/RR/SMD/MD)** | | | | | Page/para/figure |
| --- | --- | --- | --- | --- | --- |
| Describe Comparison (i.e usual care etc) |  | | | |  |
| Outcome |  | | | |  |
| Subgroup |  | | | |  |
| Timepoint |  | | | |  |
| Results | Effect estimate | SE (or other variance) | Intervention no. | Control no. |  |
|  |  |  |  |  |  |
| No. missing participants and reasons |  | | | |  |
| Any other results reported |  | | | |  |
| Reanalysis required? (specify) |  | | | |  |
| Reanalysis possible? | *yes/no/unclear* | | | |  |
| Reanalysed results |  | | | |  |

**For CBA, or studies with an intervention and comparison group**

|  |  | | Page/para/fig |
| --- | --- | --- | --- |
| Describe Comparison (i.e usual care etc) |  | |  |
| Assignment | How were control and treatment groups selected?? Is there likely to be an effect if these were the opposite way? | |  |
|  | Contemporaneous data collection? | |  |
| Outcome |  | |  |
| Subgroup |  | |  |
| Timepoint |  | |  |
| Post-intervention or change from baseline? |  | |  |
|  | **Intervention** | **Comparison** |  |
| No. participants  measured |  |  |  |
| No. missing participants and reasons |  |  |  |
| Baseline result (with variance measure) |  |  |  |
| Post-intervention results (with variance measure) |  |  |  |
| Change (Post – baseline) (with variance measure) |  |  |  |
| Difference in change (intervention – control) (with variance measure) |  | |  |
| Any other results reported |  | |  |
| Reanalysis required? (specify) |  | |  |
| Reanalysis possible? | *yes/no/unclear* | |  |
| Reanalysed results |  | |  |

**For Pre-post studies, ITS, or studies without a comparison group**

|  | | | | | | **Page/para/fig** |
| --- | --- | --- | --- | --- | --- | --- |
| Describe Comparison (i.e usual care etc)parison |  | | | | |  |
| Outcome |  | | | | |  |
| Subgroup |  | | | | |  |
| Length of timepoints measured |  | | | | |  |
| Snapshot or interval measured |  | | | | |  |
| No. participants measured |  | | | | |  |
| No. missing participants and reasons |  | | | | |  |
|  | Pre-intervention | | | Post-intervention | |  |
| No. of timepoints measured |  | | |  | |  |
| Mean value (with variance measure) |  | | |  | |  |
| Difference in means (post – pre) |  | | | | |  |
| Percent relative change |  | | | | |  |
| Result reported by authors (with variance measure) |  | | | | |  |
| Reanalysis required? (specify) |  | | | | |  |
| Reanalysis possible? | *yes/no/unclear* | | | | |  |
| Individual time point results |  | | | | |  |
| Read from figure? | *yes/no* | | | | |  |
| Reanalysed results | Change in level | SE | Change in slope | | SE |  |
|  |  |  |  | |  |  |

**Other relevant information**

| Were outcomes relating to harms/unintended effects of the intervention described? Include any data for these in the outcomes tables above |  |  |  |
| --- | --- | --- | --- |
| Potential for author conflict *ie. evidence that author or data collectors would benefit if results favoured the intervention under study or the control* |  |  |  |
| Key conclusions of the study authors |  |  |  |
| Could the inclusion of this study potentially bias the generalisability of the review? Equity pointer: Remember to consider whether disadvantaged populations may have been excluded from the study. |  | | |
| Is there potential for differences in relative effects between advantaged and disadvantaged populations? (e.g. are children from lower income families less likely to wear bicycle helmets) |  | | |
| Are interventions likely to be aimed at the disadvantaged? (e.g. school meals aimed at poor children). |  | | |
| Issues affecting directness  (*Note any aspects of population, intervention, etc. that affect this study’s direct applicability to the review question)* |  | | |
| References to other relevant studies |  | | |
| Additional notes by review authors |  | | |
| Correspondence required for further study information (from whom, what and when) |  | | |

## Quality Assessment for Qualitative Studies

**COREQ tool adapted V0.2 (8/5/12) (Long & Godfrey, 2004; Tong, Sainsbury, & Craig, 2007b). Item 10 added by team following piloting of data extraction forms**

| **ID:** | **Yr:** | **Reviewer:** |
| --- | --- | --- |
| **Authors:** |  | **Country:** |

- *Please record queries for each item at the end of the tool.*
- *Please record N/A for not applicable – These items will be deducted from the overall potential score*

| No. | | Item | Yes (2) | In Part (1) | No (0) |
| --- | --- | --- | --- | --- | --- |
| **Background** | | |  |  |  |
|  | (L) Is it clear what is being studied? | |  |  |  |
| **Research team and reflexivity** | | |  |  |  |
| *Personal characteristics* | | |  |  |  |
|  | Is it clear which author(s) conducted the interviews or focus groups? | |  |  |  |
|  | Do they state their occupation at the time of the study? | |  |  |  |
|  | Is the gender of the researcher clear? | |  |  |  |
|  | Is the researcher’s experience / training reported? | |  |  |  |
|  | Were the characteristics of the interviewer reported? *(bias, assumptions, reasons and interests the topic)* | |  |  |  |
| *Relationships established* | | |  |  |  |
|  | Is there evidence that the researcher/interviewer established a relationship with the participant before the study commenced? (i.e. had informal chats with the patient by telephone or face to face before the actual interview) | |  |  |  |
|  | (New)Did the researcher/interviewer indicate if there was a pre-existing relationship (e.g had nursed or treated) with the participant and if so, was this described? | |  |  |  |

| **No.** | | **Item** | **Yes (2)** | **In Part (1)** | **No (0)** |
| --- | --- | --- | --- | --- | --- |
| **Study Design** | | |  |  |  |
| *Analytic framework* | | |  |  |  |
|  | Was use of an analytic framework mentioned? *(e.g. grounded theory, discourse analysis, ethnography, phenomenology, content or thematic analysis.)* | |  |  |  |
|  | Is the process of analysis consistent with the analytical framework stated? | |  |  |  |
| *Participant selection* | | |  |  |  |
|  | Was the method for participant selection reported? (purposive, convenience, consecutive, snowball) | |  |  |  |
|  | Does the study state how participants were approached? *(face-to-face, telephone, email)* | |  |  |  |
|  | Does the study state how many took part in the interviews/focus group/observations? | |  |  |  |
|  | Does the study state how many refused or dropped out and does it provide reasons? | |  |  |  |
| *Setting* | | |  |  |  |
|  | Is the data collection site stated and is the site conducive to open discussion? | |  |  |  |
|  | Does the researcher state if anyone else was present during the interviews? | |  |  |  |
|  | Are the relevant characteristics of the sample reported *(demographics)*? | |  |  |  |
| *Data collection* | | |  |  |  |
|  | Were questions, prompts, guides provided by the authors? | |  |  |  |
|  | Does the author say how many interviews, focus groups/observations were carried out? | |  |  |  |
|  | Did the author report how data was recorded? | |  |  |  |
|  | Was audio or visual methods used to record/ collect the data? | |  |  |  |
|  | Does the study state if supplementary field notes were made during/after the interview or focus groups/observations? | |  |  |  |
|  | Was the duration of the interviews or focus groups or observation reported? | |  |  |  |
|  | Was data saturation discussed? | |  |  |  |
| **Data analysis and findings** | | |  |  |  |
| *Data analysis* | | |  |  |  |
|  | (L) Is the description of data analysis adequate? *(does it allow reproduction, steps taken to avoid selectivity, provision of a coding tree)* | |  |  |  |
|  | (L) Is there evidence of effort to establish validity to support the analysis? (searching for negative cases, use of multiple sources). | |  |  |  |
|  | Does the study report the number of coders involved? | |  |  |  |
|  | Does the author state if themes were identified in advance or from the data? | |  |  |  |
|  | Does the author report if management software was used? | |  |  |  |
|  | Did the authors report checking back with informants over interpretation? | |  |  |  |
| *Reporting* | | |  |  |  |
|  | Does the author illustrate their themes with participant quotations? | |  |  |  |
|  | Are all participant quotations labelled according to participant? | |  |  |  |
|  | Do the quotations reflect the findings? | |  |  |  |
|  | Do the quotations represent a range of participants? *( i.e not all from one participant if sample size greater than one!)* | |  |  |  |
|  | Were major themes clearly presented in the findings? | |  |  |  |
| **Ethics** | | |  |  |  |
|  | (L) Does the study report if ethical approval was obtained? | |  |  |  |
|  | (L)Was informed consent obtained from all study participants? | |  |  |  |

| Did you answer "yes" to more than half the items?: Yes ⬜ No ⬜ |  |
| --- | --- |
| Is there agreement between reviewers? Yes ⬜ No ⬜  Items with disagreement: ……………………………………………  Consensus reached?: Yes ⬜ No ⬜ | Contact the authors? Yes ⬜ No ⬜  Author contact details:  Contacting Reviewer: ………………………………………………………  Date contacted: ……………………………………………………………… |
| **Notes:** | |

## Risk of bias assessment for RCTs, CRCTs, CBAs & ITS*

Please refer to Chapter 8 - *Table 8.5.c: Criteria for judging risk of bias in the ‘Risk of bias’ assessment tool and to the Cochrane EPOC Group’s guidance for assessing* **Risk of bias for studies with a separate control group (RCTs, CRCTs, CBAs) and Risk of bias for interrupted time series studies** (Appendix 3) for additional guidance for scoring Yes/No/Unclear. Note that the table below includes items from both EPOC tools.

*The ITS tool has been incorporated into the bottom of the table and all items for ITS studies are denoted by ITS preceding the risk of bias question.

| **Domain** | **Review authors’ judgement*** | **Description** | **Page/ Para/ Figure #** |
| --- | --- | --- | --- |
| **Was the allocation sequence adequately generated?** | **Yes / No / Unclear** | ***Describe the method used to generate the allocation sequence in sufficient detail to allow an assessment of whether it should produce comparable groups.*** |  |
| **Was allocation adequately concealed?** | **Yes / No / Unclear** | ***Describe the method used to conceal the allocation sequence in sufficient detail to determine whether intervention allocations could have been foreseen in advance of, or during, enrolment.*** |  |
| **Were baseline outcome measurements similar?** | **Yes/No/Unclear** | ***Note whether baseline outcome measurements were reported and whether there were any important differences between groups. If there were important differences between groups, note whether appropriate adjusted analysis was performed to account for this.*** |  |
| **Were baseline characteristics similar?** | **Yes/No/Unclear** | ***Note whether baseline characteristics were reported and whether there were any important differences between groups.*** |  |
| **Were incomplete outcome data adequately addressed?**  ***Assessments should be made for each main outcome (or class of outcomes).*** | **Yes / No / Unclear** | ***Describe the completeness of outcome data for each main outcome, including attrition and exclusions from the analysis. State whether attrition and exclusions were reported, the numbers in each intervention group (compared with total randomized participants), reasons for attrition/exclusions where reported, and any re-inclusions in analyses performed by the review authors.*** |  |
| **Was knowledge of the allocated intervention adequately prevented during the study?**  ***Separate assessments should be made for relevant groups of people involved in the study i.e participants, outcome assessors, investigators, data assessors etc*** | **Yes / No / Unclear** | ***Describe all measures used, if any, to blind study participants and personnel from knowledge of which intervention a participant received. Provide any information relating to whether the intended blinding was effective, or whether blinding was appropriate.***   1. Participants – yes, no, unclear *[record supporting statement from study].* 2. Investigators – yes, no, unclear *[record supporting statement from study].* 3. Outcomes assessors – yes, no, unclear *[record supporting statement from study].*   Data assessors – yes, no, unclear *[record supporting statement from study].* |  |
| **Was the study adequately protected against contamination?** | **Yes/No/Unclear** | ***State whether and how the possibility of contamination was minimised by the study design/implementation.*** |  |
| **Are reports of the study free of suggestion of selective outcome reporting?**  ***Assessments should be made for each main outcome (or class of outcomes).*** | **Yes / No / Unclear** | ***State how the possibility of selective outcome reporting was examined by the review authors, and what was found.*** |  |
| **Other sources of bias** | **Yes / No / Unclear** | ***State any important concerns about bias not addressed in the other domains in the tool.*** |  |
| **ITS: Was the intervention independent of other changes?** | **Yes/No/Unclear** | ***Describe whether or not the intervention occurred independently of other changes over time and whether or not the outcomes may have been influenced by other confounding variables/historic events during the study period.*** |  |
| **ITS: Was the shape of the intervention effect pre-specified?** | **Yes/No/Unclear** | ***State whether or not the point of analysis was the point of intervention. If not, describe whether a rationale for the shape of the intervention effect was given by the study authors.*** |  |
| **ITS: Was the intervention unlikely to affect data collection?** | **Yes/No/Unclear** | ***Describe whether or not the intervention was likely to affect data collection and what the potential impact might have been.*** |  |
| **ITS: Was knowledge of the allocated interventions adequately prevented during the study?**  ***Separate assessments should be made for relevant groups of people involved in the study i.e participants, outcome assessors, investigators, data assessors etc*** | **Yes/No/Unclear** | ***Describe all measures used, if any, to blind study participants and personnel from knowledge of which intervention a participant received. Provide any information relating to whether the intended blinding was effective, or whether blinding was appropriate.***   1. Participants – yes, no, unclear *[record supporting statement from study].* 2. Investigators – yes, no, unclear *[record supporting statement from study].* 3. Outcomes assessors – yes, no, unclear *[record supporting statement from study].*   Data assessors – yes, no, unclear *[record supporting statement from study].* |  |
| **ITS: Was incomplete outcome data adequately addressed?**  ***Assessments should be made for each main outcome (or class of outcomes).*** | **Yes/No/Unclear** | ***Describe the completeness of outcome data for each main outcome, including attrition and exclusions from the analysis. State whether attrition and exclusions were reported, the numbers in each intervention group (compared with total randomized participants), reasons for attrition/exclusions where reported, and any re-inclusions in analyses performed by the review authors.*** |  |
| **ITS: Was the study free from selective reporting?** | **Yes/No/Unclear** | ***State how the possibility of selective outcome reporting was examined by the review authors, and what was found.*** |  |
| **ITS: Was the study free from other risks of bias?** | **Yes/No/Unclear** | ***State any important concerns about bias not addressed in the other domains in the tool.*** |  |

* Note: For each section above ‘Yes’ indicates a ‘low risk of bias’; ‘No’ indicates a ‘high risk of bias’; ‘Unclear’ indicates an ‘uncertain risk of bias’. When entering the data into RevMan, the options to choose from will be ‘Low’, ‘High’ and ‘Unclear’

## Risk of bias assessment for All OTHER QUANTITATIVE studies (taken from Effective Public Health Practise Project – EPHPP)

**COMPONENT RATINGS**

**A) SELECTION BIAS**

**(Q1) Are the individuals selected to participate in the study likely to be representative of the target population?**

1. Very likely
2. Somewhat likely
3. Not likely
4. Can’t tell

**(Q2) What percentage of selected individuals agreed to participate?**

1. 80 - 100% agreement
2. 60 – 79% agreement
3. less than 60% agreement
4. Not applicable
5. Can’t tell

**B) STUDY DESIGN**

**Indicate the study design**

1. Randomized controlled trial
2. Controlled clinical trial
3. Cohort analytic (two group pre + post)
4. Case-control
5. Cohort (one group pre + post (before and after))
6. Interrupted time series
7. Other specify ____________________________
8. Can’t tell

**Was the study described as randomized? If NO, go to Component C.**

No Yes

**If Yes, was the method of randomization described?**

No Yes

**If Yes, was the method appropriate? __________________________**

**C) CONFOUNDERS**

**(Q1) Were there important differences between groups prior to the intervention?**

1. Yes
2. No
3. Can’t tell

**The following are examples of confounders:**

1. Race
2. Sex
3. Marital status/family
4. Age
5. SES (income or class)
6. Education
7. Health status
8. Pre-intervention score on outcome measure

**(Q2) If yes, indicate the percentage of relevant confounders that were controlled (either in the design (e.g. stratification, matching) or analysis)?**

1. 80 – 100% (most)
2. 60 – 79% (some)
3. Less than 60% (few or none)
4. Can’t Tell

**D) BLINDING**

**(Q1) Was (were) the outcome assessor(s) aware of the intervention or exposure status of participants?**

1. Yes
2. No
3. Can’t tell

**(Q2) Were the study participants aware of the research question?**

1. Yes
2. No
3. Can’t tell

**E) DATA COLLECTION METHODS**

**(Q1) Were data collection tools shown to be valid?**

1. Yes
2. No
3. Can’t tell

**(Q2) Were data collection tools shown to be reliable?**

1. Yes
2. No
3. Can’t tell

**F) WITHDRAWALS AND DROP-OUTS**

**(Q1) Were withdrawals and drop-outs reported in terms of numbers and/or reasons per group?**

1. Yes
2. No
3. Can’t tell
4. Not Applicable (i.e. one time surveys or interviews)

**(Q2) Indicate the percentage of participants completing the study. (If the percentage differs by groups, record the lowest).**

1. 80 -100%
2. 60 - 79%
3. less than 60%
4. Can’t tell
5. Not Applicable (i.e. Retrospective case-control)

**G) INTERVENTION INTEGRITY**

**(Q1) What percentage of participants received the allocated intervention or exposure of interest?**

1. 80 -100%
2. 60 - 79%
3. less than 60%
4. Can’t tell

**(Q2) Was the consistency of the intervention measured?**

1. Yes
2. No
3. Can’t tell

**(Q3) Is it likely that subjects received an unintended intervention (contamination or co-intervention) that may influence the results?**

1. Yes
2. No
3. Can’t tell

**H) ANALYSES**

**(Q1) Indicate the unit of allocation**

1. Community
2. organisation/institution
3. practice/office
4. individual

**(Q2) Indicate the unit of analysis**

1. Community
2. organisation/institution
3. practice/office
4. individual

**(Q3) Are the statistical methods appropriate for the study design?**

1. Yes
2. No
3. Can’t tell

**(Q4) Is the analysis performed by intervention allocation status (i.e. intention to treat) rather than the actual intervention received?**

1. Yes
2. No
3. Can’t tell

1. ♠ Costs associated with the intervention can be linked with provider or participant outcomes in an economic evaluation (depends on the type of economic evaluation) [↑](#footnote-ref-1)
